# Discovery of Pyrano[2,3-*c*]pyrazole Derivatives as Novel Potential Human Coronavirus Inhibitors: Design, Synthesis, In Silico, In Vitro, and ADME Studies

**DOI:** 10.3390/ph17020198

**Published:** 2024-02-02

**Authors:** Abdou K. Allayeh, Aliaa H. El-boghdady, Mohamed A. Said, Mahmoud G. A. Saleh, Mohammed T. Abdel-Aal, Mohamed G. Abouelenein

**Affiliations:** 1 Environmental Virology Laboratory 176, Water Pollution Research Department, Environment and Climate Change Institute, National Research Centre (NRC), 33 El-Behouth St., Dokki, Giza 12622, Egypt; ak.allayeh@nrc.sci.eg; 2 Chemistry Department, Faculty of Science, Menofia University, Shebin El-Kom 32511, Egypt; aliaahamed742@gmail.com (A.H.E.-b.); mtaha2000_2000@yahoo.com (M.T.A.-A.); drmoh.gamal@science.menofia.edu.eg (M.G.A.); 3 Pharmaceutical Chemistry Department, Faculty of Pharmacy, Egyptian Russian University, Cairo 11829, Egypt; mohamed-adel@eru.edu.eg; 4 Department of Chemistry, College of Science, Northern Border University, Arar 91431, Saudi Arabia

**Keywords:** synthesis, pyrano[2,3-*c*]pyrazole, COVID-19, SARS-CoV2, docking, MD, DFT, ADME

## Abstract

The SARS-CoV-2 pandemic at the end of 2019 had major worldwide health and economic consequences. Until effective vaccination approaches were created, the healthcare sectors endured a shortage of operative treatments that might prevent the infection’s spread. As a result, academia and the pharmaceutical industry prioritized the development of SARS-CoV2 antiviral medication. Pyranopyrazoles have been shown to play a prominent function in pharmaceutical chemistry and drug sighting because of their significant bioactive properties. We provide herein a novel sequence of pyranopyrazoles and their annulated systems whose antiviral efficacy and cytotoxicity were explored versus human coronavirus 229E (HCoV-229E) Vero-E6 cell lines as a model for the Coronaviridae family. Fifteen synthetic congeners pointed out miscellaneous antiviral efficacies against HCoV-229E with variable inhibition degrees. Compound **18** showed a high selectivity index (SI = 12.6) that established spectacular inhibitory capacity against human coronavirus 229E. Compounds **6**, **7**, and **14** exposed moderate efficacies. Compounds **6**, **7**, **14**, and **18** exhibited substantial antiviral action through the replication phase with reduction percentages extending from 53.6%, 60.7%, and 55% to 82.2%, correspondingly. Likewise, when assessed to the positive control tipranavir (88.6%), the inhibitory efficiency of compounds **6**, **7**, **14**, and **18** versus the SARS-CoV2 Mpro provided high percentages of 80.4%, 73.1%, 81.4% and up to 84.5%, respectively. In silico studies were performed to investigate further the biological activity and the target compounds’ physical and chemical features, including molecular dynamic (MD) simulations, protein–ligand docking, ADME studies, and density functional theory (DFT) calculations. These inquiries demonstrated that this series of metabolically stable pyranopyrazoles and their annulated systems are effective human coronavirus inhibitors that inhibit the viral M^pro^ protein and may have emerged as a novel COVID-19 curative option.

## 1. Introduction

Any society’s economic progress depends greatly on its members’ health. Therefore, the scientific community attaches great importance to studying diseases and potential treatments. Among these diseases, coronavirus disease (COVID-19) is of particular concern. It belongs to the human beta coronavirus family, including MERS and SARS. Therefore, the rapid spread and mutation of the COVID-19 virus has made it a major threat. The SARS-CoV-2 virus was initially discovered in China and has since spread to multiple countries [1], threatening people worldwide. In the past, this virus was the main cause of respiratory diseases in both birds and mammals [2]. But now, coronaviruses infect the lower respiratory canals in humans, leading to the common cold [3]. Additionally, the SARS-CoV strain is the most infectious in both immune-deficient and healthy individuals with a fatality rate ranging from 10 to 30% [4,5,6]. As a result, the World Health Organization (WHO) declared the widespread spread of infectious diseases due to the common acute respiratory syndrome SARS-CoV-2 a global pandemic in 2020 [7]. In response to this globally concerning problem, researchers were required to become involved in research for an effective drug that would prevent the propagation of this virus. As SARS-CoV-2 is considered a strain of the Coronaviridae family, having a single-stranded positive-sense encased RNA and a different genomic structure from human coronaviruses such as Middle East respiratory syndrome coronavirus (MERS-CoV) and SARS-CoV. With a genome of about 30 kb, it is larger than any other known RNA virus [8,9]. Specifically, it has virulence and structural characteristics similar to SARS-CoV [10]. The only remaining treatment options for this fatal infectious disease are the symptomatic treatment of respiratory attacks and induced infections. Therefore, effective treatment is required. Likewise, several small-molecule therapies, including paxlovid, molnupiravir, and remdesivir, have been approved by the US Food and Drug Administration (FDA) for the treatment of COVID-19 [11].

**Figure 1:**
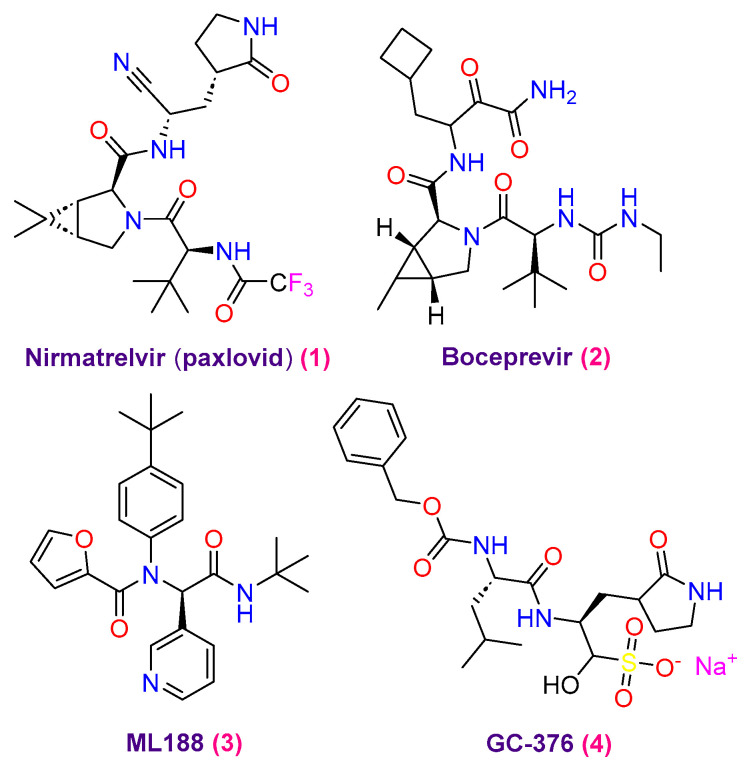
Prominent SARS-CoV-2 M^pro^ inhibitors.

However, due to the rapid appearance of powerful viral varieties, novel therapeutic identifying techniques must be developed. Targeting SARS-CoV-2 using simple inhibitors that block viral polymerases and proteases, such as papain-like protease (PL^pro^), protease (3CL^pro^ or M^pro^), and RNA-dependent RNA polymerase (RdRp), has been the focus of the researchers [12]. Hence, the inhibitors that target 3CL^pro^ and RdRp have made significant advances; several of these medications are either in studies or have received approval from the FDA [13,14,15]. Consequently, remdesivir is the main FDA-approved drug for treating severe COVID-19 symptoms within the classification of RdRp inhibitors. Consequently, remdesivir is the main FDA-approved drug for treating severe COVID-19 symptoms within the classification of RdRp inhibitors [16]. Furthermore, molnupiravir affects by starting the initiation of viral RNA alterations, which eventually prevents SARS-CoV-2 from replicating. That is why molnupiravir is classified as an RdRp inhibitor using a drug-repositioning pathway [17]. Likewise, combining inhibitors that target cytochrome P450 3A4 and 3CL^pro^, paxlovid is FDA approved for treating individuals with light COVID-19 infection, including adults and children [18]. Despite the medications’ established efficacy, certain research has revealed genetic modification worries related to paxlovid and the development of viral resistance connected to remdesivir [19]. Furthermore, there are now worries about the possible development of 3CL^pro^ mutations due to the appearance of new SARS-CoV-2 variants, among them Omicron. Such alterations could affect Paxlovid’s overall effectiveness [20]. In response to these challenges and to prevent the possible formation of SARS-CoV-2 resistance, various studies have recommended the possible use of a combined therapy treatment. Targeting other SARS-CoV-2 proteases, including PL^pro^ and 3CL^pro^, is part of this strategy [21]. Drug development prospects are enhanced by virus-encoded proteases, namely the SARS-CoV-2 major protease (M^pro^). As a cysteine protease that functions similarly to chymotrypsin, M^pro^ is essential for viral transcription and replication. Due to its functional importance, this protein is a significant target for developing potent antiviral medications. Its significance in the viral life cycle is highlighted by its function in converting polypeptides into functional proteins by cleaving polyproteins into replication-correlated proteins [22]. On the other hand, the protease’s bioefficacy is affected when the inhibitor and amino acids in the active region interact to inhibit substrate binding [23]. Consequently, creating M^pro^ inhibitors as antivirals presented several challenges for researchers. Despite these challenges, several effective compounds have been shown to bind to the M^pro^ catalytic active site, suggesting that they may be non-covalent M^pro^ inhibitors (Figure 1), including nirmatrelvir (paxlovid) (**1**), boceprevir (**2**), ML188 (**3**), and GC-376 (**4**) [24,25,26]. Therefore, it has been thought that finding the protease inhibitors for COVID-19 is an essential first step in the fight against this infectious respiratory disorder.

The FDA recently approved several vaccinations and drugs to treat COVID-19, but total viral defeat still needs to be corrected. Therefore, there is a pressing need to explore effective and safe antiviral treatments. Thus, the capability to create diverse structures to fulfill specific functions like heterocyclic compounds, abundant in nature, has been extensively utilized in pharmaceutical applications due to their versatility, which is a key factor contributing to their widespread application. Various synthetic compounds with heterocyclic moieties have become recognized for their antiviral properties [27,28]. Therefore, pyranopyrazole moieties and their annulated systems are a special structure of bioactive fused heterocyclic structures that have developed attentive significance in biochemistry due to their various pharmacological and biological efficiencies such as human Chk1 kinase inhibitors [29], antimicrobial agents [30], molluscicidal agents [31], analgesic [32], anti-bacterial [33], anti-inflammatory [22], antiproliferative [34], antifungal [35], anti-depressant [36], antioxidant [37], antimalarial [38], α-glucosidase inhibitors [39], anti-cancer [40], biodegradable agrochemicals [41], and anti-Alzheimer efficiencies [42].

The heterocyclic pyrazole and pyran frameworks have inspired several antiviral medication classes [6,43,44]. Pyran compounds were also recognized as a unique moiety for antiviral drug design [45,46]. Through in vitro testing for herpes virus HSV1 multiplication control, pyrano[2,3-*c*]pyrazoles have shown their virucide efficacies against various pathogens [47,48]. In addition, pyranopyrazoles having two aryl moieties substituted at *C*-4 and *N*-1 showed double efficacy against the developing coronaviruses and were linked to widespread inflammations [48]. By the way, we provide a challenge that uses pyrano[2,3-*c*]pyrazole surrogates as novel inhibitors to stop the spread of human coronavirus 229E infection. Our efforts can be included in reviews of the literature on the current drugs that have shown great promise in preventing the spread of viruses, such as hydroxychloroquine, ribavirin, favipiravir, oseltamivir, chloroquine, and Remdesivir [49]. Based on the previously mentioned advantages and as a component of our program aiming at investigating the synthesis of heterocyclic structures with possible biological significance [50,51], we will be concentrating on the synthesis, characterization, in silico analysis, and screening of a novel series of pyranopyrazoles and their annulated systems against the human coronavirus 229E. These substances are expected to be useful building blocks for creating novel inhibitors that target the human coronavirus’s 229E primary protease (M^pro^). This study aims to provide important new information about these substances’ potential as antiviral agents against the human coronavirus 229E.

## 2. Results and Discussion

### 2.1. Chemistry

**Scheme 1:**
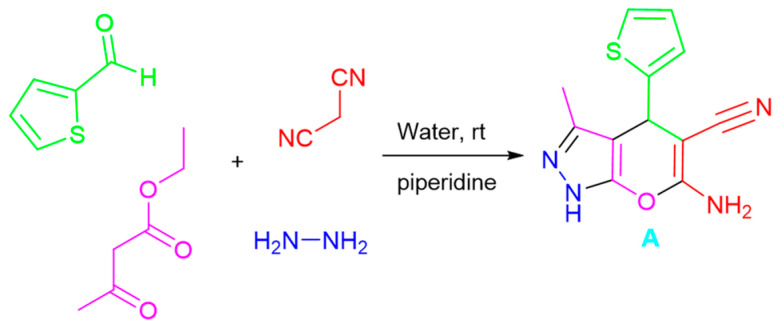
Synthesis of 6-amino-3-methyl-4-(thiophen-2-yl)-1,4-dihydropyrano[2,3-*c*]pyrazole-5-carbonitrile (**A**).

A facile multi-component technique was conducted to synthesize the enaminonitrile (**A**) through a base-catalyzed reaction using hydrazine hydrate, thiophene-2-carbaldehyde, malononitrile, and ethyl acetoacetate following the synthetic approach [51]. ^1^H, ^13^C, and infrared spectroscopy procedures verified the structure of compound (**A**). The infrared spectra showed a peak at 2187 cm^−1^ linked to the -CN group and two strong bands at 3357 and 3315 cm^−1^, corresponding to the -NH_2_ group’s stretching frequencies. The pyrazole ring’s NH proton was identified as a singlet peak at 12.14 ppm in the ^1^H NMR spectrum, which was accompanied by two singlets for the amino group protons (δ 6.94 ppm, D2O exchangeable) and δ 4.76 ppm for the C_4_-H. In the ^13^C NMR spectrum; the cyano group was detected at δ 120.45 ppm (cf. Scheme 1 and experimental part (Supplementary File)).

Derivatives of pyranopyrazoles presented an attractive point for synthesizing further heterocyclic structures. One method included refluxing **A** in DMF with 1,2,3-thiadiazole-4-carbaldehyde using a catalytic quantity of zinc chloride producing the pyrimidin-5(1H)-one derivative **1**. The carbonyl group absorption band was detected in the infrared spectrum at around 1675 cm^−1^. A broad singlet signal was seen in the ^1^H NMR spectrum at δ 11.16 ppm for the NH proton of the pyrimidin-5(1H)-one ring. Additionally, the ^13^C NMR spectra at δ 163.11 ppm showed the presence of the carbonyl group and the absence of the cyano group carbon. Moreover, refluxing compound **A** with formamide produced pyrimidin-5-amine derivative **2**. Both the IR and ^1^H NMR spectra showed the presence of the amino group, where the ^13^C NMR spectrum showed an absence of the –CN group. The fusion of 3,4-dichlorofuran-2,5-dione and compound **2** gave the pyrrolidone derivative **3**.

**Scheme 2:**
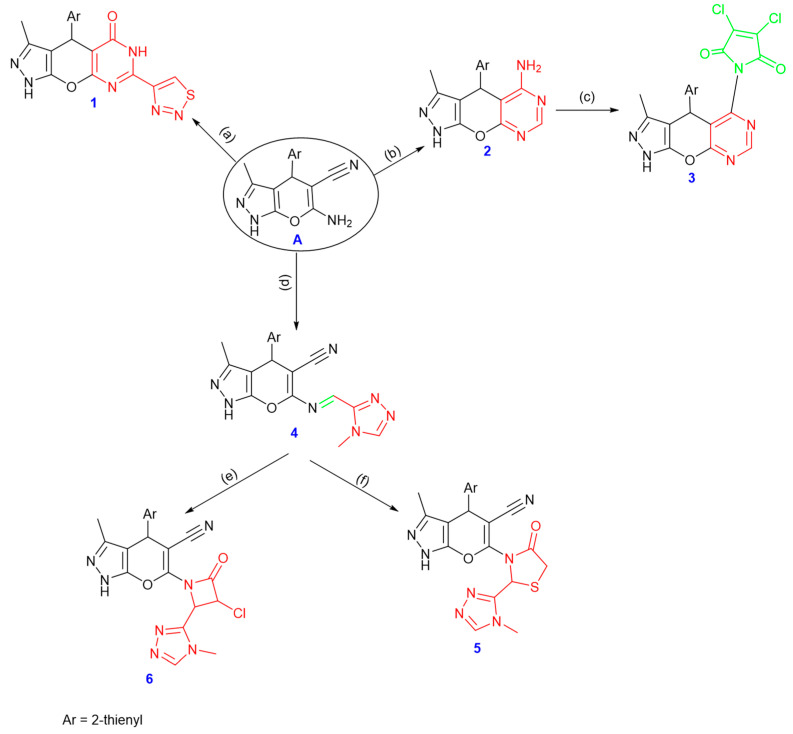
Synthesis of compounds **1**–**6**, reagents and conditions: (a) 1,2,3-thiadiazole-4-carbaldehyde, DMF, ZnCl_2_, (b) formamide, (c) 3,4-dichlorofuran-2,5-dione, (d) 4-methyl-*4H*-1,2,4-triazole-3-carbaldehyde, ethanol, reflux, (e) thioglycolic acid, (f) chloroacetyl chloride.

In addition, refluxing the starting compound **A** and 4-methyl-4H-1,2,4-triazole-3-carbaldehyde in the presence of a catalytic amount of glacial acetic acid produced the corresponding Schiff base **4**. The IR spectrum showed no absorption band related to the amino group; the NH, CN, and HC=N groups were represented by significant bands that emerged at 3345, 2220, and 1630 cm^−1^, respectively. At δ 8.58 ppm in the ^1^H NMR spectrum, a singlet signal was identified as HC=N. In addition, compound **4** refluxed with thioglycolic acid and presented compound **5**. At 1707 cm^−1^ in the IR spectrum, there was an absorption band of the carbonyl group. On the other hand, compound **4** was refluxed in DMF with a catalytic amount of triethylamine and chloroacetyl chloride to produce compound **6** (cf. Scheme 2 and the experimental section (Supplementary File)).

**Scheme 3:**
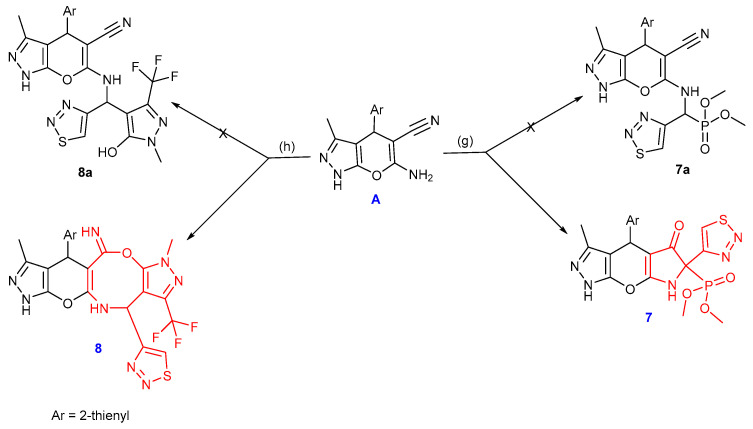
Synthesis of compounds **7** and **8**, reagents and conditions: (g) 1,2,3-thiadiazole-4-carbaldehyde, CH_3_CN, LiClO_4_, RT, (h) 1,2,3-thiadiazole-4-carbaldehyde, 1-methyl-3-(trifluoromethyl)-*1H*-pyrazol-5-ol, EtOH, ZnCl_2_.

Additionally, dimethyl(3-methyl-5-oxo-6-(1,2,3-thiadiazol-4-yl)-4-(thiophen-2-yl)-4,5,6,7-tetrahydro-1H-pyrrolo[3′,2′:5,6]pyrano[2,3-c]pyrazol-6-yl)phosphonate **7** was produced through the reaction of the enaminonitrile **A** with 1,2,3-thiadiazole-4-carbaldehyde and trimethyl phosphite in the presence of lithium perchlorate as a catalyst in acetonitrile (Scheme 3).

**Scheme 4:**
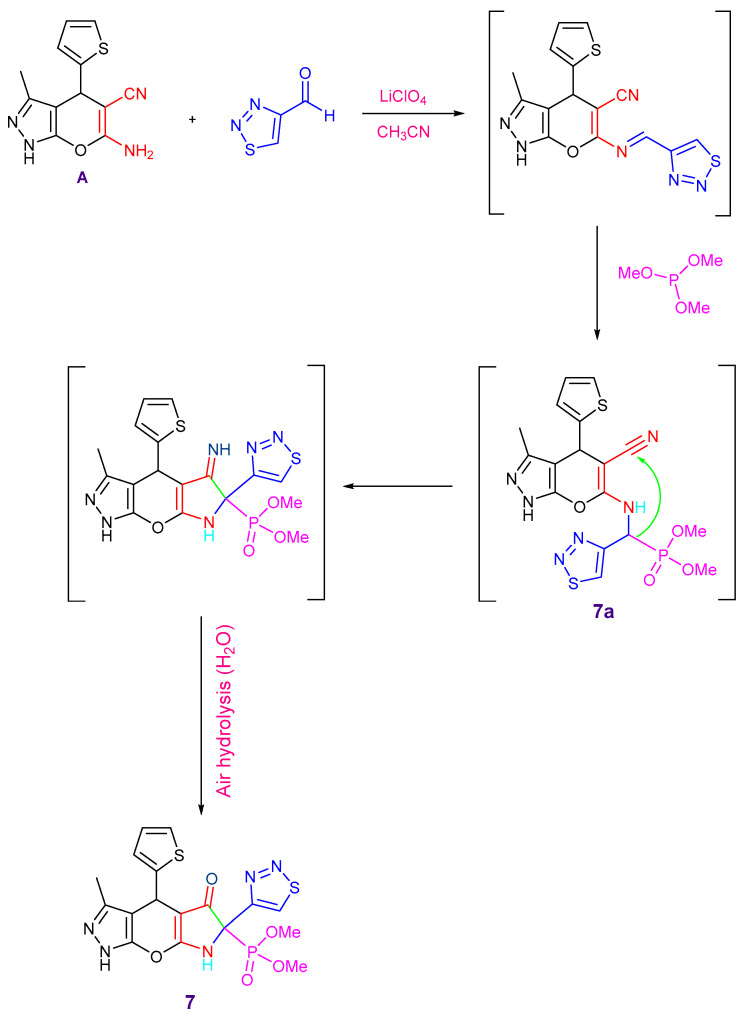
Plausible mechanism for the synthesis of compound **7**.

Initially, the Schiff base is formed by the condensation of amine **A** with the carbonyl group of 1,2,3-thiadiazole-4-carbaldehyde using LiClO_4_. When trimethyl phosphite was added, the α-aminophosphonate **7a** was produced following the Kabachnik–Field reaction. The active hydrogen of the CH–P group makes an addition to the -CN group, forming intermediate **7** through cyclization and air hydrolysis (Scheme 3 and Scheme 4). The IR spectrum revealed the C=O group absorption band at 1692 cm^−1^ and showed the CN group’s disappearance. The ^31^P NMR spectrum showed a singlet signal at δ 22 ppm, whereas the ^1^H NMR spectrum showed a singlet signal at δ 11.13 ppm related to the NH proton. With a coupling constant of (J = 149.8 Hz), the carbon atom of the C–P moiety appeared as a doublet at δ 68.75 ppm in the ^13^C NMR spectrum, while the carbonyl group signal appeared at δ 195.83 ppm. Furthermore, the mass spectrum revealed the molecular formula (C_17_H_16_N_5_O_5_PS_2_) and corresponded to the molecular ion peak at m/z 465.02 (M+, 21%). Additionally, compound **7** showed high purity (99.12%) through the HPLC chromatogram.

On the other hand, compound **8** was produced by cyclocondensation and refluxing a mixture of 1-methyl-3-(trifluoromethyl)-1H-pyrazole-5-ol, 1,2,3-thiadiazole-4-carbaldehyde, and the pyranopyrazole derivative **A** (Scheme 3). In the presence of ZnCl_2_, the reaction started with the imine formation from the reaction of compound **A** with 1,2,3-thiadiazole-4-carbaldehyde, which was followed by a Betti reaction pathway with phenol to yield compound **8a**. The hydroxyl group was then added to the cyano group with cyclization to yield compound **8**. The structure of compound **8** was elucidated by spectral analyses (cf. Experimental section, Supplementary File).

**Scheme 5:**
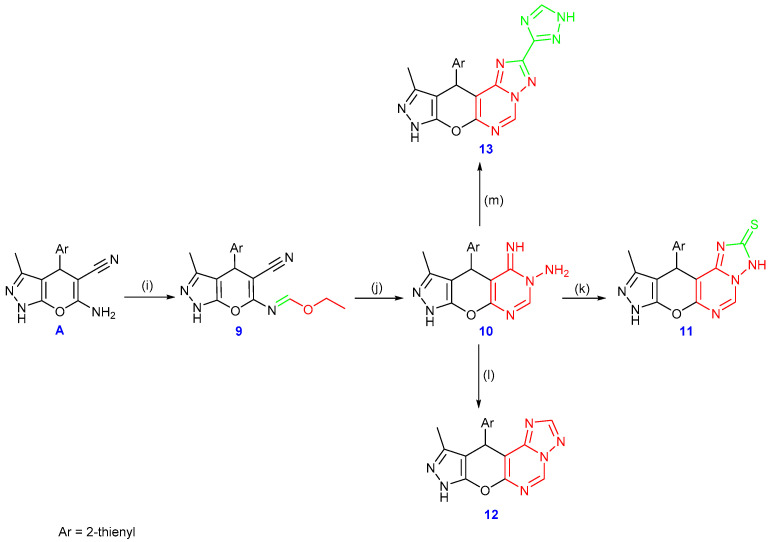
Synthesis of compounds **9**–**13,** reagents and conditions: (i) triethyl orthoformate, acetic anhydride; (j) hydrazine hydrate, (k) CS_2_, KOH, (l) formic acid, and (m) 1*H*-1,2,4-triazole-3-carbohydrazide.

Substrate A is essential for generating a range of pyrimidine derivatives mainly by converting into the iminoether derivative **9**. Subsequently, compound **A** was condensed with triethyl orthoformate to give the addition product **9**. The IR spectrum clearly showed the presence of the cyano group and the non-appearance of NH_2_. The -C=N absorption band was identified at 1638 cm^−1^. Data from ^1^H and ^13^C NMR analyses helped to verify the structure of compound **9** further. Furthermore, compound **9** was refluxed with hydrazine hydrate to form compound **10**. According to the IR spectrum, no absorption band was related to the cyano group. Three exchangeable singlet signals were found in the ^1^H NMR spectrum at δ 12.2, 9.26, and 4.83 ppm, which was related to the (–NH) and –NH_2_ groups, respectively. Also, compound **10** was refluxed with carbon disulfide, which produced compound **11**. Refluxing compound **10** with formic acid by cyclocondensation also produced the pyrimidine derivative **12**. Furthermore, upon refluxing 1H-1,2,4-triazole-3-carbohydrazide with compound **10**, triazolo[1,5-c]pyrimidine **13** was produced. The structures of compounds **11**–**13** were clarified using spectroscopic and analytical methods. The characteristic bands corresponding to the expected functional groups were detected in the IR spectra. The ^1^H, ^13^C NMR, and mass spectra further confirmed the predicted structures (cf. Scheme 5 and the experimental section in the Supplementary File).

**Scheme 6:**
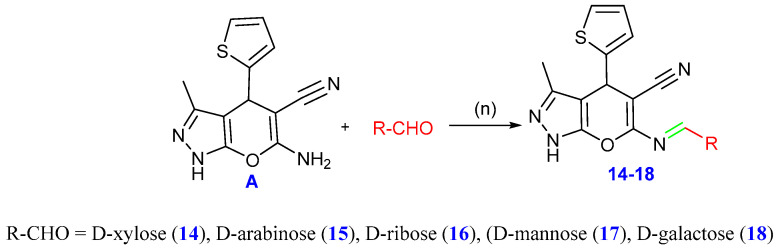
Synthesis of compounds **14**–**18**, reagents and conditions: (n) EtOH, drops of glacial AcOH, reflux.

Moreover, Schiff bases **14**–**18** were formed by the condensation of several monosaccharides with compound **A** via refluxing in acetic acid. A range of spectral data was used to determine the structures of the compounds. The infrared spectra displayed absorption bands within the 3207–3385 cm^−1^ regions related to the hydroxyl groups. The protons of the sugar moieties were identified by the signals at δ 3.6–5.42 ppm in the ^1^H NMR spectra, while the doublet peaks at δ 8.56–8.58 ppm were also correlated with signals for the aromatic protons and the protons of the condensed sugar moieties (N=CH, C-1 methine). The suggested structures were also characterized by the ^13^C NMR spectra, which revealed the signals of the sugar moieties (cf. Scheme 6 and the experimental portion in the Supplementary File). Additionally, compound **18** showed high purity (99.92%) through the HPLC chromatogram.

### 2.2. Biological Evaluation

**Figure 2:**
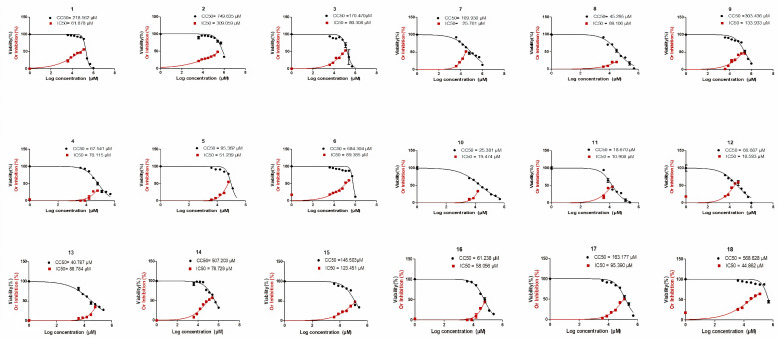
Pyrano[2,3-*c*]pyrazole congeners’ cytotoxicity and antiviral efficacy versus HCoV-229E virus.

In the current investigation, pyrano[2,3-c]pyrazoles represented an innovative category of selective and effective human coronavirus 229E inhibitors. Eighteen pyrano[2,3-c]pyrazole congeners were gradually diluted and assessed to examine their influence on Vero-E6 cell growth and survival. Utilizing the MTT test, the cell viability of the Vero-E6 cell line was estimated after incubation for 48 h. By integrating the three different experiments’ mean dose–response curves, the 50% growth inhibitory and cytotoxic doses were calculated (Figure 2).

Except for compounds (**4**, **5**, **8**, **10**, **11**, **12**, **13**, and **16**) with a CC_50_ extending from 18 to 95 μM on the vero E6 cell line, the other investigated compounds were not harmful to the Vero-E6 cell line at dosages more than 100 μM. Employing a cytopathic inhibition assay, the investigated compounds’ in vitro antiviral efficacy versus HCoV-229E was assessed with crystal violet dye. Untreated virus-infected cells were utilized in the assay as a control. Opposing HCoV-229E, the 50% inhibitory concentration (IC_50_) of the compounds was established (Table 1).

**Figure 3:**
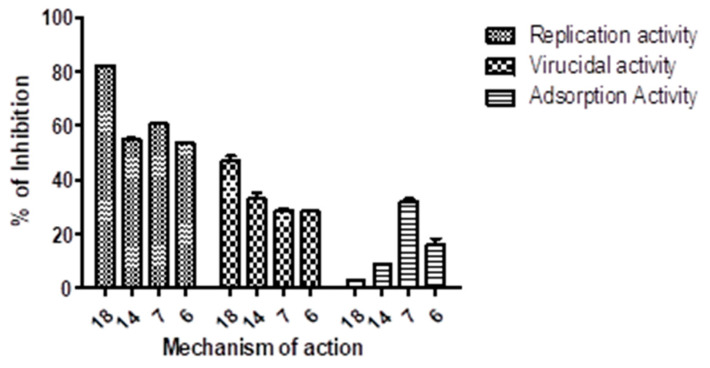
Pyrano[2,3-*c*]pyrazoles (**6**, **7**, **14**, and **18**) inhibitory mechanism mode versus minimal morbific coronavirus 229E. Utilizing the plaque reduction assay, the three principal mechanisms were evaluated: virucidal, adsorption, and replication.

Compound **18**, with a selectivity index of (12.6 M) successfully decreased HCoV-229E, making it a potential therapy for human HCoV-229E. The human HCoV-229E virus was suppressed by compounds **6**, **7**, and **14** with moderate selectivity indices of 7.6, 4.3, and 6.5 M, respectively. Compounds **1**, **2**, **3**, **5**, **9**, **10**, **11**, **12**, **15**, **16**, and **17** had minimal antiviral activity; however, compounds **4**, **8**, and **13** did not. The plaque assay, on the other hand, was used to determine the inhibitory effect mode of action for compounds with high or moderate activity. Compounds **18**, **14**, **7**, and **6** exposed strong antiviral efficacies throughout the replication process with reduced percentages of up to 82.2%, 55%, 60.7%, and 53.6%, respectively. During the adsorption procedure, the same chemicals demonstrated no antiviral action. The four compounds had modest antiviral activity ranging from 28% to 42% (Figure 3).

**Figure 4:**
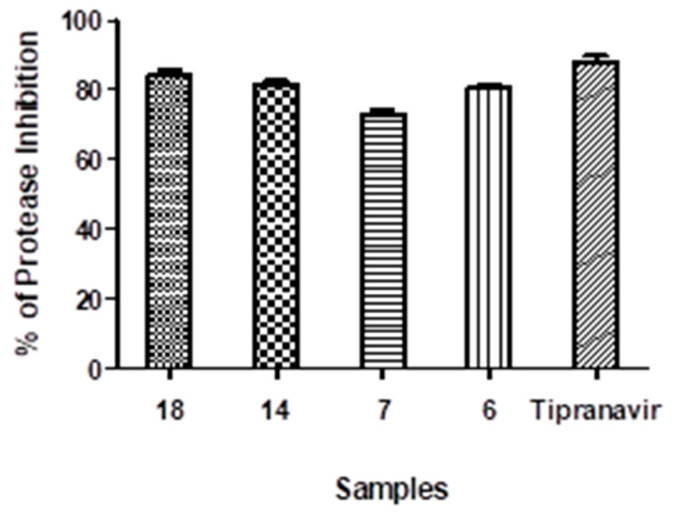
Inhibition effect of pyrano[2,3-*c*]pyrazoles (**6**, **7**, **14**, and **18**) on protease enzyme of SARS-CoV2.

Moreover, we compared the four surrogates’ impacts to a positive control (Tipranavir) on inhibiting the SARS-CoV2 protease enzyme. Compounds **6**, **7**, **14**, and **18** have IC_50_ values of 44.78, 359.5, 70.3, and 27.8 μg/mL, respectively, compared to tipranavir’s IC_50_ of 13.32 μg/mL. Furthermore, the four investigated compounds’ inhibitory efficiency versus the SARS-CoV2 protease enzyme was 80.4%, 73.1%, 81.4%, and 84.5%, respectively, when compared to the positive control tipranavir, which inhibits up to 88.6% at 100 μg/mL (Figure 4). This implies that compound **18**, which has no toxicity on cells and a high selectivity index of up to 12.6 μM in primary antiviral testing, hinders with the greatest percentage of 84.5% compared to the positive control inhibition percentage of 88.6% for the SARS-CoV2 protease enzyme. This suggests that this pyranopyrazole congener is a SARS-CoV2 potential antiviral medication.

### 2.3. Computational Studies

#### 2.3.1. Docking Simulations

**Figure 5:**
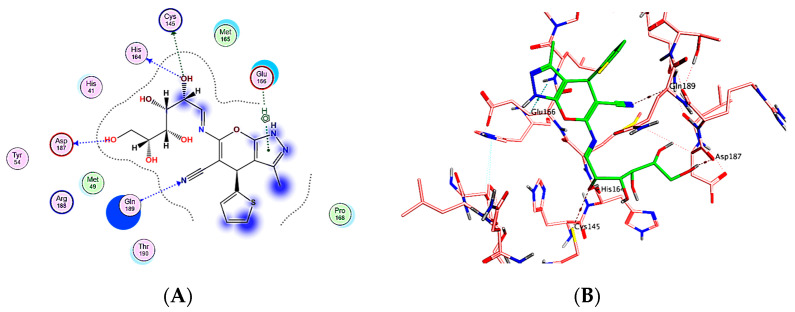
(**A**) Two-dimensional (2D) and (**B**) 3-D interaction docking poses for compound **18**.

**Figure 6:**
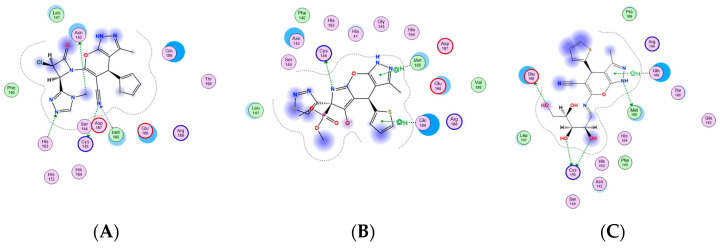
Two-dimensional (2D) interaction docking poses for (**A**) compound **6**, (**B**) compound **7**, and (**C**) compound **14**.

Compounds **6**, **7**, **14** and **18** were selected as the most selective and effective antiviral inhibitors for the SARS-CoV2 Main protease enzyme (M^pro^) [PDB id: 6LU7]. Molecular operating environment (MOE-2019.0102.) software was exploited in preparing either the enzyme or target compounds (**6**, **7**, **14** and **18**) in addition to the docking process of the previously mentioned compounds into the viral protein active site. The resulted outcomes revealed that compound **18** is the most probable effective antiviral inhibitor for the main protease enzyme, as it showed the best binding score in addition to the best interaction pose, that include four hydrogen bond in addition to one hydrophobic-binding interaction with the enzyme key amino acid residues His164, Gln189, Cys145, Asp187 and Glu166, respectively (Figure 5) [52,53]. Meanwhile, compounds **6** and **14** showed moderate enzyme inhibitory effect with suitable binding interaction to the key amino acid residues and moderate binding score, as shown in Figure 6. Finally, compound **7** was the worst of the library, as it revealed as having the lowest binding score with one hydrogen bond and two hydrophobic interactions the enzyme (Figure 6). It was observed that the docking results including scores and binding interaction were matched with the previously tested IC_50_ values, which validate the docking protocol and results (Table 2).

#### 2.3.2. Molecular Dynamic Simulations

**Figure 7:**
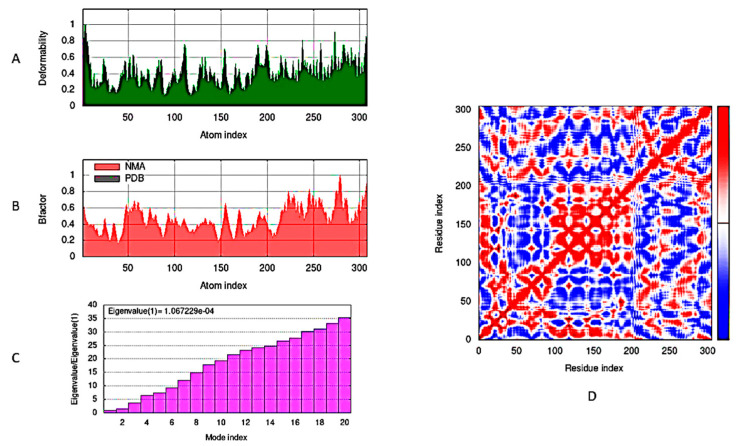
Compound **18**/M^pro^ complex molecular dynamics simulation by iMODS server: (**A**) Deformability, (**B**) B−factor values, (**C**) eigenvalue, and (**D**) covariance model.

In Figure 7, the docked complex of viral M^pro^ enzyme and compound **18** were simulated using molecular dynamics (MD) and normal mode analysis (NMA) of iMod server (iMODS: http://imods.chaconlab.org/, accessed on 28 June 2023). A computational investigation was carried out for determining the flexibility of M^pro^ residues with respect to the target compound **18**. The main-chain deformability graph shown in (Figure 7A) is illustrating that the higher peaks represent the protein deformability regions, indicating the flexibility and relocation of group of amino acid resides to improve their binding to the target compound **18**. The experimental B-factor in (Figure 7B) determines the root mean square (RMS) value and illustrates the protein complex atoms’ unpredictability. The enzyme-compound **18** complex obviously had a relatively low eigenvalue of 1.067229 × 10^−4^, which reveals the ability of high deformability and degree of M^pro^ flexibility (Figure 7C). The covariance map (Figure 7D) amongst the ligand and enzyme residues is displayed in Figure 7C, signifying their high connections (anti-correlated motion shown via blue color, interrelated motion specified shown through red color, and uncorrelated motion directed with white color).

#### 2.3.3. Swiss-ADME Study

**Figure 8:**
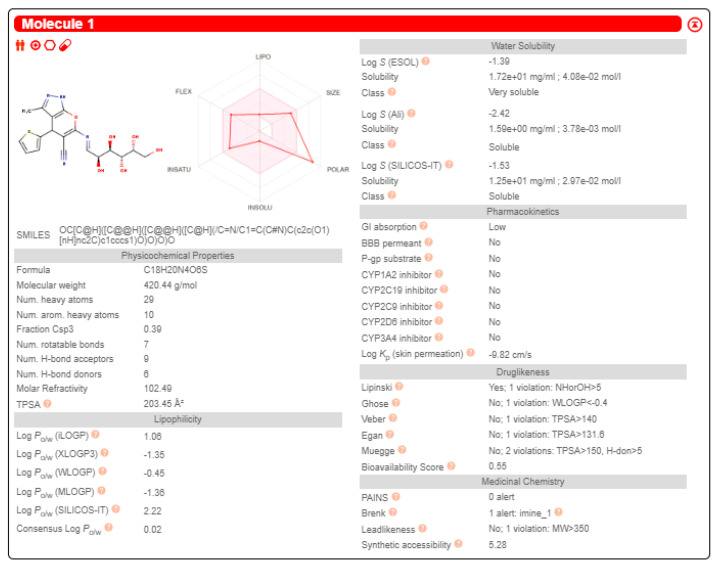
Representing compound **18** Swiss−ADME outcomes.

Compound **18**’s ADME properties and physicochemical features (solubility, lipophilicity, polarity, size, flexibility, and saturation) were predicted using the Swiss-ADME web tool (http://www.swissadme.ch, accessed on 28 June 2023). The target compound **18** exhibited less lipophilic (LogP), soluble, and polar (TPSA) features. It has low GIT absorption without CNS side effects (cannot cross the BBB). There were no PAINS structural toxicity alerts [54]. The results assumed its ability to inhibit non-metabolizing enzymes (CYP2C9, CYP1A2, CYP2C19, CYP3A4, CYP2D6), indicating the hepatotoxicity’s non-existence, but it disclosed BRENK structural toxicity alerts due to the imine group existence. It was demonstrated that additional scaffold adaptations are seriously required in the future to improve oral bioavailability (Figure 8).

#### 2.3.4. Density Function Theory (DFT)

##### Structure Optimization

**Figure 9:**
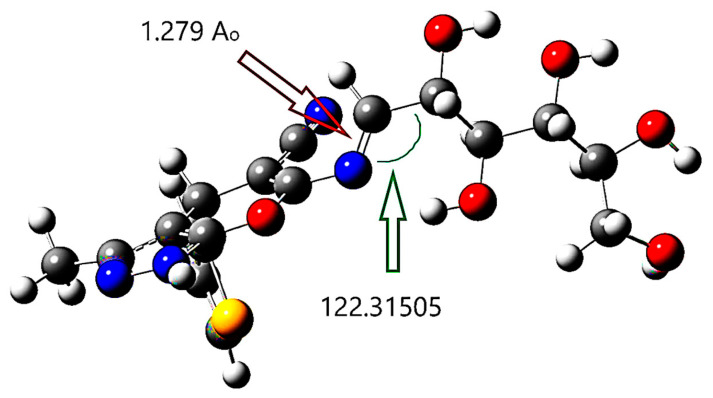
The selected compound’s (**18**) ground state optimized molecular structure at B3LYB/6-31++G(d,p).

The DFT/B3LYP technique was used to optimize the structure of compound **18**. This structure is formed via condensing D-galactose monosaccharide moiety with the β-enaminonitrile **A** amino group. The imine group replaces the carbonyl group. In Figure 9, the optimized structure of compound **18** is summarized. The imine bond length was exposed in DFT calculations to be 1.279 Å; however, the angle positioned on its side was found to be 122.31505°.

##### Frontier Molecular Orbital Analysis

**Figure 10:**
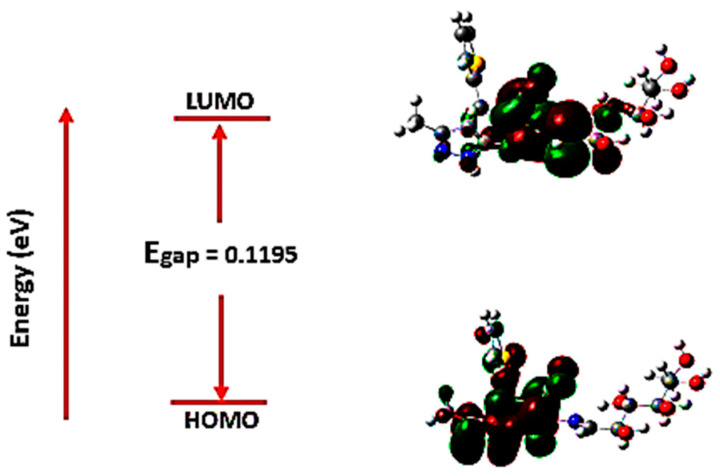
Frontier molecular orbitals: ground state HOMO and LUMO at B3LYB/6-31++G(d,p).

The molecular orbital energies are started as an excellent means for describing a molecule’s optical and electric properties using quantum chemistry. Frontier molecular orbitals (FMOs) are the highest occupied molecular orbital (HOMO) and the lowest unoccupied molecular orbital (LUMO) located at the molecules’ electrons’ outermost borders defining the conjugated molecules; FMO successfully explained the electron excitation from (HOMO) to (LUMO). HOMO energy (EHOMO) is used to estimate the electron–donor essence, whereas LUMO energy (ELUMO) is exploited in the electron–acceptor essence estimating. Correspondingly, lower LUMO and higher HOMO values match lower electron-acceptance resistance and electron–donor capacity. In Figure 10, the frontier molecular orbitals are exhibited where the molecule’s negative and positive phases are denoted as red and green-colored regions.

In Figure 10, HOMO is restricted to most of the atoms excluding some hydrogen and the sugar moiety atoms, while LUMO is delocalized only upon the molecule middle region. Employing the FMO essential energy gap value (Egap), the molecules’ stability toward succeeding chemical reactions is indicated. The numerical values of Egap in addition to other target molecule electronic parameters were computed (Table 3). Established upon Koopmans’ theory, the target molecule’s chemical reactivity descriptors like ionization potential (IP), chemical potential (µ), electronegativity (-χ), electron affinity (EA), global chemical hardness (դ), electrophilicity (Ѡ), and global chemical softness (S), were assessed [55].

Table 3 lists all the computed reactivity and energetic parameters, including the dipole moment (Dm) of the target compound **18**. It was obvious that the E_gap_ among HOMO and LUMO is calculated to be 0.1195 eV (modest) [55]. The trivial frontier orbital gap value for a compound indicates a chemical reactivity and an extreme polarizable; such finding explicates compound 18 antiviral embarrassment capability in contradiction of human Coronavirus 229E and the main protease enzyme M^pro^. The molecule’s chemical responsiveness concept is strongly tied to theoretical chemistry constructed on the FMO theory.

## 3. Materials and Methods

### 3.1. Chemistry

Detailed methodology and experimental data were provided in the Supplementary Materials.

### 3.2. Biological Assay

#### 3.2.1. Viruses and Cells

Cell lines and viral types were kindly provided through the Nawah-Scientific Centre (Egypt) and employed from the American Type Culture Collection (Manassas, VA, USA). For propagating human coronavirus 229E (HCoV-229E), an African green monkey kidney cells (Vero-E6) clone was utilized. In DMEM medium-high glucose with 1% antibiotic solution and 10% FBS (Gibco BRL, New York, NY, USA), the cells were grown up.

#### 3.2.2. MTT Assay for Cellular Toxicity

In 96-well plates, a monolayer of a Vero-E6 cell line was incubated after its introduction at 1 × 10^5^ cells for every well and in 5% CO_2_ to assess cytotoxicity at 37 °C. After removing the growing media, each substance examined was diluted (two-fold) in 0.01 mL of medium and cultured at 37 °C in a 5% CO_2_ atmosphere for 24 h. Three times, every compound was investigated. The usual control cells received no treating compounds. Following 24 h of treatment, each well was incubated for 2 h at 37 °C after receiving 20 L of 5 mg/mL MTT. After that, the medium should be progressively eliminated. For 15 min, the cells were shaken on an orbital shaker after wrapping them with tin foil. Utilizing a microplate reader at 570 nm, the optical density was restrained. The cell’s vitality was established as the cell controls’ mean optical density percentage, which was set at 100%. Utilizing the GraphPad PRISM program, the mean dose–response curves determined the 50% cytotoxic concentrations (CC_50_) [56].

#### 3.2.3. Cytopathic Inhibition Assay

The examined compounds’ antiviral efficacy is largely judged microscopically; however, in this experiment, crystal violet was employed in producing supplemental tangible findings. Resembling the cytotoxic assay, the CPE inhibitory experiment employed a Vero-E6 cell line upon its execution in 96-well plates. Supporting the cell growth medium aspiration, a coefficient multiplicity of infection (MOI) = 1 of HCoV-229E (1 × 10^5^ TCID_50_) and 100 µL of every pyranopyrazole diluted in the medium were instituted to the wells in 100 µL of the suitable medium. Either infected or non-infected cells devoid of compounds were employed as viral and cell controls. In 5% CO_2_ at 37 °C, plates have been placed. The course of the cytopathic impact was monitored using inverted microscopy. Using the same method as for the cytotoxicity experiments, the cells fixing and coloring with a 0.03% crystal violet solution in 3% formalin and 2% ethanol were executed in water. To compute the antiviral efficiency percentage of any substance, we use the subsequent formula: antiviral efficiency = [(cell controls’ mean optical density minus virus controls)/(the test optical density minus virus controls)] × 100. Based on these data, the 50% CPE inhibitory dose (ID_50_) was calculated [16,57].

#### 3.2.4. Calculating IC_50,_ CC_50_, and SI Values

The antiviral efficiency 50% inhibitory concentration (IC_50_) calculation was executed by plotting the investigated compounds’ successive dilutions’ optical density vs. the compounds’ concentration. Utilizing the cells’ average vitality relating to the investigated compound concentration, the 50% cytotoxic impact (CC_50_) was determined. These values were calculated based on linear regression analysis, and the GraphPad Prism software program v.5. CC50/IC50 was used to calculate the selective index (SI).

#### 3.2.5. Mode of Action Using Plaque Assay

Plaque assay was used, as previously described, to assess if the candidate compounds have a virucidal impact through the virus life cycle on the viral particle and adsorption and/or reproduction [58].

#### 3.2.6. Virucidal Mechanism

Vero E6 cells were cultured inside a six-well plate for one day at 37 °C. For 30 min, the virus was incubated with the sample in varied doses (as much as the untreated viral control). After the growth medium removal of the cell culture plates, countable sample/virus mixtures (100 µL/well) were supplemented into the cells. After 1 h of contact, the cell monolayer was provided with 2% agarose assorted with 1.5 mL of DMEM; plate hardening was permitted before incubation at 37 °C until forming the plaques. After a 2 h immersion in 10% formalin, the plates were stained in distilled water with 0.1% crystal violet. Vero E6 cells were cultivated in control wells with untreated virus, counting the plaques and reporting the plaque reduction percentage likened with control in the following manner: % inhibition = viral count (untreated) minus viral count (treated) divided by viral count (untreated) multiplied by 100.

#### 3.2.7. Adsorption Mechanism

During this process, the identical methods as in the previous section were carried out on a six-well plate, except the plates’ growth medium withdrawing and infecting the investigated compounds with varied concentrations (100 µL/well). After 1 h of incubation, the virus (100 µL/well) was inserted. Afterward 1 h of interaction, the cell monolayer was provided with 2% agarose mixed with 1.5 mL of DMEM, and the plate hardness was permitted before incubation at 37 °C until formation of the viral plaques.

#### 3.2.8. Replication Mechanism

The same actions as in the previous section were performed in a six-well plate during this mechanism except for the cell culture plates growth medium amputation and the virus introduction (100 µL/well) and incubation for 1 h at 37 °C subsequent to the infected cells injection with the investigated compound in erratic concentrations (100 µL/well). The cell monolayer was provided with 2% agarose assorted with 1.5 mL of DMEM after 1 h of interaction; plate hardening was permitted before incubating at 37 °C until forming the viral plaques.

#### 3.2.9. Protease (SARS-CoV2) Inhibition Test

The Protease (SARS-CoV2) inhibition test was executed using the 3CL Protease Test Kit (Catalogue Number #79955-1; BPS Bioscience, San Diego, CA, USA). The 3CL protease, known as the Main Protease (M^pro^), is essential in digesting polypeptides translated from viral RNA [59,60]. Viral replication can be thwarted through 3CL Protease inhibitors. The quantitative component of the protease test was performed using micro-well strips as directed by the manufacturer. In short, the wells characterized as the “inhibitor control”, “positive control”, and “test compound” were provided with 30 µL of diluted 3CL Protease enzyme solution (M^pro^). The reaction started with 10 µL of substrate solution in every well, which was followed by overnight ambient incubation. The fluorescence intensity was then estimated utilizing a micro-titer plate-reading fluorometer equipped for detection at 460 nm and excitation at 360 nm.

#### 3.2.10. Statistical Investigation

In triplicate, whole experiments were executed, and all computations were performed with GraphPad PRISM and linear regression analysis (Version 5.0.1, GraphPad Software, San Diego, CA, USA).

### 3.3. Computational Investigations

#### 3.3.1. Docking Simulations

Molecular operating environment (MOE: 2019.0102) software was manipulated for preparing the ligand and protein, molecular docking studies, and evaluating ligand–protein interaction via pose visualization and scoring function [61,62]. Rescoring: London dG, docking placement: triangular matcher, refinement: Affinity dG, and force field were used in the docking process via the MMFF94x protocol.

#### 3.3.2. The Target Protein Structure Preparation

The main protease (M^pro^) as a significant SARS-CoV2 viral protein playing a decisive role through evolving research in anti-CoV potential drug design was repossessed with PDB id: 6LU7 from the Protein Data Bank (www.rcsb.org) [63]. MOE’s automatic correction was used for checking and restoring the protein structure. Throughout the protonation step, the hydrogen atoms were supplemented into the structure. For determining the binding site, the N3 ligand was used. The bounded ligand N3 and the co-crystallized water molecules were then removed. Following the docking accomplishment, the outcomes were observed and filtered via binding scores and visualized poses.

#### 3.3.3. The Investigated Drug Molecules’ Preparation

MOE was exploited in preparing the 3D model library from the selective and effective target pyrano[2,3-*c*]pyrazoles (**6**, **7**, **14**, and **18**). The compounds were exposed to an energy-minimizing method as well as automated partial charge computation. Finally, this constructed library was saved as an mdb file for docking against the main protease enzyme.

#### 3.3.4. Docking Validation

The docking process is validated via computing the root mean square deviation (RMSD). The RMSD is calculated after redocking the co-crystallized ligand on its target enzyme, and the redocked co-crystallized ligand is superimposed on the inventive co-crystallized constrained conformation. During this study, the RMSD of M^pro^ (6LU7) looked to be in the appropriate range (1.8 Å).

#### 3.3.5. Molecular Dynamic Simulations

The remarkable posed docking complex (Enzyme-compound **18** complex) and nominated lowermost energy esteemed were investigated by a molecular dynamic simulation approach based on the docking data. The iMod server (iMODS) (http://imods.chaconlab.org/, accessed on 28 June 2023) ran the MD simulations in inner coordinates at 1 atm constant pressure and 300 K constant temperature [64,65] to provide a user-friendly boundary for this improved normal mode analysis (NMA) methodology. Finally, the target complex was investigated by molecular dynamics simulation for 50 ns.

#### 3.3.6. Swiss-ADME Study

The Swiss-ADME platform (http://www.swissadme.ch/, 6 July 2023) is a liberally obtainable web implement that assembles the maximum significant computational techniques for providing universal estimates of the pharmacokinetics profile of the small molecules. Because of their robustness and ease of interpretation, the web tool creators chose their methodologies to allow effective interpretation into medicinal chemistry. Some methodologies were created by web tool developers operating open-source algorithms, while others were unchanged versions of the approaches created through the original authors [66]. The target compound **18**’s molecular structure was submitted into the Swiss-ADME web tool segment applying the simplified molecular-input line-entry specification (SMILES) labelling technique, and the result report was generated expending Marvin sketch software 19.19.

#### 3.3.7. Density Function Theory (DFT)

The DFT quantum chemistry calculations were executed employing the Gaussian 09 program. All the data files were displayed using GaussianView6 [55]. To optimize the organic chemical structure of compound **18**, the density function theory (DFT) at 6-31G++(d,p) basis set/B3LYP method was applied, and Avogadro software 1.2.0 was used to generate and optimize the original chemical structure [67,68].

The global chemical hardness (դ), ionization potential (IP), electrophilicity (Ѡ), global chemical softness (S), chemical potential (µ), electron affinity (EA), and electronegativity (χ) are Koopmans’ theory equations.

Energy gap = |E_HOMO_ -E_LUMO_| eV

Dipole Moment (Dm) Debye

Ionization Potential (IP = −E_HOMO_) eV

Electron Affinity (EA = −E_LUMO_) eV

Electronegativity χ = (I + A)/2 eV

Chemical Potential µ = (IP + EA)/2

Global Chemical Hardness դ = (IP − EA) eV

Global Chemical Softness σ = 1/դ

Electrophilicity Index Ѡ = µ^2^/2դ eV

## 4. Conclusions

In the existing investigation, we have introduced innovative pyranopyrazoles and their annulated systems, elucidating their structures by an arrangement of spectral performances. The synthesized congeners were assessed for their antiviral efficiency and cytotoxicity against human coronavirus 229E as a model for the Coronaviridae family. The cytotoxicity of pyranopyrazole compounds was exhibited to be least against the Vero-E6 cell lines. Pyranopyrazoles and their annulated systems demonstrated miscellaneous antiviral efficacies. Fortunately, compound **18** achieved the extreme selective index (12.6) and could efficiently inhibit human coronavirus 229E. The most active compounds **6**, **7**, **14**, and **18** were screened for the SARS-CoV2 M^pro^ inhibitory assay to evaluate their potential antiviral mechanism. Representing an IC_50_ value of 27.8 μg/mL, compound **18** compellingly hampered the M^pro^ efficacy associated with tipranavir’s IC_50_ of 13.32 μg/mL. The docking results showed that compound **18** is the most active antiviral inhibitor for the main protease enzyme showing good docking binding scores and interactions that matched the tested IC_50_ values. Moreover, MD simulations revealed the flexibility and relocation of a group of amino acid residues for improving their binding to the target compound **18**. However, the ADME study revealed the need for additional future modifications to the chemical scaffold in order to improve oral absorption ability. Finally, the density functional theory application explained the antiviral inhibition ability of compound **18** against human Coronavirus 229E as a model for the Coronaviridae family and SARS-CoV2 main protease (M^pro^) enzyme.

## Figures and Tables

**Table 1:** The selective index (SI) and inhibitory concentration (IC_50_) of the investigated compounds.

Compound Code	IC_50_ (µM)	SI (µM)
**1**	61.878	3.5
**2**	309.059	2.4
**3**	80.308	2.1
**4**	70.115	0.9
**5**	51.239	1.86
**6**	89.385	**7.6**
**7**	25.761	4.3
**8**	69.106	0.6
**9**	133.933	2.3
**10**	19.474	1.3
**11**	10.908	1.7
**12**	18.593	3.5
**13**	88.784	0.46
**14**	78.729	**6.5**
**15**	123.451	1.18
**16**	58.056	1.05
**17**	95.390	1.7
**18**	44.862	**12.6**

**Table 2:** The target compounds’ featured interaction and docking scores against M^pro^ enzyme.

Compound	M^pro^ % Inhibition	S-Score (kcal/mol)	Involved Receptor Residues	Type of Bond Interaction
**6**	80.4%	−6.1855	Met165 Cys145 His163 Asn142	H-bond H-bond H-bond H-bond
**7**	73.1%	−5.8237	Cys145 Met654 Gln189	H-bond Hydrophobic Interaction Hydrophobic Interaction
**14**	81.4%	−6.5673	Met 165 Glu166 Cys145 Gln189	H-bond H-bond H-bond Hydrophobic Interaction
**18**	84.5%	−7.3091	His 164 Gln189 Cys145 Asp187 Glu166	H-bond H-bond H-bond H-bond Hydrophobic Interaction

**Table 3:** Compound **18** DFT calculated energetic parameters and global reactivity.

Parameters	Compound 18
E_HOMO_	−0.29094
E_LUMO_	−0.17144
Energy Gap (E_gab)_	0.1195
Dipole Moment (Dm)	2.2848697
Ionization Potential (IP)	0.29094
Electron Affinity (EA)	0.17144
Electronegativity (χ)	−0.1195
Chemical Potential (µ)	0.23119
Global Chemical Hardness (դ)	0.1195
Global Chemical Softness (σ)	8.2682
Electrophilicity Index (Ѡ)	0.223635

## Data Availability

Data is contained within the article and Supplementary Material.

## Supplementary Materials

The following supporting information can be downloaded at https://www.mdpi.com/article/10.3390/ph17020198/s1, Chapter S1. Material and methods (3.1. Chemistry section). Chapter S2. Characterization details (IR, Mass, HNMR and CNMR) for the synthesized compounds. Chapter S3. Computational studies (docking, MD, ADME, DFT).
