# Supplementary material for: Discovery of Pyrano[2,3-c]pyrazole Derivatives as Novel Potential Human Coronavirus Inhibitors: Design, Synthesis, In Silico, In Vitro, and ADME Studies
Source: Pharmaceuticals (Basel). 2024 Feb 2;17(2):198. doi: 10.3390/ph17020198 (PMC10892497; doi:10.3390/ph17020198)
Supplement: Supplementary file 1 [file pharmaceuticals-17-00198-s001.zip › pharmaceuticals-2752707-supplementary.pdf]

3. Materials and Methods

3.1. Chemistry

The whole substances were utilized with no additional purification after importing by mercantile suppliers and. Thin-layer chromatography (TLC) was executed utilizing UV light at 254 nm established on silica gel 60 F254 precoated plates (Merck); they were envisioned to monitor both the tested compounds' purity and the reactions' progress. By the open capillary procedure, on the Stuart science melting point system, the melting points were gauged and uncorrected. On the JEOL ECA-500 II spectrometer (500 MHz and 100 MHz, correspondingly), wholly ^{1}H NMR as well as ^{13}C NMR spectra were carried out at the NMR unit, Mansoura University. In δ part per million (ppm), chemical shifts are recorded comparatively to tetramethylsilane (δ 0 ppm). On Thermo Fisher Nicolet IS10, USA spectrometer (Mansoura University), Egypt, the IR spectra were performed, and utilizing a 70 eV GC/MS SHIMADZU spectrophotometer at the Microanalytical centre (Al-Azhar University), mass spectra were reported and recorded. An automated analyzer (CHNS Vario EL III-Elementary Analyzer) was employed at the Microanalytical centre (Al-Azhar University) in documenting all the resulting compounds' CHN elemental analysis.

Synthesis 6-amino-3-methyl-4-(thiophen-2-yl)-1,4-dihydropyrano[2,3-c]pyrazole-5-carbonitrile (A)

The *β*-enaminonitrile (**A**) was initially developed by condensing hydrazine hydrate, thiophene-2-carbaldehyde, ethyl acetoacetate, and malononitrile consistent with the stated procedure [41].

3-methyl-7-(1,2,3-thiadiazol-4-yl)-4-(thiophen-2-yl)-4,6-dihydropyrazolo[4',3':5,6]pyrano[2,3-d]pyrimidin-5(1H)-one (1)

A mixture of pyranopyrazole derivative **A** (2 mmol, 0.52 g), 1,2,3-thiadiazole-4-carbaldehyde (2 mmol, 0.23 g), and zinc chloride (20% mol) as a catalyst was refluxed in DMF until the reaction completion (TLC). The mix was chilled and transferred into icy water. The solid was isolated via filtration, desiccated, and recrystallized exploiting ethyl alcohol as a white powder: Yield 88%; m.p: 217-219 °C; IR (KBr) cm^{-1} 3407, 3230, 3090, 2965, 1675, 1632, 1605, 1198; ^{1}H NMR (500 MHz, DMSO-d_6)δ(ppm): 12.2 (s, 1H), 11.16 (s, 1H), 8.17 (s, 1H), 7.41-7.42 (d, 1H, *J*= 2 Hz), 6.24-6.25 (m, 1H), 6.06-6.1 (d, 1H, *J*= 2.5 Hz), 5.2 (s, 1H), 2.08 (s, 3H); ^{13}C NMR (100 MHz, DMSO-d_6)δ(ppm): 163.11, 158.31, 156.52, 155.07, 152, 143.67, 143.42, 136.87, 132.5, 112, 107.42, 100.13, 99.41, 30.83, 10.67; EIMS, *m/z* [M]$^+$ calcd: 370.03; found: 370.02; Elemental Analysis for $C_{15}H_{10}N_6O_2S_2$ (%), Calcd: C, 48.64; H, 2.72; N, 22.69; found: C, 48.69; H, 2.67; N, 22.64.

3-methyl-4-(thiophen-2-yl)-1,4-dihydropyrazolo[4',3':5,6]pyrano[2,3-d]pyrimidin-5-amine (2)

A solution of pyranopyrazole derivative **A** (3 mmol, 0.77 g) in surplus of formamide (10 mL) was refluxed for 5 h till the reaction end up. Afterward cooling, the mix was deflated over cold water, and the solid was filtered off, and recrystallized utilizing ethyl alcohol as a yellow powder: Yield 86%; m.p: 256-258 °C; IR (KBr) cm^{-1} 3410, 3245, 2924, 1630, 1591; ^{1}H NMR (500 MHz, DMSO-d_6)δ(ppm): 12.19 (s, 1H), 8.35 (s, 1H), 7.608-7.612 (d,1H, *J*= 2 Hz), 7.04 (s, 2H), 6.36-6.37 (m, 1H), 6.17-6.18 (d, 1H, *J*= 2.5 Hz), 5.21 (s, 1H), 2.16 (s, 3H); ^{13}C NMR (100 MHz, DMSO-d_6)δ(ppm): 163.64, 159.11, 157.21, 155.82, 152.91, 143.09, 136.54, 112.38, 107.81, 100.54, 99.62, 31.24, 10.34; EIMS, *m/z* [M]$^+$ calcd: 285.07; found: 285.07; Elemental Analysis for $C_{13}H_{11}N_5OS$ (%), Calcd: C, 54.72; H, 3.89; N, 24.55; found: C, 54.68; H, 3.85; N, 24.49.

3,4-dichloro-1-(3-methyl-4-(thiophen-2-yl)-1,4-dihydropyrazolo[4',3':5,6]pyrano[2,3-d]pyrimidin-5-yl)-1H-pyrrole-2,5-dione (3)

An equimolar amount of compound **2** (2 mmol, 0.57 g) and 3,4-dichlorofuran-2,5-dione (2 mmol, 0.33 g) in existing of freshly fused sodium acetate (2 mmol, 0.16 g) were fused until the reaction end up (TLC). The mix was chilled then the solid was recrystallized utilizing ethyl alcohol as a yellowish white powder: Yield 81%; m.p: 215-

217 °C; IR (KBr) cm^{-1} 3270, 2954, 1690, 1657, 1638, 1601; ^1H NMR (500 MHz, DMSO-d6)δ(ppm): 12.18 (s, 1H), 8.16 (s, 1H), 7.41-7.42 (d,1H, *J*= 2 Hz), 6.46-6.47 (m, 1H), 6.27-6.28 (d, 1H, *J*= 2.5 Hz), 5.17 (s, 1H), 2.14 (s, 3H); ^{13}C NMR (100 MHz, DMSO-d6)δ(ppm): 168, 163.99, 159.45, 157.56, 156.17, 153.25, 143.44, 137.39, 136.47, 112.73, 108.16, 100.89, 99.97, 31.59, 10.69; EIMS, m/z [M]+ calcd: 432.98; found: 432.99; Elemental Analysis for C17H9Cl2N5O3S (%), Calcd: C, 47.02; H, 2.09; N, 16.13; found: C, 46.95; H, 2.06; N, 16.08.

(E)-3-methyl-6-(((4-methyl-4H-1,2,4-triazol-3-yl)methylene)amino)-4-(thiophen-2-yl)-1,4-dihydropyrano[2,3-c]pyrazole-5-carbonitrile (4)

The ethanolic solution of pyranopyrazole derivative **A** (5 mmol, 1.29 g) and 4-methyl-4*H*-1,2,4-triazole-3-carbaldehyde (5 mmol, 0.56 g) was refluxed in existing of glacial acetic acid (5 drops, catalyst) for 6 h then iced. The precipitate was filtered and recrystallized utilizing ethyl alcohol as a white powder: Yield 90%; m.p: 243-245 °C; IR (KBr) cm^{-1} 3345, 3026, 2924, 2220, 1630, 1586; ^1H NMR (500 MHz, DMSO-d_6) δ (ppm): 12.22 (s, 1H), 8.58 (s, 1H), 8.24 (s, 1H), 7.48-7.49 (d,1H, *J*= 2 Hz), 6.32-6.33 (m, 1H), 6.126-6.131 (d, 1H, *J*= 2.5 Hz), 5.17 (s, 1H), 3.73 (s, 3H), 2.08 (s, 3H); ^{13}C NMR (100 MHz, DMSO-d_6) δ (ppm): 161.45, 160.99, 160.18, 152.67, 147.27, 142.99, 142.76, 136.34, 132, 119.12, 114.87, 110.09, 79.79, 33.75, 31.24, 10.95; EIMS, *m/z* $[M]^+$ calcd: 351.09; found: 351.07; Elemental Analysis for $C_{16}H_{13}N_7OS$ (%), Calcd: C, 54.69; H, 3.73; N, 27.9; found: C, 54.63; H, 3.69; N, 27.85.

3-methyl-6-(2-(4-methyl-4H-1,2,4-triazol-3-yl)-4-oxothiazolidin-3-yl)-4-(thiophen-2-yl)-1,4-dihydropyrano[2,3-c]pyrazole-5-carbonitrile (5)

A mix of pyrano[2,3-*c*]pyrazole congener **4** (2 mmol, 0.7g) and thioglycolic acid (2 mmol, 0.14 mL) in dry dioxane was refluxed until the reactants were consumed (detected by TLC), then chilled and treated with Na_2CO_3 solution. The formed solid was filtered off and recrystallized utilizing ethyl alcohol as a yellowish white powder: Yield 85%; m.p: 224-226 °C; IR (KBr) cm^{-1} 3384, 3034, 2984, 2938, 1730, 1707, 1604; ^1H NMR (500 MHz, DMSO-d_6) δ (ppm): 12.21 (s, 1H), 8.19 (s, 1H), 7.388-7.392 (d, 1H, *J*= 2 Hz), 6.29-6.3 (m, 1H), 6.105-6.11 (d, 1H, *J*= 2.5 Hz), 5.34 (s, 1H), 4.9 (s, 1H), 3.95 (s, 2H), 3.67 (s, 3H), 2.07 (s, 3H); ^{13}C NMR (100 MHz, DMSO-d_6)δ(ppm): 164.01, 159.45, 157.5, 156.16, 152.16, 142.98, 142.73, 136.18, 131.82, 119.01, 113.68, 109.17, 79.67, 59.34, 42.67, 33.64, 30.73, 10.84; EIMS, *m/z* $[M]^+$ calcd: 425.07; found: 425.09; Elemental Analysis for $C_{18}H_{15}N_7O_2S_2$ (%), Calcd: C, 50.81; H, 3.55; N, 23.04; found: C, 50.76; H, 3.51; N, 23.

*6-**(3-chloro-2-(4-methyl-4H-1,2,4-triazol-3-yl)-4-oxoazetidin-1-yl)-3-methyl-4-(thiophen-2-yl)-1,4-dihydropyrano[2,3-c]pyrazole-5-carbonitrile (6)***

A mixture of pyrano[2,3-*c*]pyrazole congener **4** (2 mmol, 0.7 g) and chloroacetyl chloride (2 mmol, 0.16 mL) was refluxed in DMF in attending of triethylamine (catalytic drops) until the reaction accomplishment (scrutinized by TLC). The mix was cooled, dispensed over icy water. The precipitate was filtered off and recrystallized exploiting ethyl alcohol as an off-white powder: Yield 83%; m.p: 210-212 °C; IR (KBr) cm^{-1} 3338, 3220, 3126, 3004, 2960, 2926, 2855,1744, 1658, 1600; ^1H NMR (500 MHz, DMSO-d_6)δ(ppm): 12.23 (s, 1H), 8.2 (s, 1H), 7.398-7.402 (d, 1H, *J*= 2 Hz), 6.31-6.32 (m, 1H), 6.118-6.123 (d, 1H, *J*= 2.5 Hz), 4.86 (s, 1H), 4.54-4.55 (d, 1H, *J*= 8.5 Hz, C_2H of Azetidine), 3.97-3.98 (d, 1H, *J*= 8.5 Hz, C_3H of Azetidine), 3.7 (s, 3H), 2.08 (s, 3H); ^{13}C NMR (100 MHz, DMSO-d_6)δ(ppm): 163.5, 158.91, 157, 155.64, 151.73, 142.48, 142.23, 135.68, 131.32, 118.51, 113.18, 108.67, 79.01, 61.45, 53, 33.46, 30.56, 10.66; EIMS, *m/z* $[M]^+$ calcd: 427.06; found: 427.05; Elemental Analysis for $C_{18}H_{14}ClN_7O_2S$ (%), Calcd: C, 50.53; H, 3.3; N, 22.92; found: C, 50.56; H, 3.28; N, 22.88.

Dimethyl(3-methyl-5-oxo-6-(1,2,3-thiadiazol-4-yl)-4-(thiophen-2-yl)-4,5,6,7-tetrahydro-1H-pyrrolo[3',2':5,6]pyrano[2,3-c]pyrazol-6-yl)phosphonate (7)

A mix of pyranopyrazole derivative **A** (1 mmol, 0.26 g), 1,2,3-thiadazole-4-carbaldehyde (1 mmol, 0.11 g), and trimethyl phosphite (1 mmol, 0.12 mL) were dissolved in acetonitrile (15 mL) under vigorous stirring at ambient

temperature in the incidence of lithium perchlorate (20% mol). The reaction advancement was judged via TLC till the reaction accomplishment, then the precipitate designed was isolated, speckled numerous times with water, desiccated, and recrystallized applying (acetonitrile/ water) mix as a yellow orange powder: Yield 83%; m.p: >300 °C; IR (KBr) cm^{-1} 3387, 3296, 2950, 2850, 1692, 1646, 1609, 1231, 1035; ^1H NMR (500 MHz, DMSO-d_6)δ(ppm): 12.17 (s, 1H), 11.13 (s, 1H), 8.15 (s, 1H), 7.387-7.391 (d, 1H, *J*= 2 Hz), 6.22-6.23 (m, 1H), 6.03-6.04 (d, 1H, *J*= 2.5 Hz), 5.18 (s, 1H), 3.83-3.85 (d, 3H, *J*= 8 Hz), 3.63-3.64 (d, 3H, *J*= 8 Hz), 2.05 (s, 3H); ^{31}P NMR (162 MHz, DMSO-d_6): 22 ppm; ^{13}C NMR (100 MHz, DMSO-d_6) δ (ppm): 164.55, 159.09, 157.16, 151.82, 144.26, 144.01, 137.49, 133.16, 112.67, 108.17, 100.72, 100, 69.5, 68, 54.15, 53.95, 31.63, 11.37; EIMS, *m/z* $[M]^+$ calcd: 465.03; found: 465.02; Elemental Analysis for $C_{17}H_{16}N_5O_5PS_2$ (%), Calcd: C, 43.87; H, 3.47; N, 15.05; found: C, 43.82; H, 3.41; N, 15.08.

1,9-dimethyl-4-(1,2,3-thiadiazol-4-yl)-10-(thiophen-2-yl)-3-(trifluoromethyl)-4,5,7,10-tetrahydropyrazolo[3,4-b]pyrazolo[4',3':5,6]pyrano[2,3-f][1,5]oxazocin-11(1H)-imine (8)

To a vigorously stirred solution of 1,2,3-thiadiazole-4-carbaldehyde (1 mmol, 0.11 g), pyranopyrazole derivative **A** (1 mmol, 0.26 g), and 1-methyl-3-(trifluoromethyl)-1H-pyrazole-5-ol (1 mmol, 0.17 g) in ethanol (15 mL), zinc chloride (10% mol) catalytic quantity was supplied. In an oil tub, the mix was refluxed for 6–8 h. Afterward the reaction accomplishment (detected by TLC), the solvent was detached under vacuum. In ethyl acetate, the residue was thawed, and brine was inserted. The aqueous layer was extracted with ethyl acetate (15 mL x 3) after separating the organic layer. The collective organic phase was splashed with water and dehydrated over anhydrous sodium sulphate subsequent with the solvent removal under vacuum. Reliant upon column chromatography, the residue was purified on silica gel [eluent: hexane and ethyl acetate (6:4)] providing a light yellow powder: Yield 75%; m.p: >300 °C; IR (KBr) cm^{-1} 3385, 3276, 3194, 3095, 2934, 1667, 1646, 1606, 1243; ^1H NMR (500 MHz, DMSO-d6)δ(ppm): 12.15 (s, 1H), 11.1 (s, br, 1H), 10.08 (s, 1H), 8.16 (s, 1H), 7.36-7.37 (d,1H, *J*=2 Hz), 6.19-6.2 (m, 1H), 6.005-6.01 (d,1H, *J*= 2.5 Hz), 5.14 (s, 1H), 4.74 (s, 1H), 3.66 (s, 3H), 2.03 (s, 3H); ^{13}C NMR (100 MHz, DMSO-d6)δ(ppm): 163.18, 158.54, 155.06, 153.68, 150.55, 142.25, 142, 135.41, 131.09, 126.83, 124.18, 113.67, 109.16, 102, 79.42, 49.34, 34.33, 27.46, 10.91; EIMS, m/z [M]+ calcd: 520.07; found: 521.02; Elemental Analysis for C20H15F3N8O2S2 (%), Calcd: C, 46.15; H, 2.9; N, 21.53; found: C, 46.07; H, 2.96; N, 21.49.

Ethyl (E)-N-(5-cyano-3-methyl-4-(thiophen-2-yl)-1,4-dihydropyrano[2,3-c]pyrazol-6-yl)formimidate (9)

A mixture of pyranopyrazole derivative A (1 mmol, 0.26 g), acetic anhydride (5 mL), and triethyl orthoformate (5 mL) was refluxed for 6 h. The mix was concentrated and the detached solid was filtered, dried, and recrystallized utilizing benzene as an off-white powder; Yield 86%; m.p: 195-197 °C; IR (KBr) cm^{-1} 3332, 3089, 2960, 2916, 2215, 1638, 1585, 1534, 1171; ^1H NMR (500 MHz, DMSO-d6)δ(ppm): 12.23 (s, 1H), 8.49 (s, 1H), 7.58-7.59 (d, 1H, J= 2 Hz), 6.41-6.42 (m, 1H), 6.226-6.231 (d, 1H, J= 2.5 Hz), 5.24 (s, 1H), 4.42-4.44 (q, 2H), 2.15 (s, 3H), 1.29-1.33 (t, 3H); ^{13}C NMR (100 MHz, DMSO-d6)δ(ppm): 157.63, 155.82, 154.37, 152.19, 142.37, 135.82, 120.91, 110.73, 106.18, 95.64, 63.63, 62.36, 30.17, 13.63, 10.36; EIMS, m/z [M]+ calcd: 314.08; found: 314.09; Elemental Analysis for C15H14N4O2S (%), Calcd: C, 57.31; H, 4.49; N, 17.82; found: C, 57.26; H, 4.43; N, 17.78.

5-imino-3-methyl-4-(thiophen-2-yl)-1,4-dihydropyrazolo[4',3':5,6]pyrano[2,3-d]pyrimidin-6(5H)-amine (10)

An ethanolic solution of compound **9** (5 mmol, 1.57 g) and hydrazine hydrate (5 mmol, 0.25 mL) was refluxed till the reactants consumed (detected by TLC). The mix was chilled and the precipitate was filtered off, dehydrated, and recrystallization from ethyl alcohol as a white powder: Yield 85%; m.p: 204-206 °C; IR (KBr) cm^{-1} 3398, 3237, 3129, 2924, 1648, 1605; ^1H NMR (500 MHz, DMSO-d_6) δ (ppm): 12.2 (s, 1H), 9.26 (s, 1H), 8.72 (s, 1H), 7.338-7.342 (d, 1H, *J*= 2 Hz), 6.43-6.44 (m, 1H), 6.23-6.24 (d, 1H, *J*= 2.5 Hz), 5.12 (s, 1H), 4.83 (s, 2H), 2.17 (s, 3H); ^{13}C NMR (100 MHz, DMSO-d_6)δ(ppm): 159.82, 157.5, 156.01, 152.73, 143.16, 142.17, 136.18, 112.55, 108, 100.73, 97.27, 27.09, 10.73; EIMS, *m/z* $[M]^+$ calcd: 300.08; found: 300.08; Elemental Analysis for $C_{13}H_{12}N_6OS$ (%), Calcd: C, 51.99; H, 4.03; N, 27.98; found: C, 51.94; H, 3.99; N, 27.93.

10-methyl-11-(thiophen-2-yl)-8,11-dihydropyrazolo[4',3':5,6]pyrano[3,2-e][1,2,4]triazolo[1,5-c]pyrimidine-2(3H)-thione (11)

A mixture of compound **10** (2 mmol, 0.6 g), carbon disulphide (4 mmol, 0.24 mL), and potassium hydroxide (4 mmol, 0.22 g) was refluxed in ethanol and the reaction advancement was scrutinized through TLC until all reactants' consumption. Afterward refrigeration, the mix was dispensed over HCl/ice and the detached precipitate was filtered and recrystallized utilizing water as a yellowish white powder; Yield 91%; m.p: 208-210 °C; IR (KBr) cm^{-1} 3321, 3245, 2954, 1646, 1610, 1453; ^{1}H NMR (500 MHz, DMSO-d_6)δ(ppm): 12.21 (s, 1H), 11.46 (s, br, 1H), 8.73 (s, 1H), 7.62-7.63 (d, 1H, *J*= 2 Hz, Ar-H), 6.46-6.47 (m, 1H, Ar-H), 6.266-6.271 (d, 1H, *J*= 2.5 Hz, Ar-H), 5.16 (s, 1H), 2.06 (s, 3H); ^{13}C NMR (100 MHz, DMSO-d_6)δ(ppm): 169.27, 160.52, 158.2, 156.71, 153.43, 143.86, 142.87, 136.88, 113.25, 108.7, 101.43, 97.97, 27.82, 11.43; EIMS, *m/z* $[M]^+$ calcd: 342.04; found: 342.03; Elemental Analysis for $C_{14}H_{10}N_6OS_2$ (%), Calcd: C, 49.11; H, 2.94; N, 24.55; found: C, 49.06; H, 2.89; N, 24.51.

10-methyl-11-(thiophen-2-yl)-8,11-dihydropyrazolo[4',3':5,6]pyrano[3,2-e][1,2,4]triazolo[1,5-c]pyrimidine (12)

A mix of pyrazolo[4',3':5,6]pyrano[2,3-*d*]pyrimidin-6(5*H*)-amine **10** (2 mmol, 0.6 g) and formic acid (10 mL) was refluxed till the reaction completion (TLC scrutinization). The mix was chilled, bestowed over ice-cold water and the precipitate was isolated, dried, and recrystallized operating methyl alcohol as an off-white powder: Yield 83%; m.p: 196-198 °C; IR (KBr) cm^{-1} 3256, 3065, 2942, 1652, 1639, 1609; ^{1}H NMR (500 MHz, DMSO-d_6)δ(ppm): 12.22 (s, 1H), 8.58 (s, 1H), 8.24 (s, 1H), 7.48-7.49 (d, 1H, *J*= 2 Hz), 6.32-6.33 (m, 1H), 6.126-6.131 (d, 1H, *J*= 2.5 Hz), 5.17 (s, 1H), 2.08 (s, 3H); ^{13}C NMR (100 MHz, DMSO-d_6)δ(ppm): 159.47, 157.15, 155.66, 152.38, 142.81, 141.82, 135.84, 132.66, 112.2, 107.65, 100.38, 96.93, 26.74, 10.38; EIMS, *m/z* $[M]^+$ calcd: 310.06; found: 310.06; Elemental Analysis for $C_{14}H_{10}N_6OS$ (%), Calcd: C, 54.18; H, 3.25; N, 27.08; found: C, 54.14; H, 3.21; N, 27.03.

10-methyl-11-(thiophen-2-yl)-2-(1H-1,2,4-triazol-3-yl)-8,11-dihydropyrazolo[4',3':5,6]pyrano[3,2-e][1,2,4]triazolo[1,5-c]pyrimidine (13)

A mix of compound **10** (1 mmol, 0.3 g) and *1H*-1,2,4-triazole-3-carbohydrazide (1.2 mmol, 0.15 g) in ethanol (15 mL) was refluxed for 6-7 h then chilled. The attained solid was filtered off, desiccated, and recrystallized utilizing methyl alcohol as a white powder: Yield 83%; m.p: 209-211 °C; IR (KBr) cm^{-1} 3316, 3265, 3043, 2955, 1664, 1643, 1607; ^{1}H NMR (500 MHz, DMSO-d_6)δ(ppm): 12.93 (s, 1H), 12.23 (s, 1H), 8.59 (s, 1H), 8.25 (s, 1H), 7.495-7.499 (d, 1H, *J*= 2 Hz), 6.33-6.34 (m, 1H), 6.138-6.143 (d, 1H, *J*= 2.5 Hz), 5.18 (s, 1H), 2.09 (s, 3H); ^{13}C NMR (100 MHz, DMSO-d_6)δ(ppm): 159.65, 157.33, 155.84, 152.56, 142.99, 142, 136.01, 132.84, 112.37, 107.83, 100.56, 97.1, 26.92, 10.56; EIMS, *m/z* $[M]^+$ calcd: 377.08; found: 377.06; Elemental Analysis for $C_{16}H_{11}N_9OS$ (%), Calcd: C, 50.92; H, 2.94; N, 33.4; found: C, 50.86; H, 2.98; N, 33.47.

General procedure for synthesizing pyranopyrazole derivatives (14-18)

An equimolar amount of pyranopyrazole derivative **A** (2 mmol, 0.52 g) and different sugars derivatives, namely, D-xylose **14** (2 mmol, 0.3 g), D-arabinose **15** (2 mmol, 0.3 g), D-ribose **16** (2 mmol, 0.3 g), D-mannose **17** (2 mmol, 0.36 g), D-galactose **18** (2 mmol, 0.36 g), respectively, were refluxed in ethyl alcohol in existing of glacial acetic acid (a catalytic volume) till the reaction completion (scrutinized *via* TLC). The reaction mix was chilled then dispensed over icy water. The molded solid was filtered off and recrystallized applying ethyl alcohol.

3- methyl-6-(((2S,3R,4R,E)-2,3,4,5-tetrahydroxypentylidene)amino)-4-(thiophen-2-yl)-1,4-dihydropyrano[2,3-c]pyrazole-5-carbonitrile (14)

White powder; Yield 78%; m.p: >300 °C; IR (KBr) cm^{-1} 3352, 3224, 2981, 2927, 2870, 2207, 1618; ^{1}H NMR (500 MHz, DMSO-d_6) δ (ppm): 12.13 (s, 1H), 8.20-8.21 (d, 1H, *J*= 8 Hz), 7.659-7.663 (d, 1H, *J*= 2 Hz), 6.54-6.55 (m, 1H), 6.177-6.182 (d, 1H, *J*= 2.5 Hz), 5.3-5.31 (m, 2H), 4.95-5.96 (m, 2H), 4.69 (s, 1H), 4.36-4.38 (m, 1H), 3.75-3.76 (m, 2H), 3.59-3.60 (m, 2H), 1.98 (s, 3H); ^{13}C NMR (100 MHz, DMSO-d_6) δ (ppm): 161.64, 156, 154.92, 150.91, 142.51, 136.01, 120.91, 110.37, 105.82, 95.28, 73.84, 72.34, 70.19, 64.19, 58.33, 30.18, 9.67; EIMS, *m/z* $[M]^+$ calcd: 390.1; found: 390.12; Elemental Analysis for $C_{17}H_{18}N_4O_5S$ (%), Calcd: C, 52.3; H, 4.65; N, 14.35; found: C, 52.35; H, 4.68; N, 14.32.

3-methyl-6-(((2R,3S,4R,E)-2,3,4,5-tetrahydroxypentylidene)amino)-4-(thiophen-2-yl)-1,4-dihydropyrano[2,3-c]pyrazole-5-carbonitrile (15)

Brownish white powder; Yield 81%; m.p: >300 °C; IR (KBr) cm^{-1} 3354, 3167, 2922, 2866, 2208,1618; ^{1}H NMR (500 MHz, DMSO-d_6) δ (ppm): 12.11 (s, 1H), 8.22-8.24 (d, 1H, *J*= 8.5 Hz), 7.58-7.59 (d, 1H, *J*= 2 Hz), 6.553-6.547 (m, 1H), 6.188-6.193 (d, 1H, *J*= 2.5 Hz), 5.40-5.41 (d, 1H, *J*= 6.5 Hz), 5-5.01 (m, 2H), 4.74 (s, 1H), 4.42-4.44 (t, 1H, *J*= 7 Hz), 3.89-3.9 (m, 1H), 3.71-3.73 (m, 2H), 3.59-3.60 (m, 2H), 1.91 (s, 3H); ^{13}C NMR (100 MHz, DMSO-d_6) δ (ppm): 161.64, 156, 154.92, 150.91, 142.51, 136.01, 120.91, 110.37, 105.82, 95.28, 73.84, 72.34, 70.19, 64.19, 58.33, 30.18, 9.67; EIMS, *m/z* $[M]^+$ calcd: 390.1; found: 390.09; Elemental Analysis for $C_{17}H_{18}N_4O_5S$ (%), Calcd: C, 52.3; H, 4.65; N, 14.35; found: C, 52.26; H, 4.6; N, 14.38.

3-methyl-6-(((2S,3R,4R,5R,E)-2,3,4,5,6-pentahydroxyhexylidene)amino)-4-(thiophen-2-yl)-1,4-dihydropyrano[2,3-c]pyrazole-5-carbonitrile (16)

White powder; Yield 77%; m.p: >300 °C; IR (KBr) cm^{-1} 3421, 3224, 2965, 2926, 2206,1619, 1561; ^{1}H NMR (500 MHz, DMSO-d_6) δ (ppm): 12.13 (s, 1H), 8.23-8.25 (d, 1H, *J*= 8 Hz), 7.59-7.60 (d, 1H, *J*= 2 Hz), 6.54-6.55 (m, 1H), 6.176-6.181 (d, 1H, *J*= 2.5 Hz), 5.39-5.4 (m, 1H), 4.99-5 (m, 2H), 4.74 (s, 1H), 4.42-4.44 (d, 1H, *J*= 7.5 Hz), 3.90-3.91 (m, 2H), 3.76-3.78 (t, 1H, *J*= 6.5 Hz), 3.56-3.58 (m, 2H), 1.92 (s, 3H); ^{13}C NMR (100 MHz, DMSO-d_6) δ (ppm): 161.638, 155.99, 154.91, 150.91, 142.5, 136, 120.91, 110.36, 105.82, 95.27, 73.84, 72.34, 70.19, 64.19, 63.67, 58.33, 30.18, 9.67; EIMS, *m/z* $[M]^+$ calcd: 420.11; found: 420.08; Elemental Analysis for $C_{18}H_{20}N_4O_6S$ (%), Calcd: C, 51.42; H, 4.79; N, 13.33; found: C, 51.38; H, 4.75; N, 13.36.

3-methyl-6-(((2R,3R,4R,5R,E)-2,3,4,5,6-pentahydroxyhexylidene)amino)-4-(thiophen-2-yl)-1,4-dihydropyrano[2,3-c]pyrazole-5-carbonitrile (17)

White powder; Yield 78%; m.p: >300 °C; IR (KBr) cm^{-1} 3385, 3184, 2983, 2930, 2208,1619; ^{1}H NMR (500 MHz, DMSO-d_6) δ(ppm): 12.15 (s, 1H), 8.17-8.18 (d, 1H, *J*= 6.5 Hz), 7.687-7.691 (d, 1H, *J*= 2 Hz), 6.63-6.64 (m, 1H), 6.27-6.28 (d, 1H, *J*= 2.5 Hz), 5.50-5.51 (d, 1H, *J*= 7 Hz), 5.11-5.13 (m, 2H), 4.82 (s, 1H), 4.36-4.39 (m, 3H), 3.74-3.75 (m, 2H), 3.56-3.58 (t, 1H, *J*= 7 Hz), 3.38-3.39 (m, 2H), 1.99 (s, 3H); ^{13}C NMR (100 MHz, DMSO-d_6) δ(ppm): 161.82, 156.17, 155.09, 151.10, 142.83, 136.34, 121.09, 110.55, 106.01, 95.46, 73.68, 71.68, 71.46, 69.84, 66.18, 63.67, 58.50, 30.36, 10.01; EIMS, *m/z* $[M]^+$ calcd: 420.11; found: 420.12; Elemental Analysis for $C_{18}H_{20}N_4O_6S$ (%), Calcd: C, 51.42; H, 4.79; N, 13.33; found: C, 51.36; H, 4.83; N, 13.28.

3-methyl-6-(((2S,3R,4S,5R,E)-2,3,4,5,6-pentahydroxyhexylidene)amino)-4-(thiophen-2-yl)-1,4-dihydropyrano[2,3-c]pyrazole-5-carbonitrile (18)

White powder; Yield 80%; m.p: >300 °C; IR (KBr) cm^{-1} 3354, 3237, 2983, 2929, 2208,1619; ^{1}H NMR (500 MHz, DMSO-d_6) δ (ppm): 12.14 (s, 1H), 8.26-8.28 (d, 1H, *J*= 7 Hz), 7.776-7.780 (d, 1H, *J*= 2 Hz), 6.768-6.774 (m, 1H), 6.41-6.42 (d, 1H, *J*= 2.5 Hz), 5.64-5.65 (d, 1H, *J*= 7 Hz), 5.25-5.26 (m, 2H), 4.98 (s, 1H), 4.42-4.44 (m, 2H), 3.67-3.69 (d, 1H, *J*= 7 Hz), 3.61-3.62 (m, 2H), 3.44-3.45 (t, 1H, *J*= 7 Hz), 3.30-3.31 (m, 2H), 1.99 (s, 3H); ^{13}C NMR (100 MHz, DMSO-d_6) δ (ppm): 162.17, 156.50, 155.46, 151.46, 143, 136.50, 121.27, 110.73, 106.37, 95.64, 73.83, 71.61, 70,

66.36, 63.84, 58.67, 30.55, 10.18; EIMS, *m/z* $[M]^+$ calcd: 420.11; found: 420.09; Elemental Analysis for $C_{18}H_{20}N_4O_6S$ (%), Calcd: C, 51.42; H, 4.79; N, 13.33; found: C, 51.37; H, 4.72; N, 13.29.

IR, ^{1}H NMR, and ^{13}C NMR spectra of the synthesized compounds

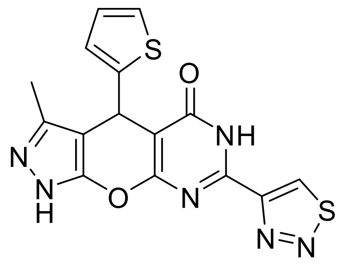

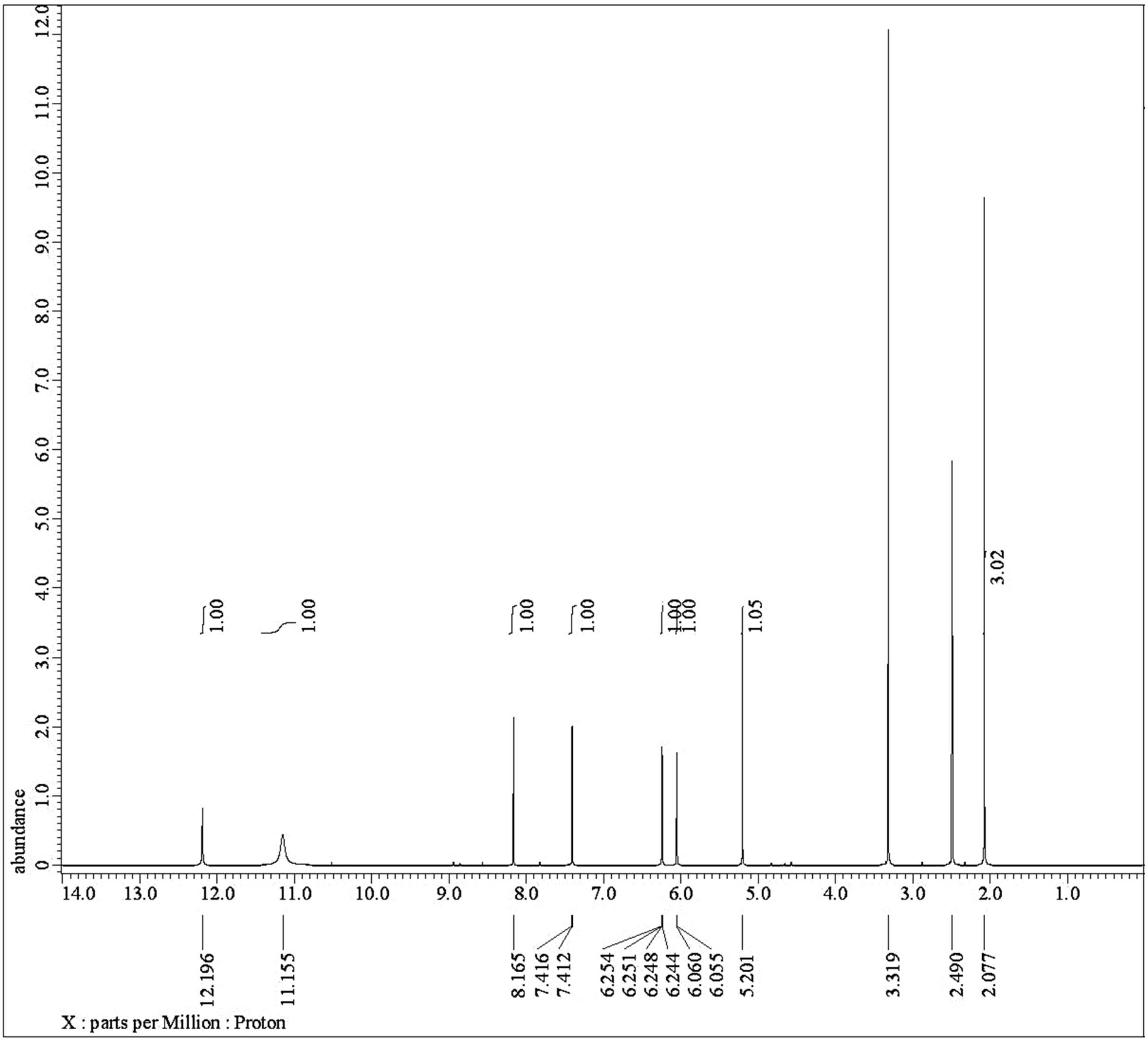

^{1}H NMR of Compound **1**

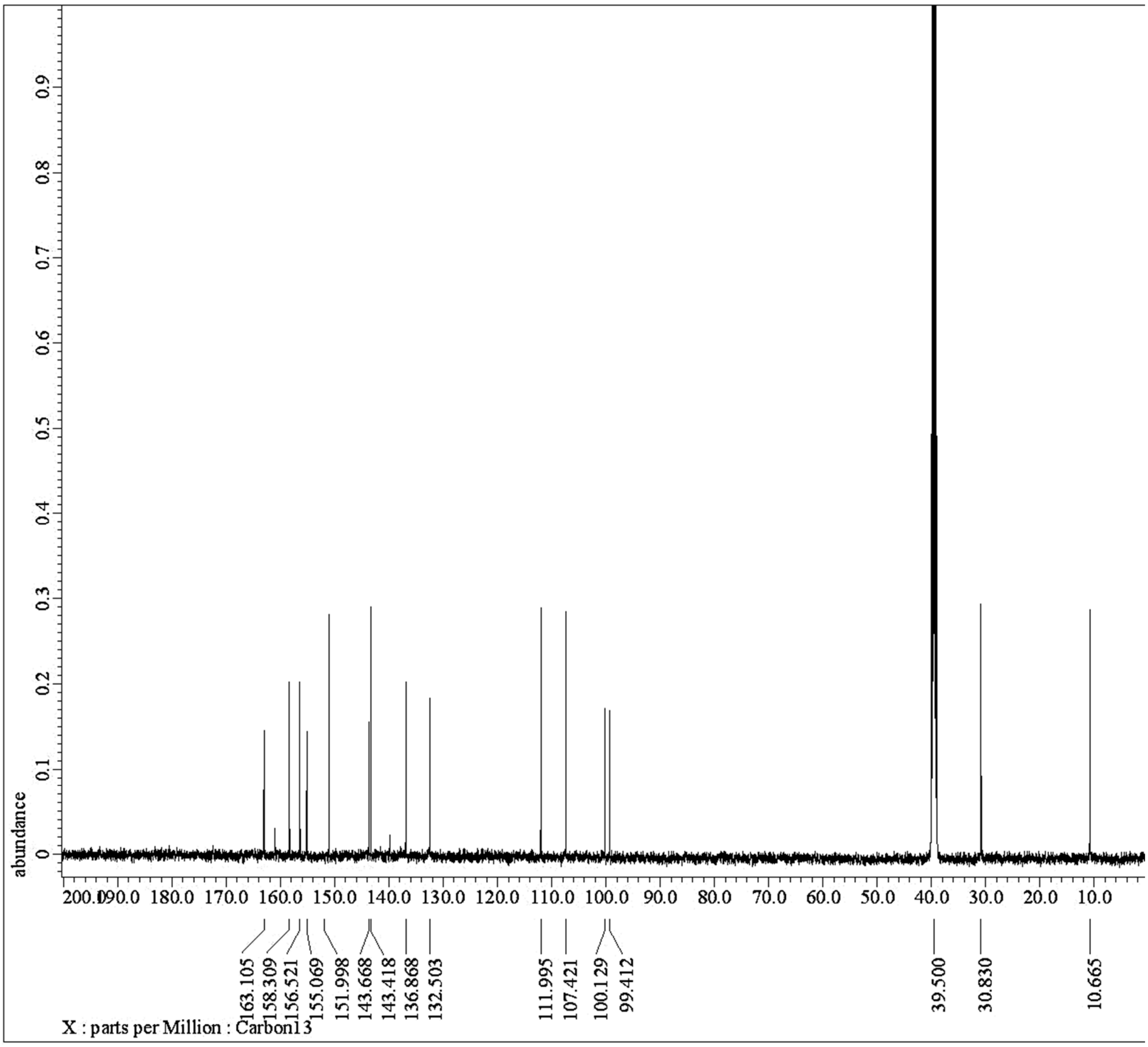

^{13}C NMR of Compound **1**

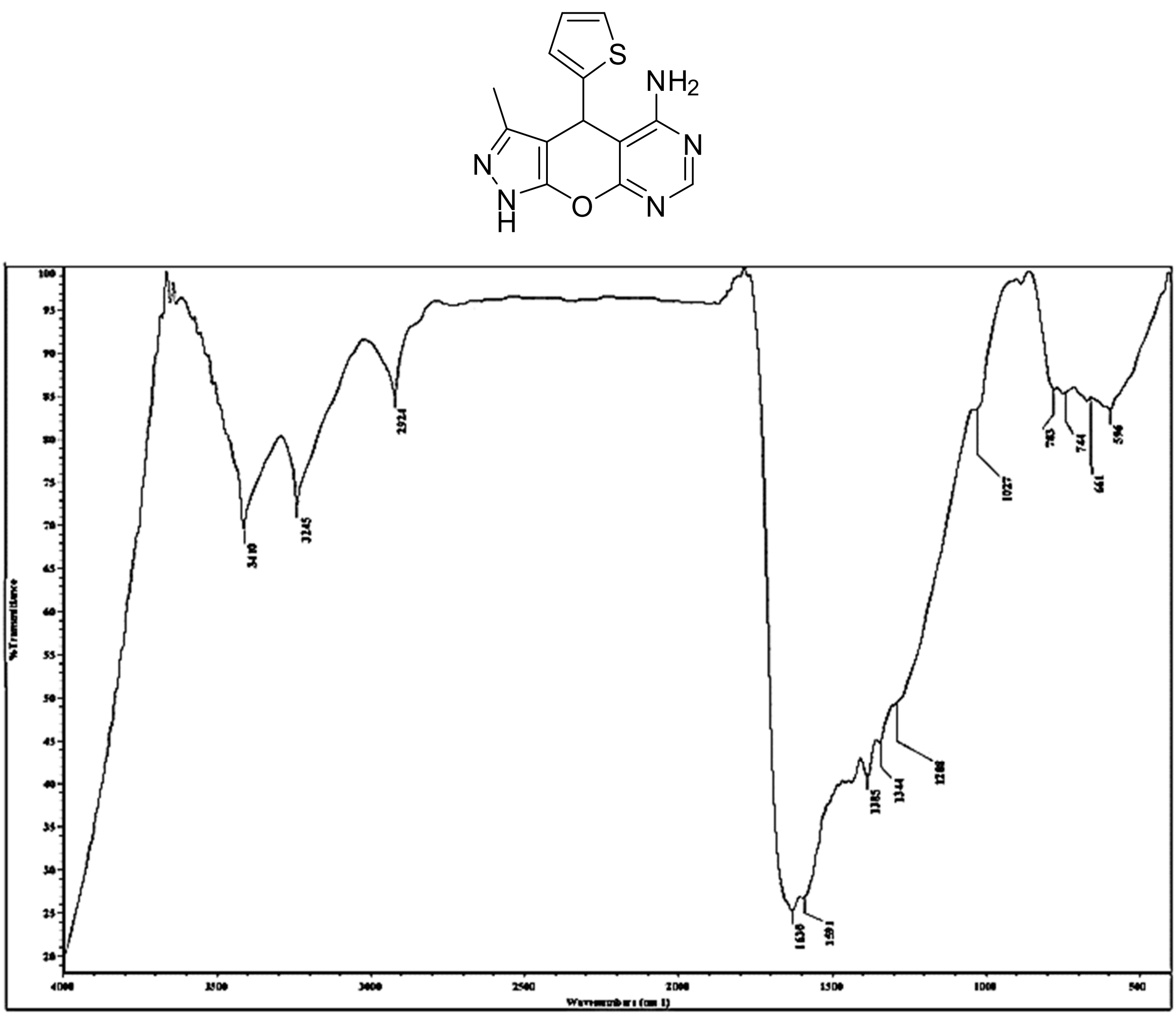

FT-IR of Compound **2**

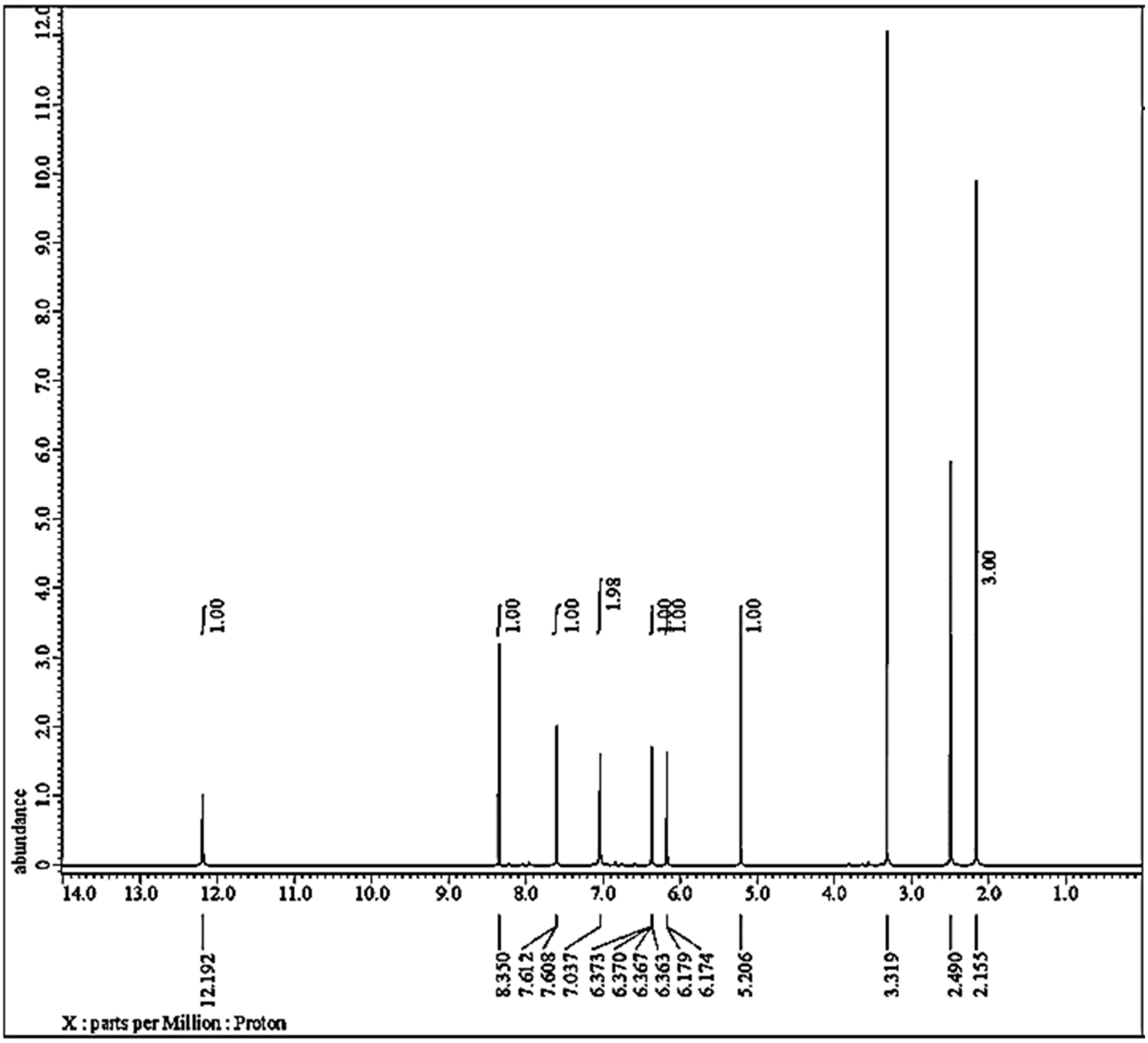

^{1}H NMR of Compound **2**

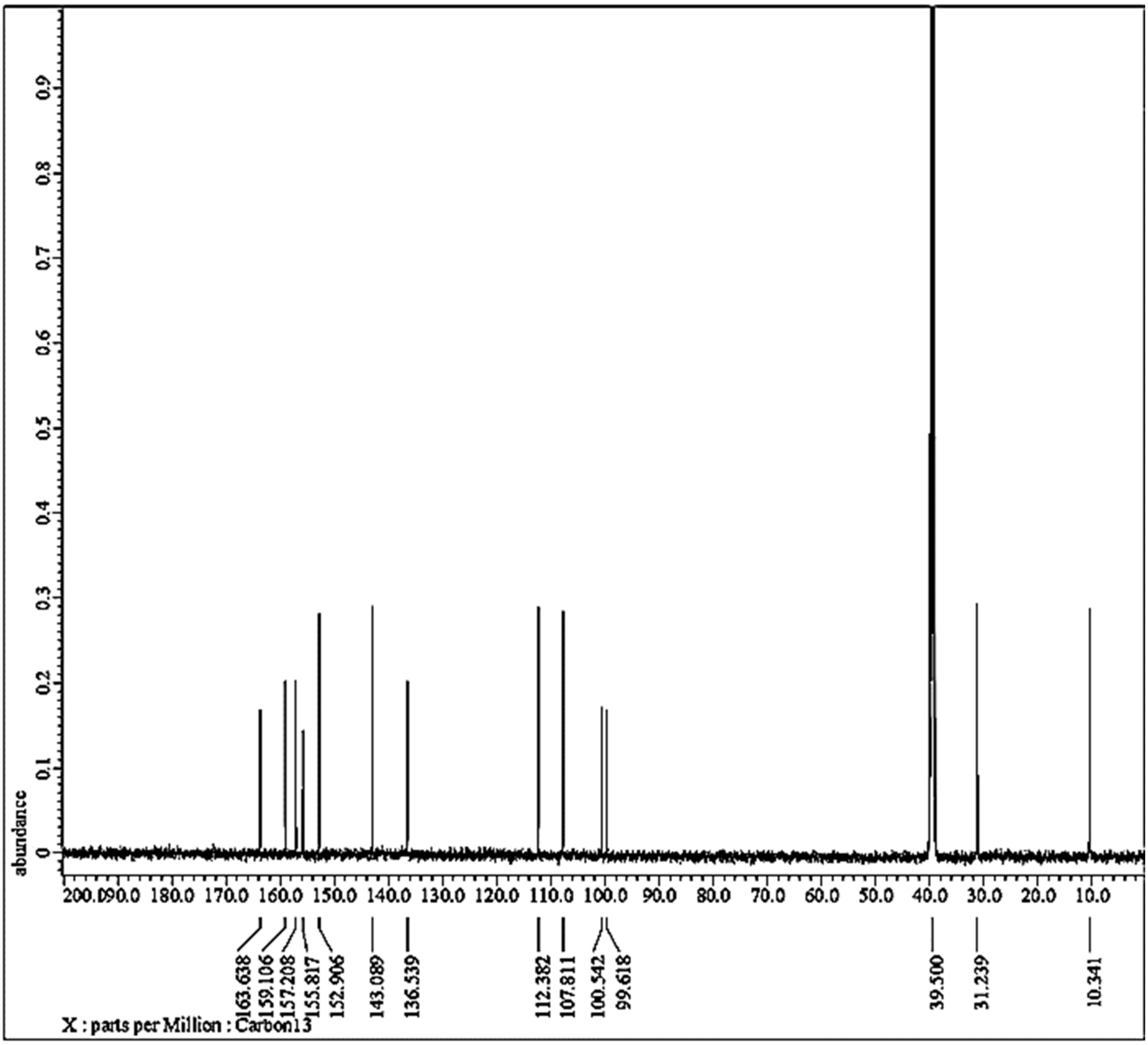

^{13}C NMR of Compound **2**

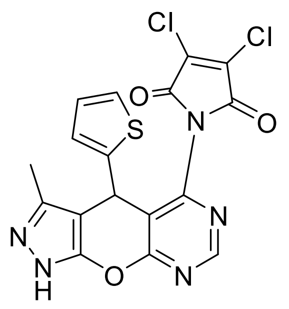

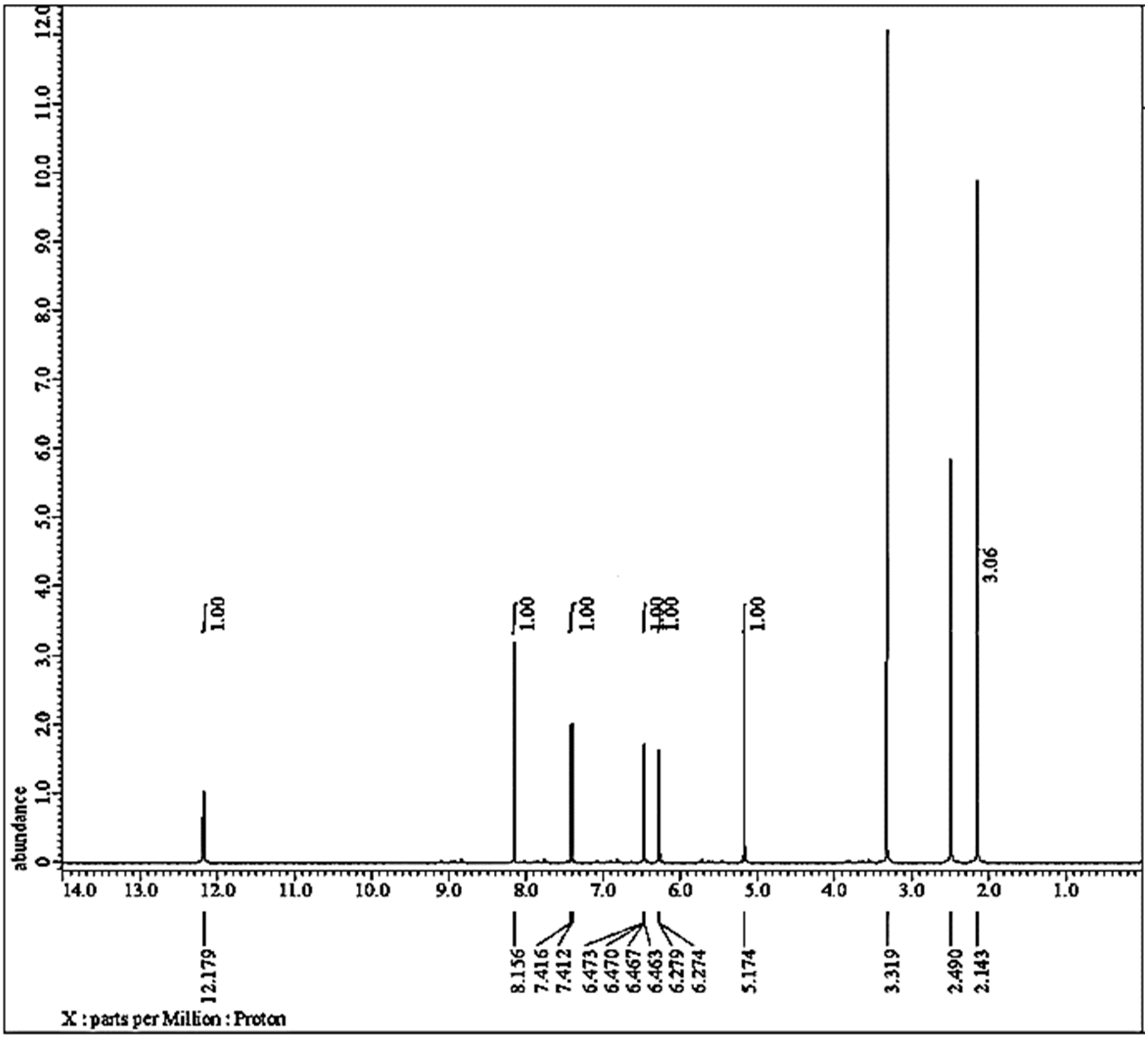

^{1}H NMR of Compound **3**

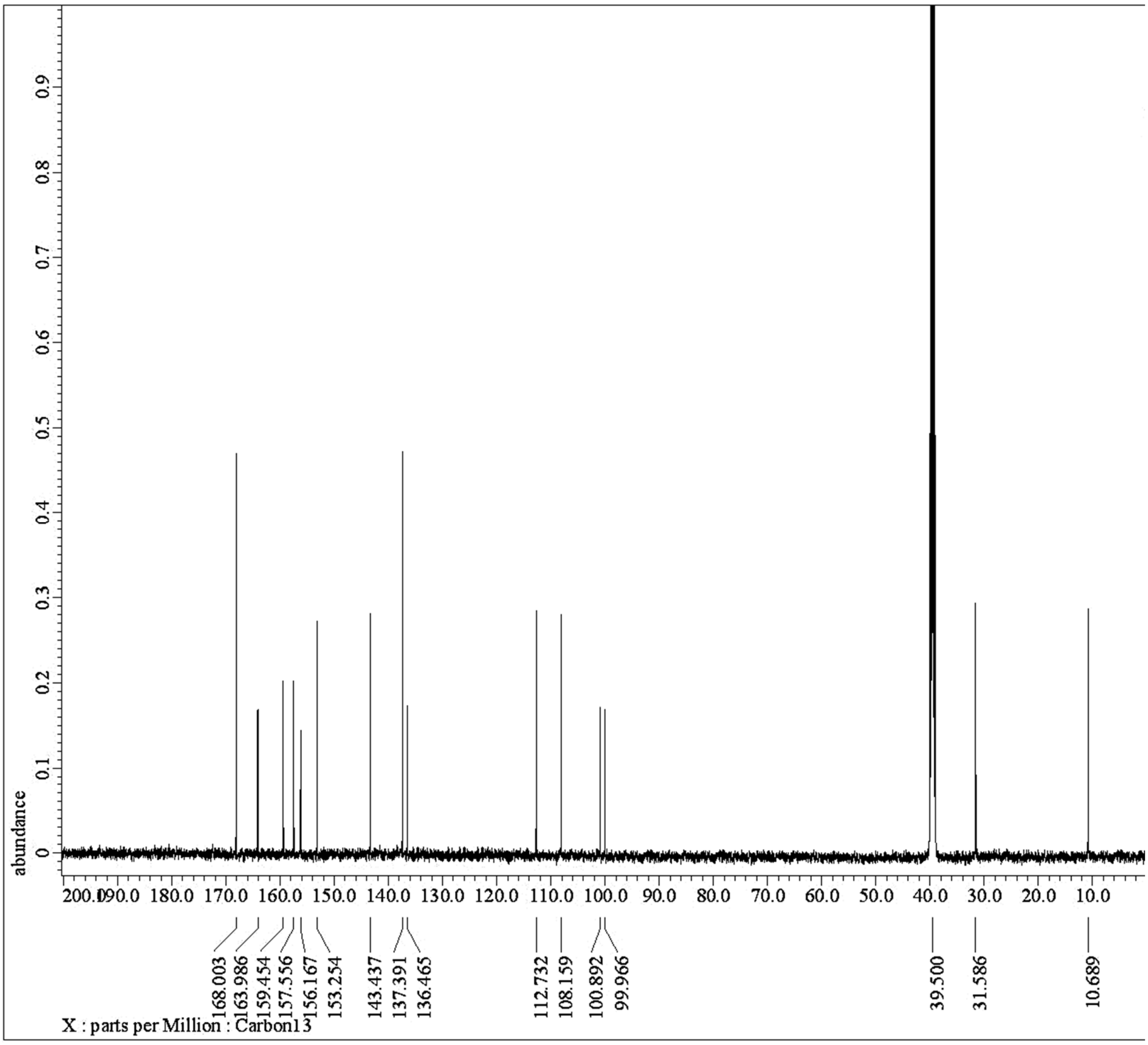

^{13}C NMR of Compound **3**

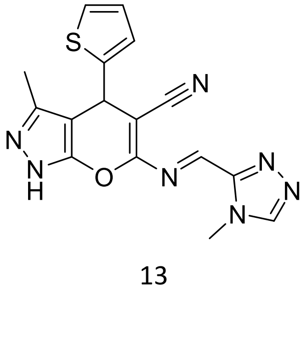

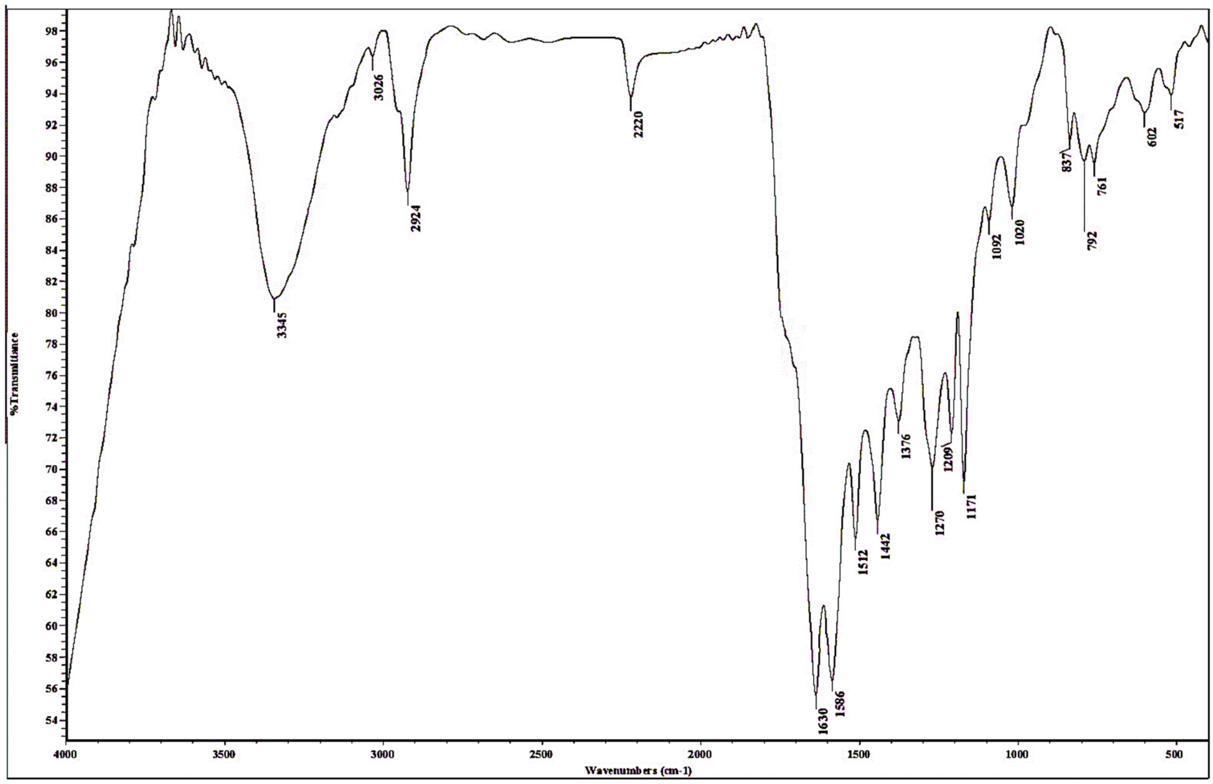

FT-IR of Compound **4**

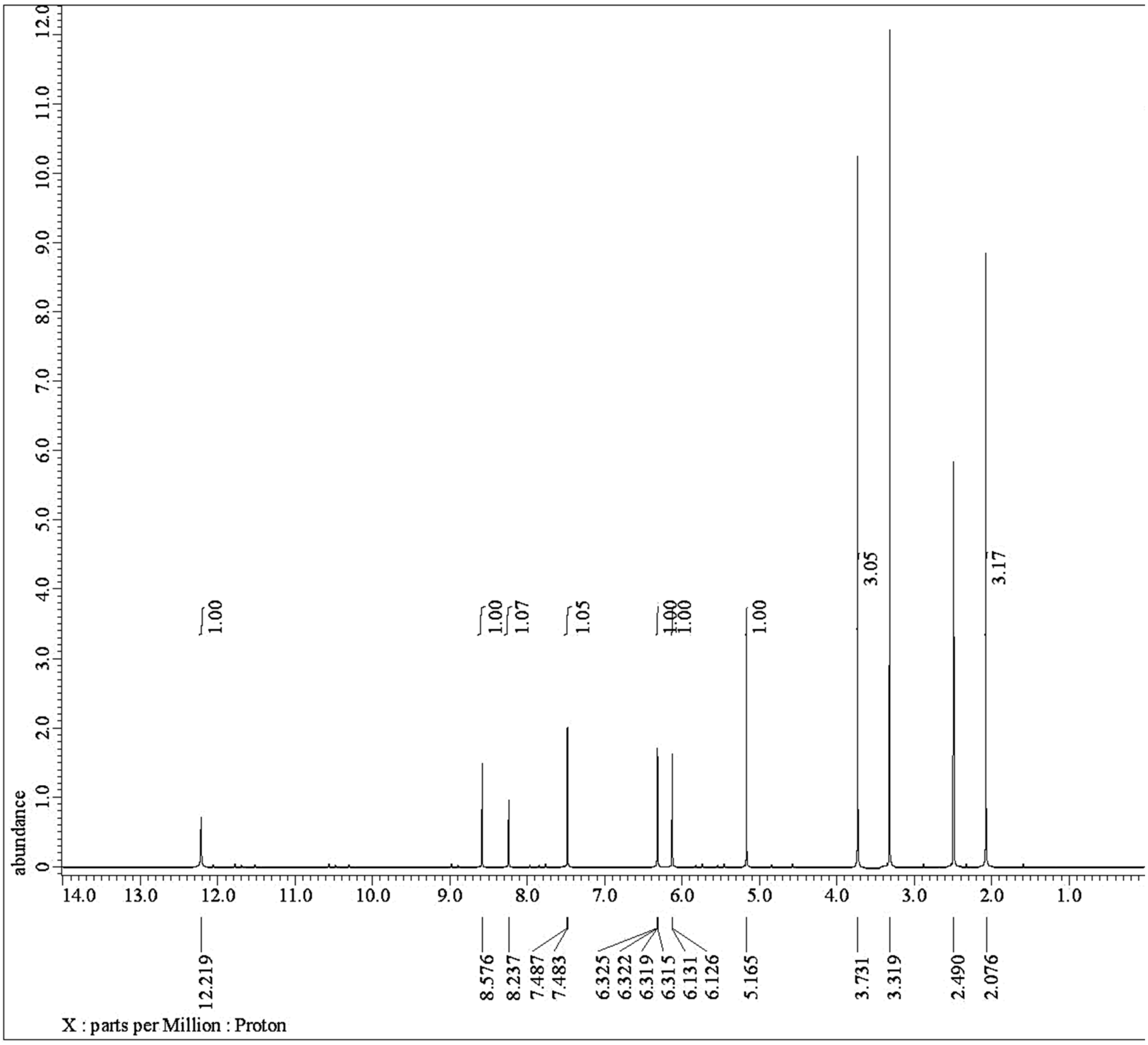

^{1}H NMR of Compound **4**

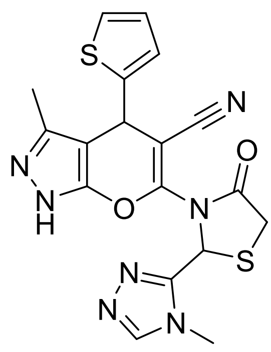

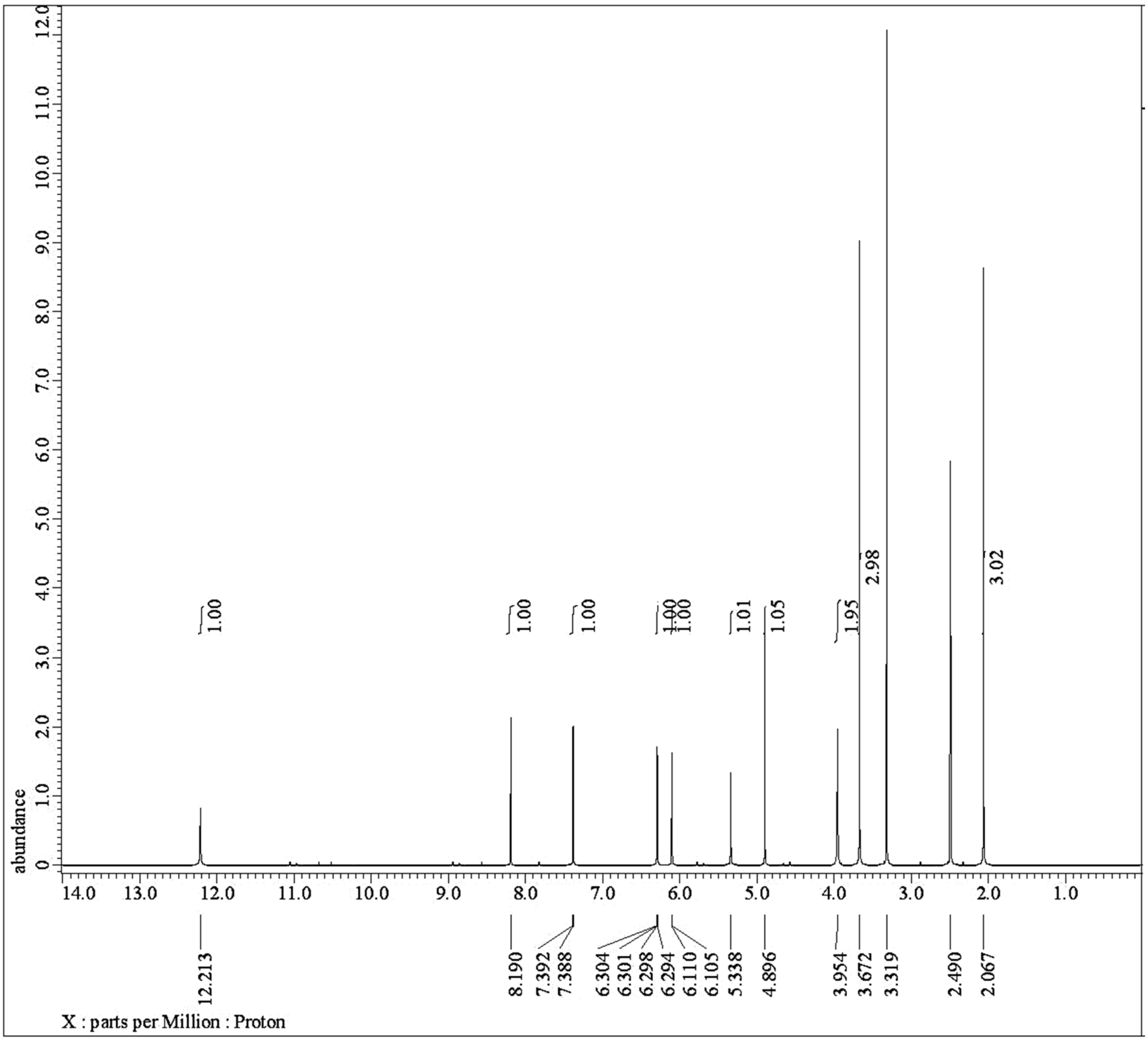

^{1}H NMR of Compound **5**

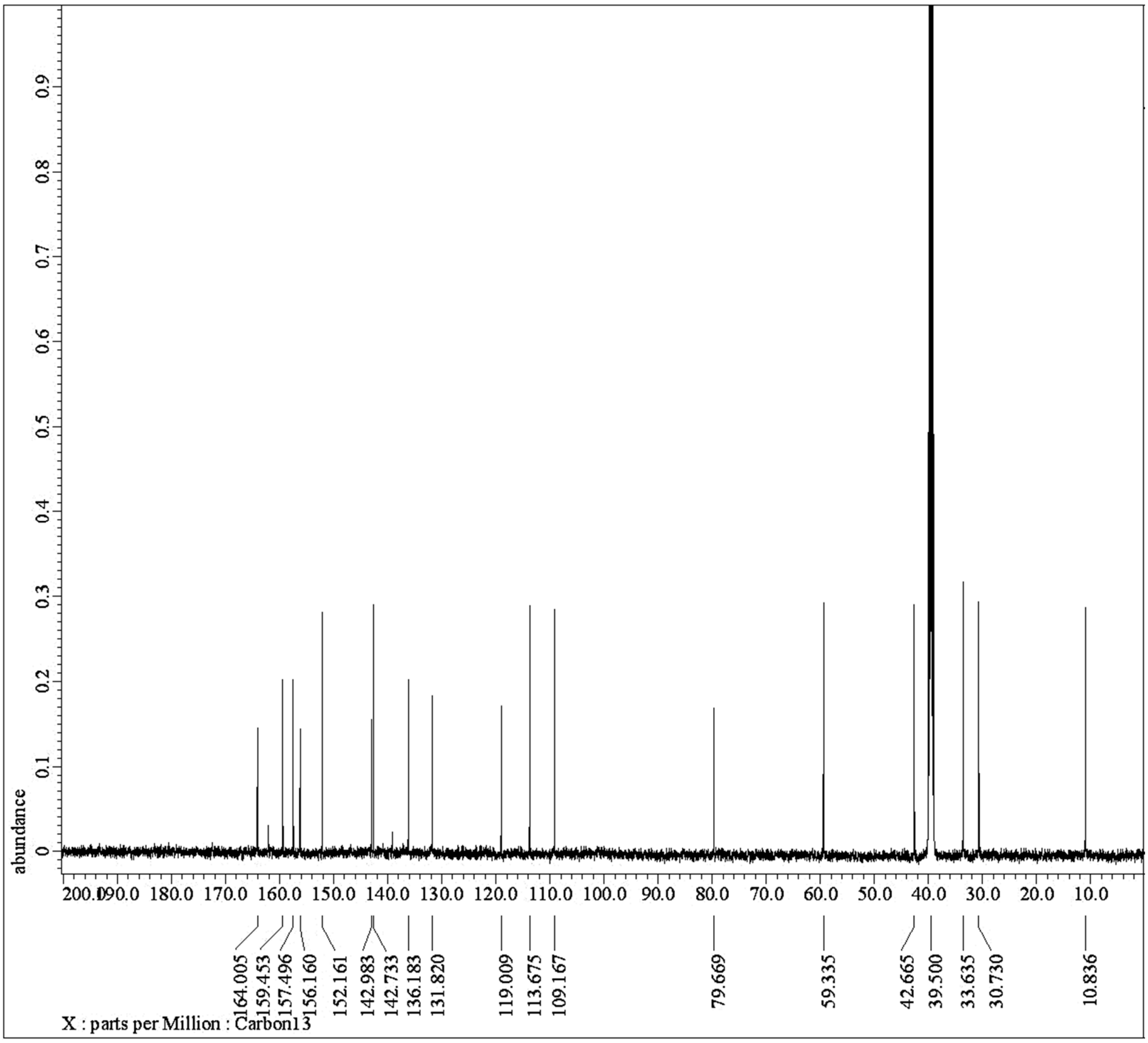

^{13}C NMR of Compound **5**

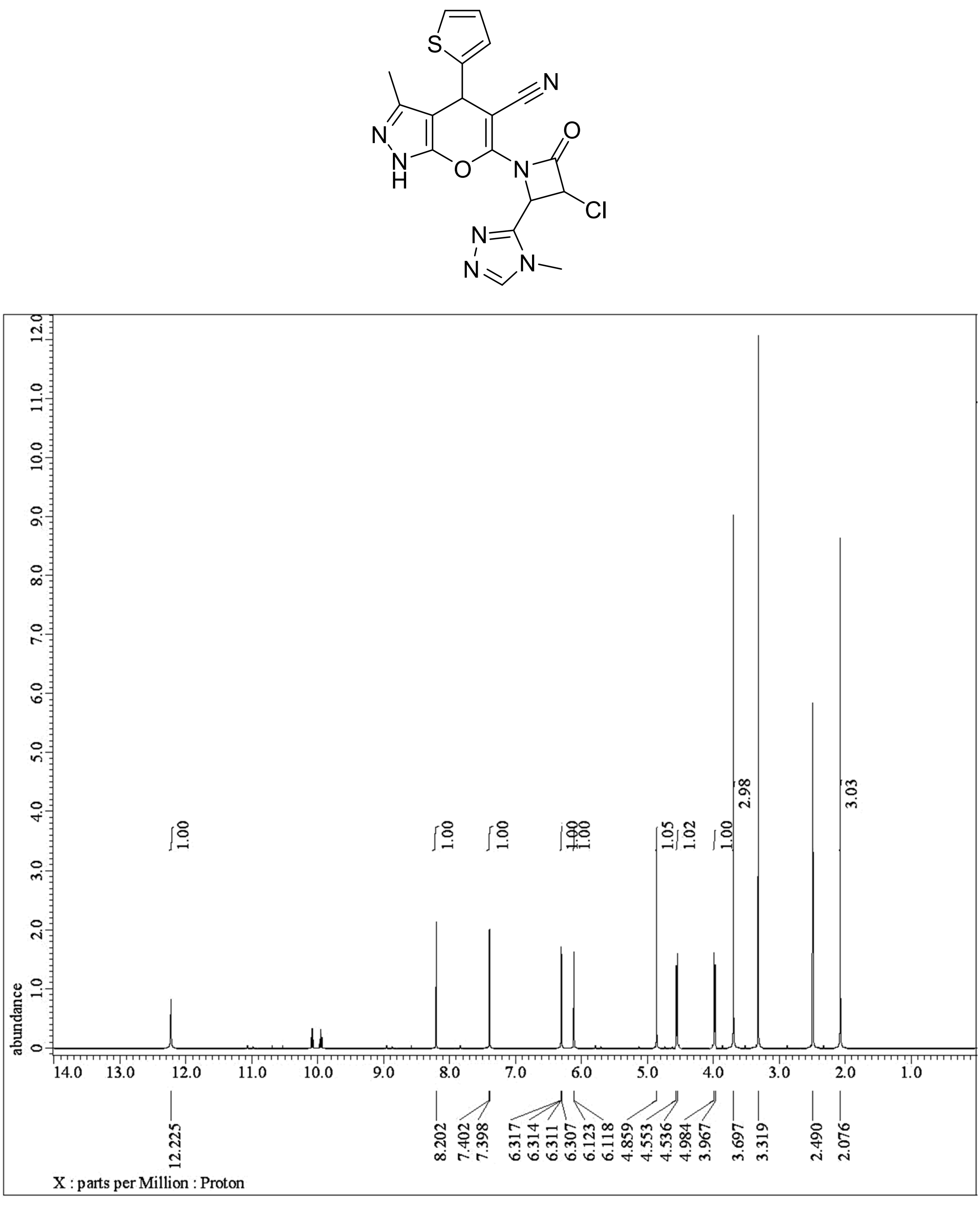

^{1}H NMR of Compound **6**

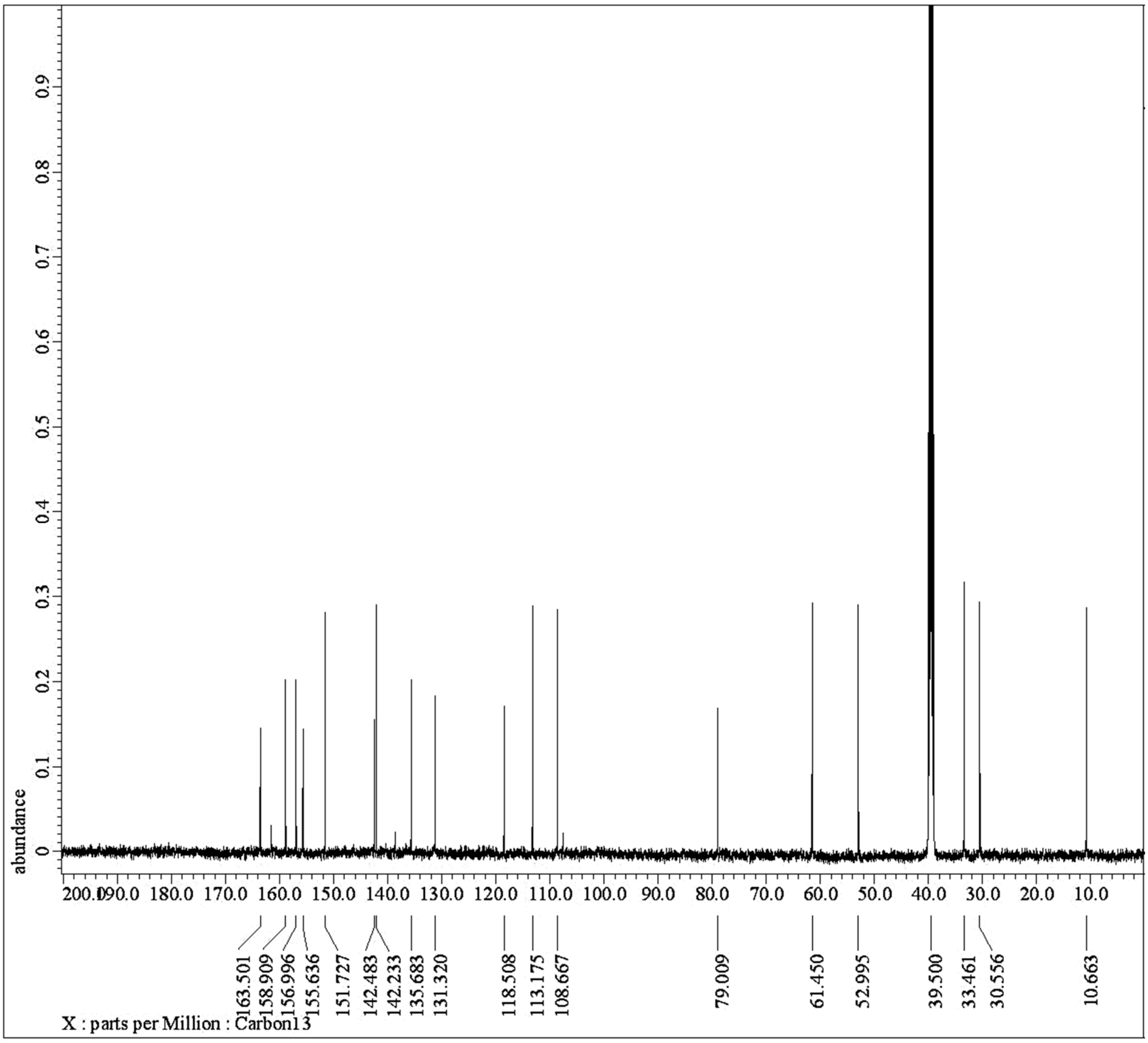

^{13}C NMR of Compound **6**

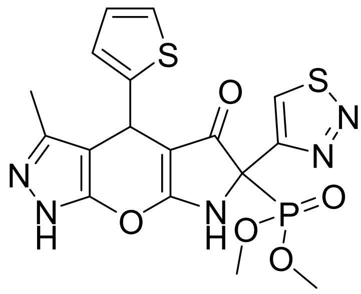

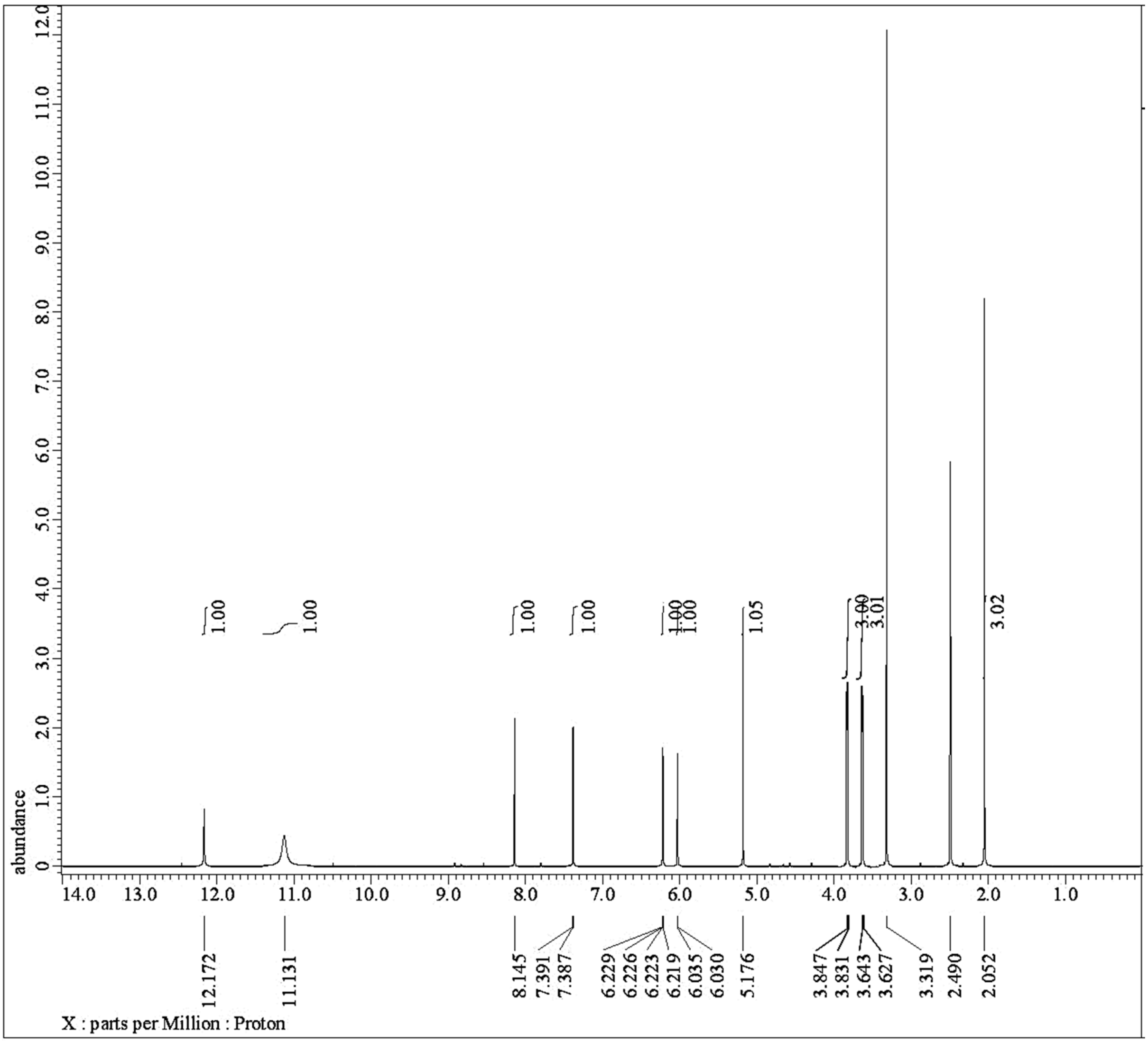

^{1}H NMR of Compound **7**

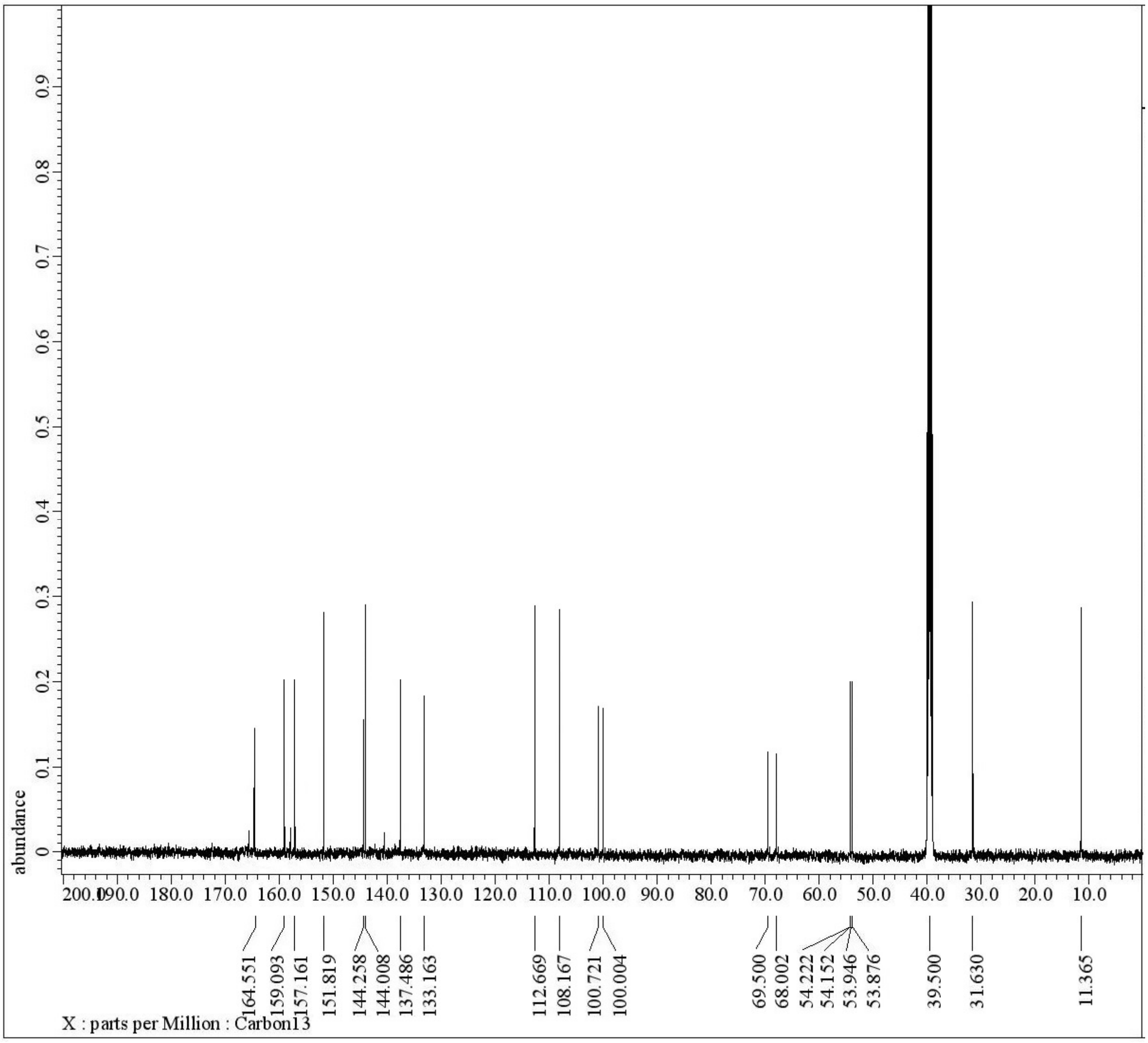

^{13}C NMR of Compound **7**

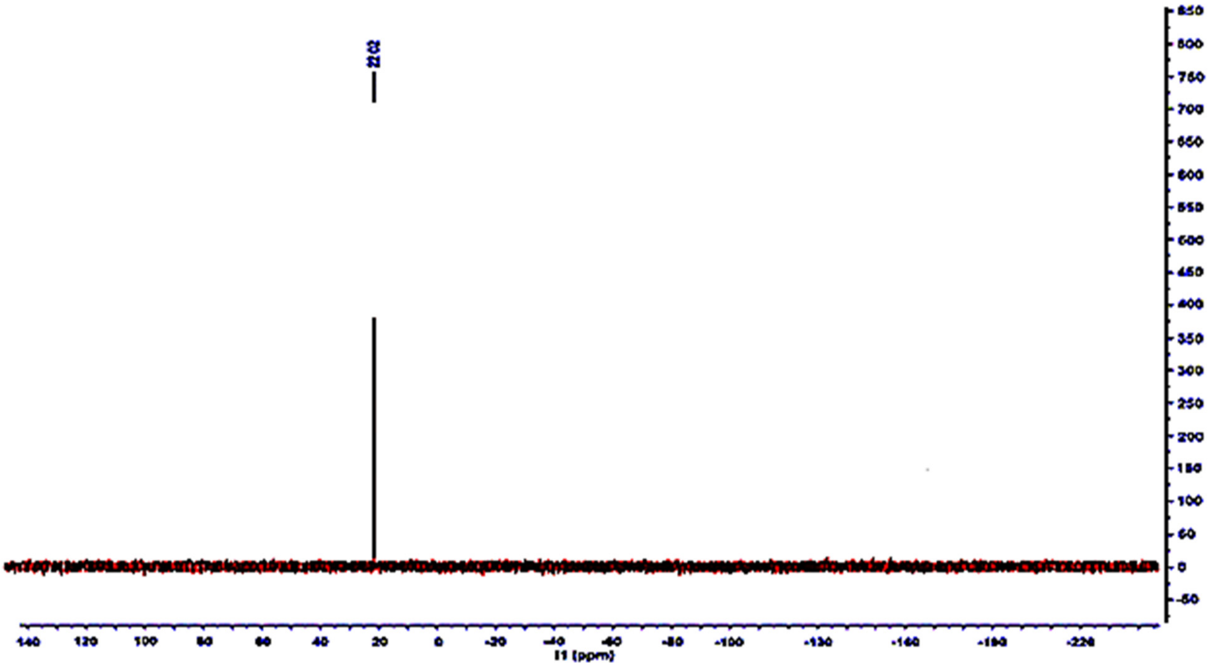

^{31}P NMR of Compound **7**

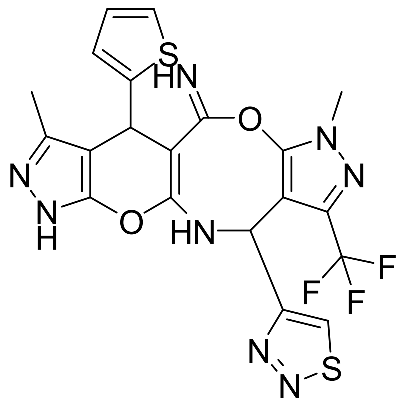

S
HN
O
N
N
N
N
H
O
HN
F
F
F
N
N
S

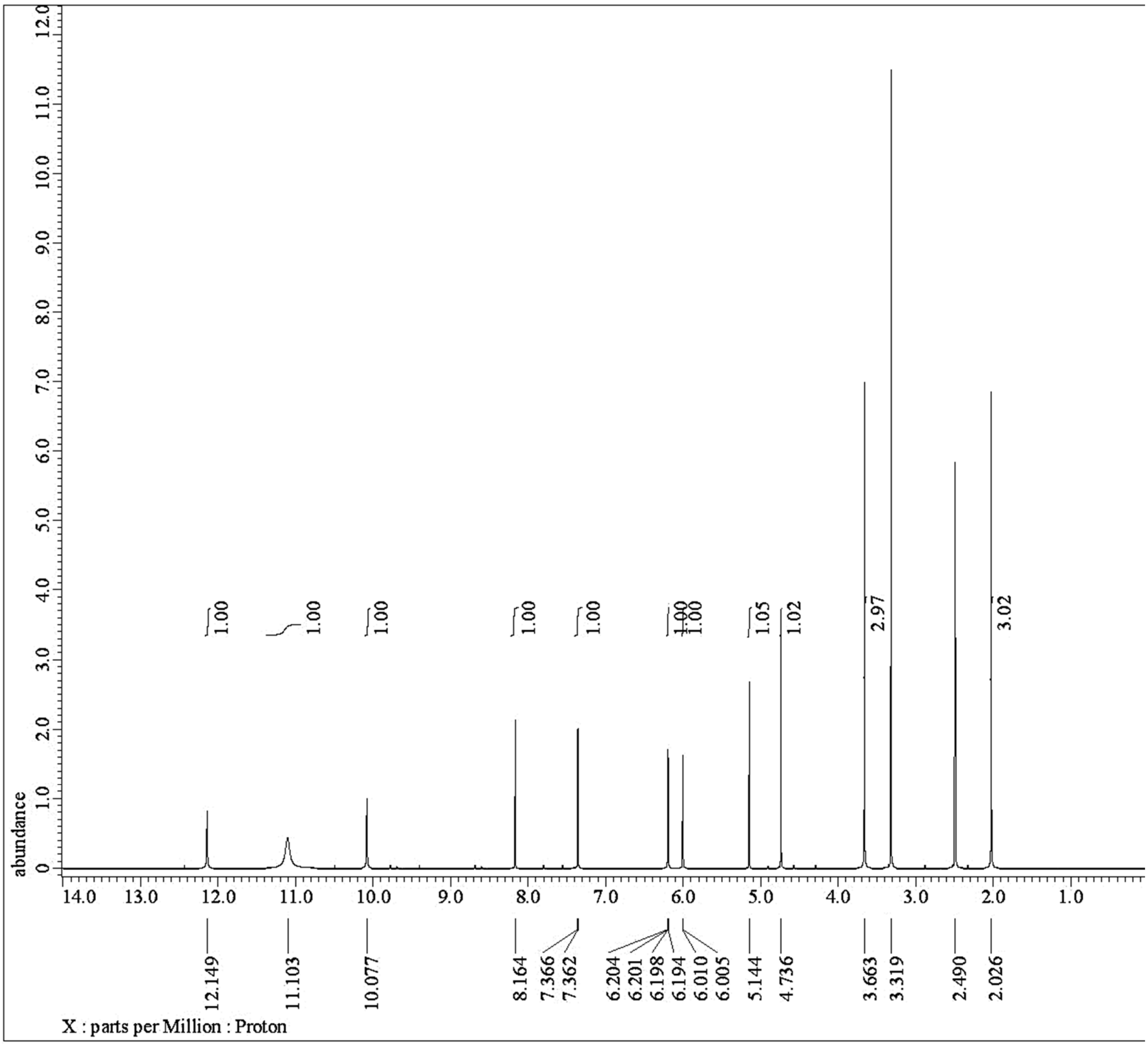

abundance
X : parts per Million : Proton
12.149
11.103
10.077
8.164
7.366
7.362
6.204
6.201
6.198
6.194
6.010
6.005
5.144
4.736
3.663
3.319
2.490
2.026
1.00
1.00
1.00
1.00
1.00
1.00
1.00
1.05
1.02
2.97
3.02

^{1}H NMR of Compound **8**

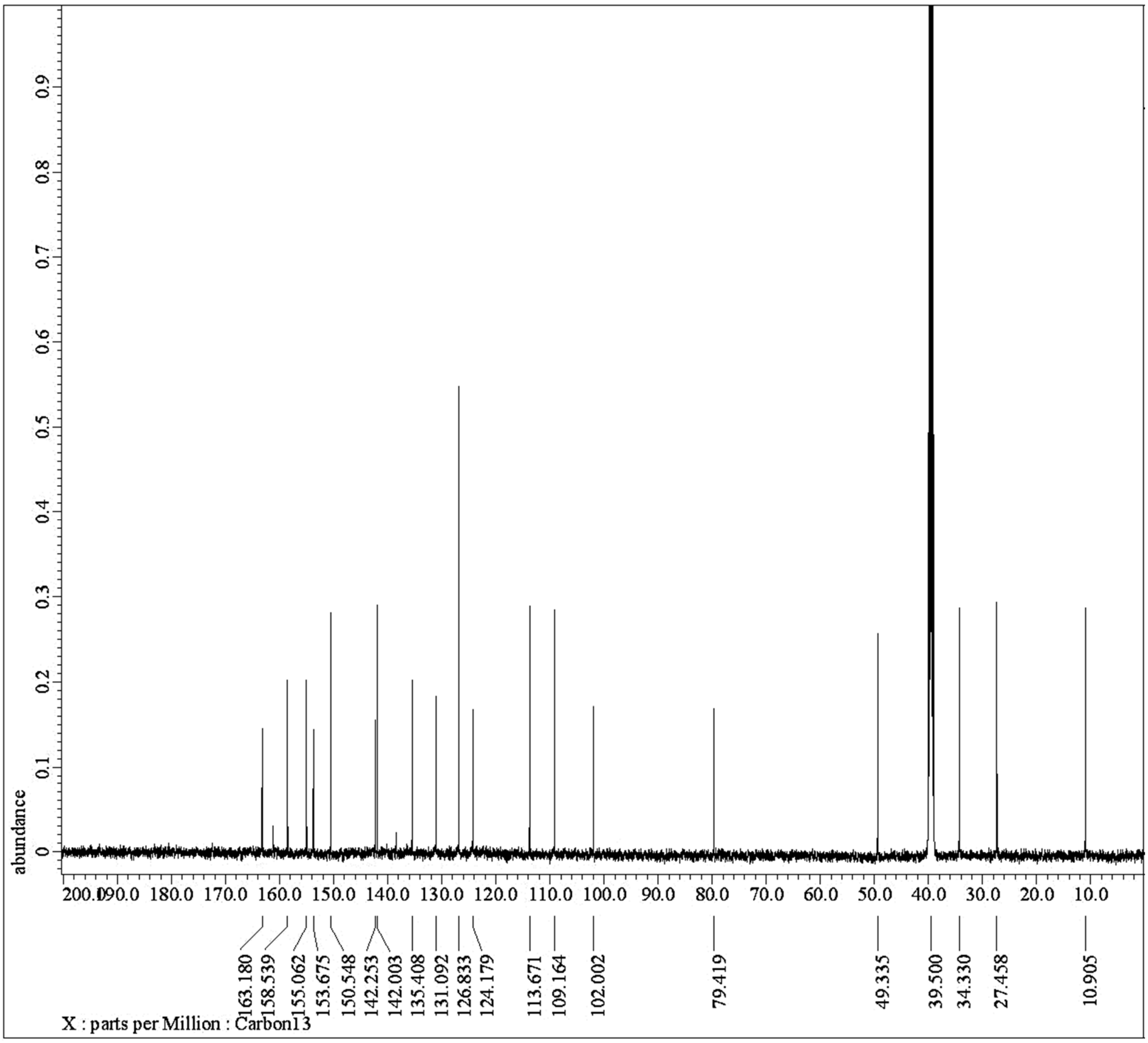

^{13}C NMR of Compound **8**

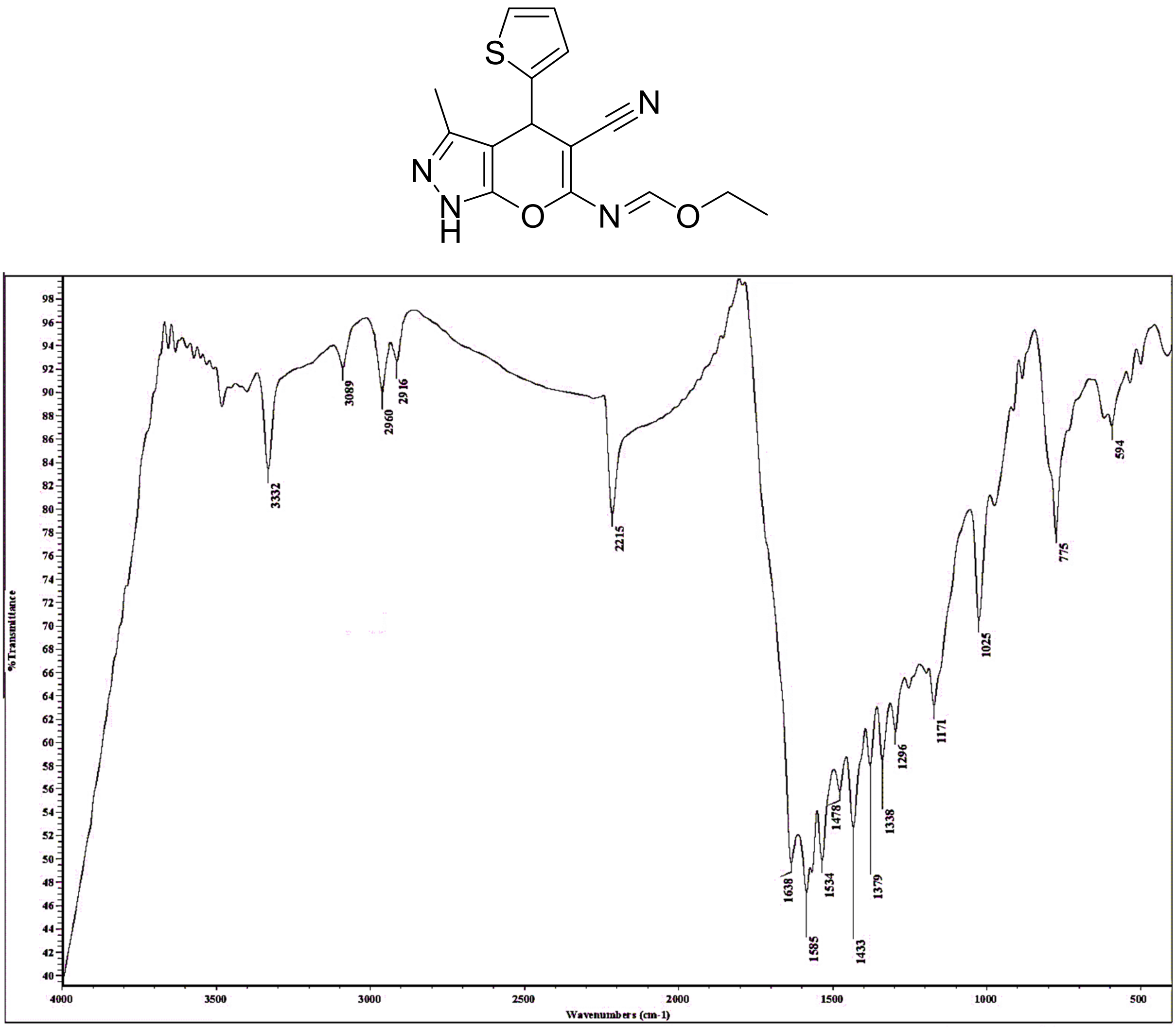

FT-IR of Compound **9**

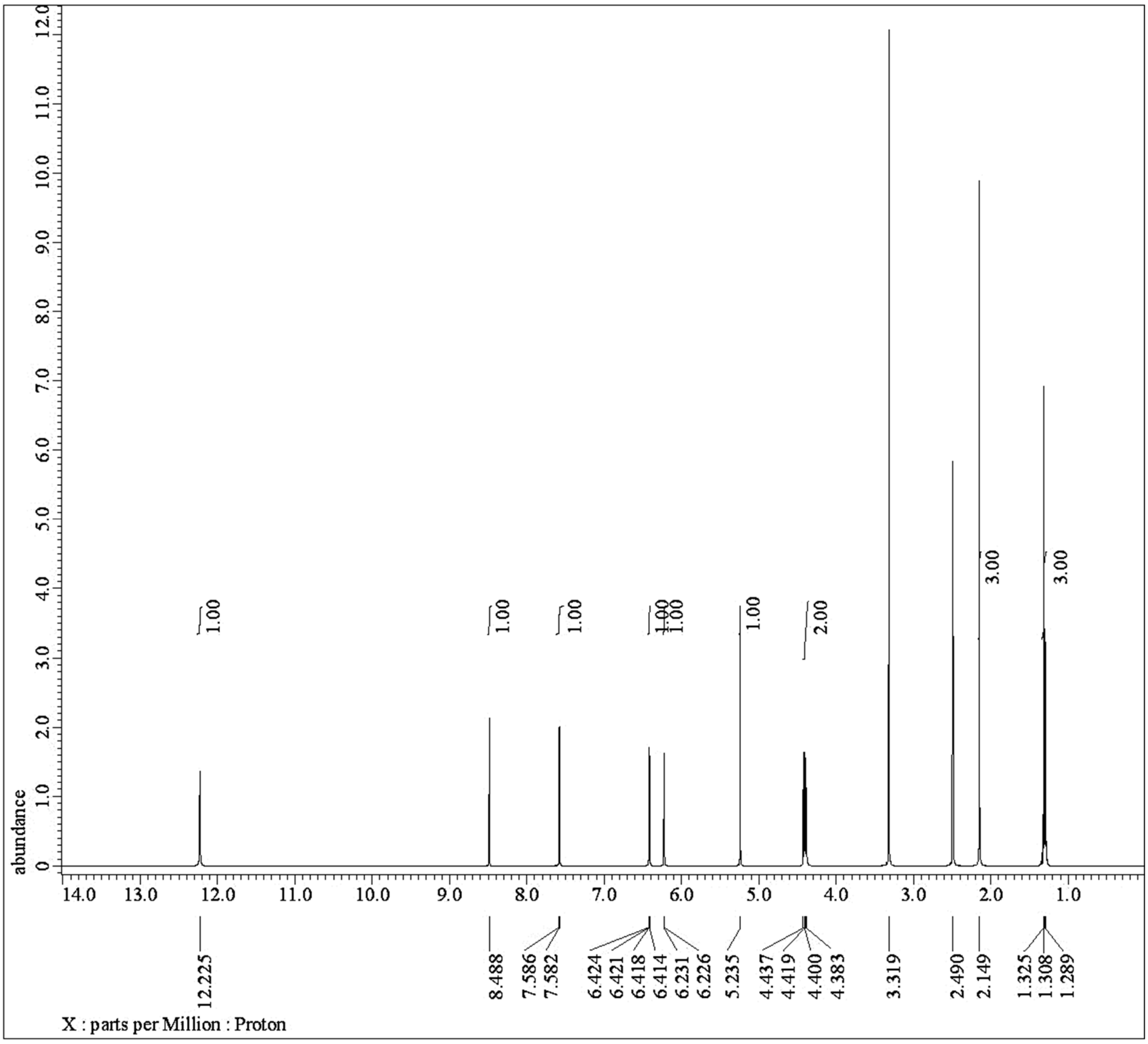

^{1}H NMR of Compound **9**

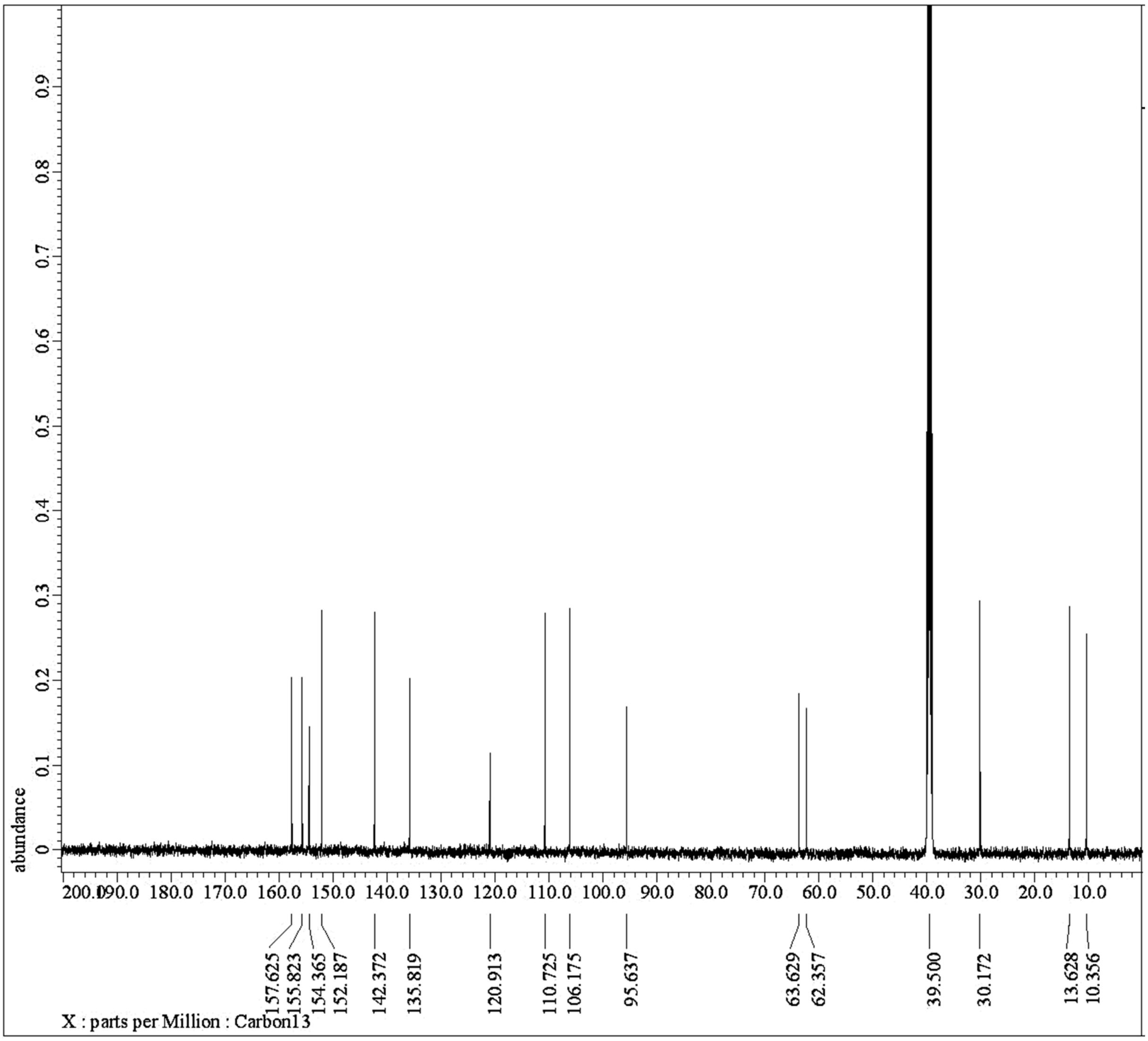

^{13}C NMR of Compound **9**

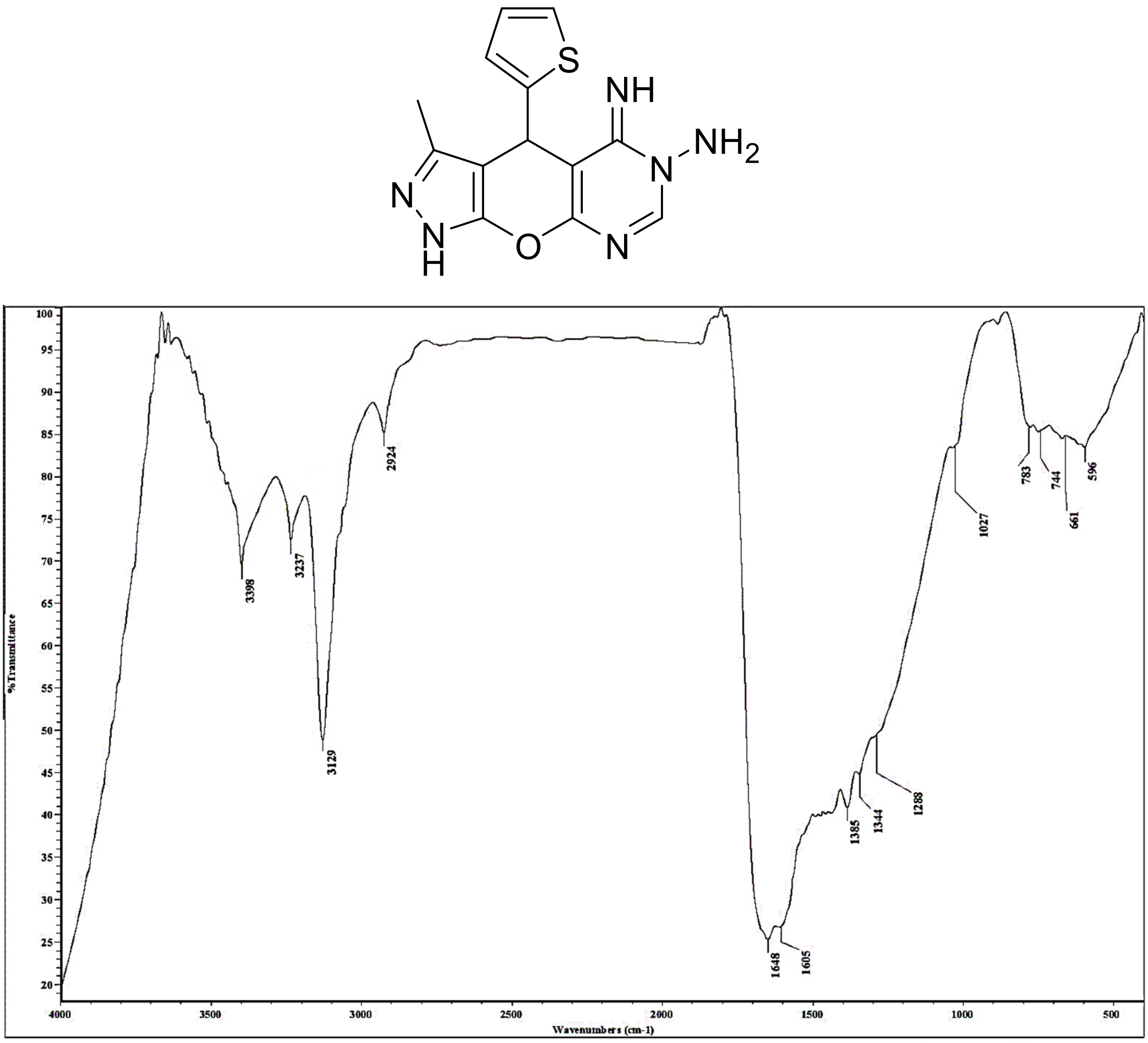

FT-IR of Compound **10**

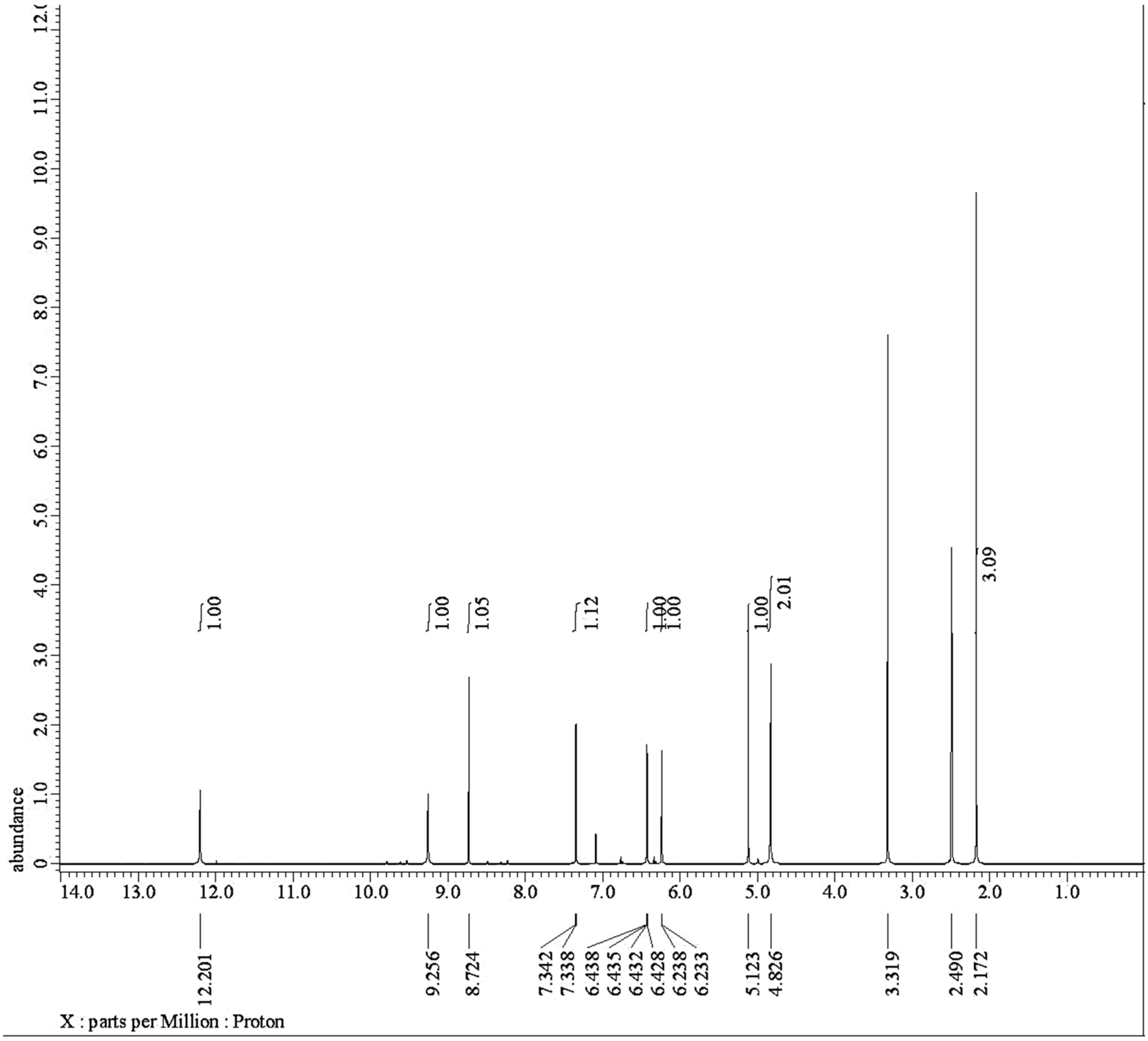

^{1}H NMR of Compound **10**

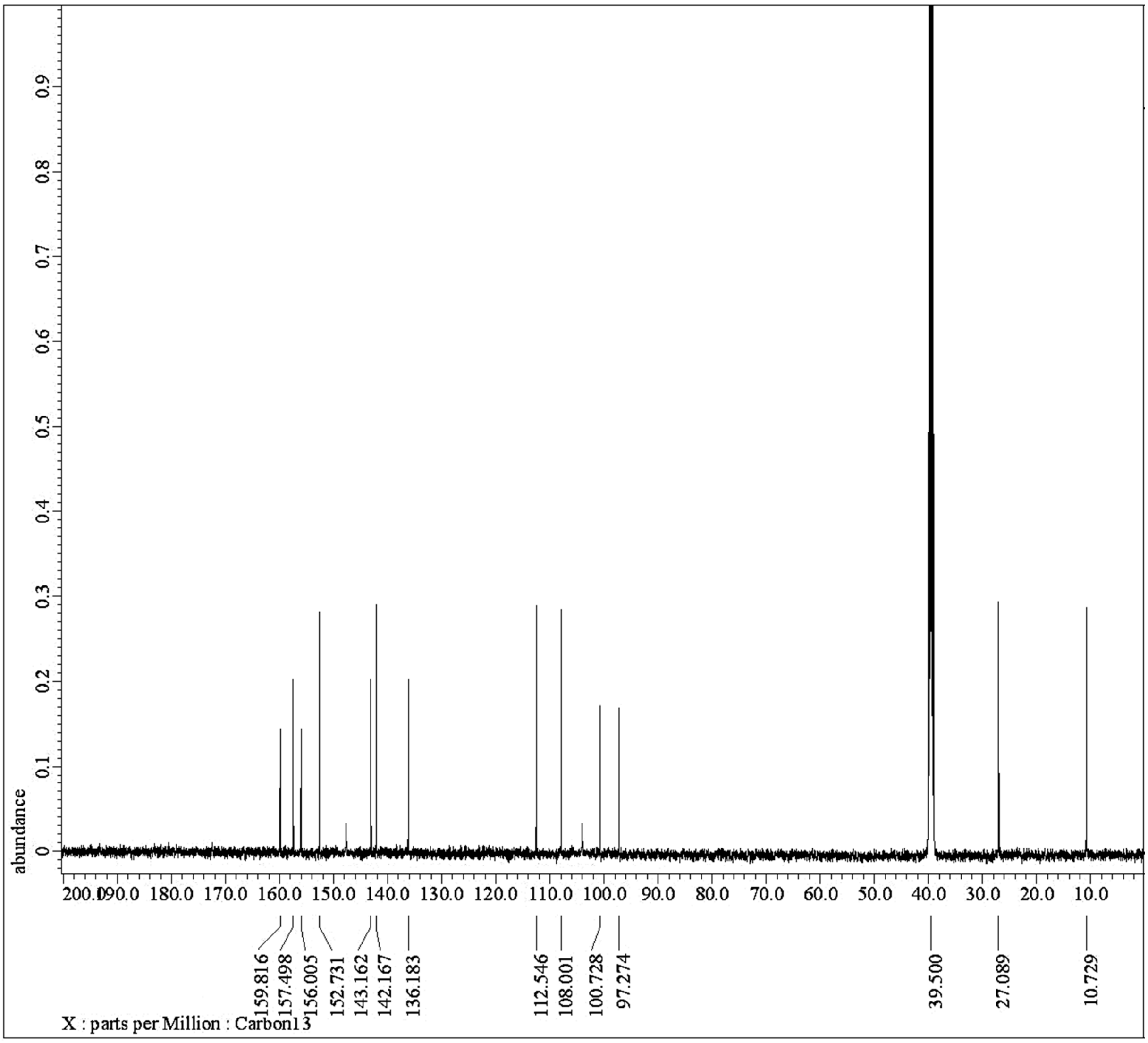

^{13}C NMR of Compound **10**

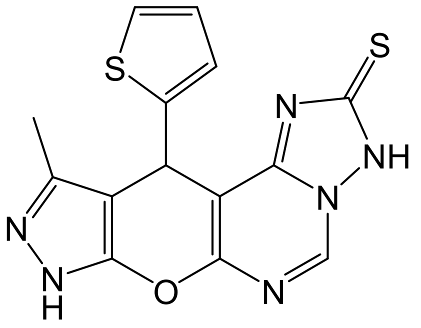

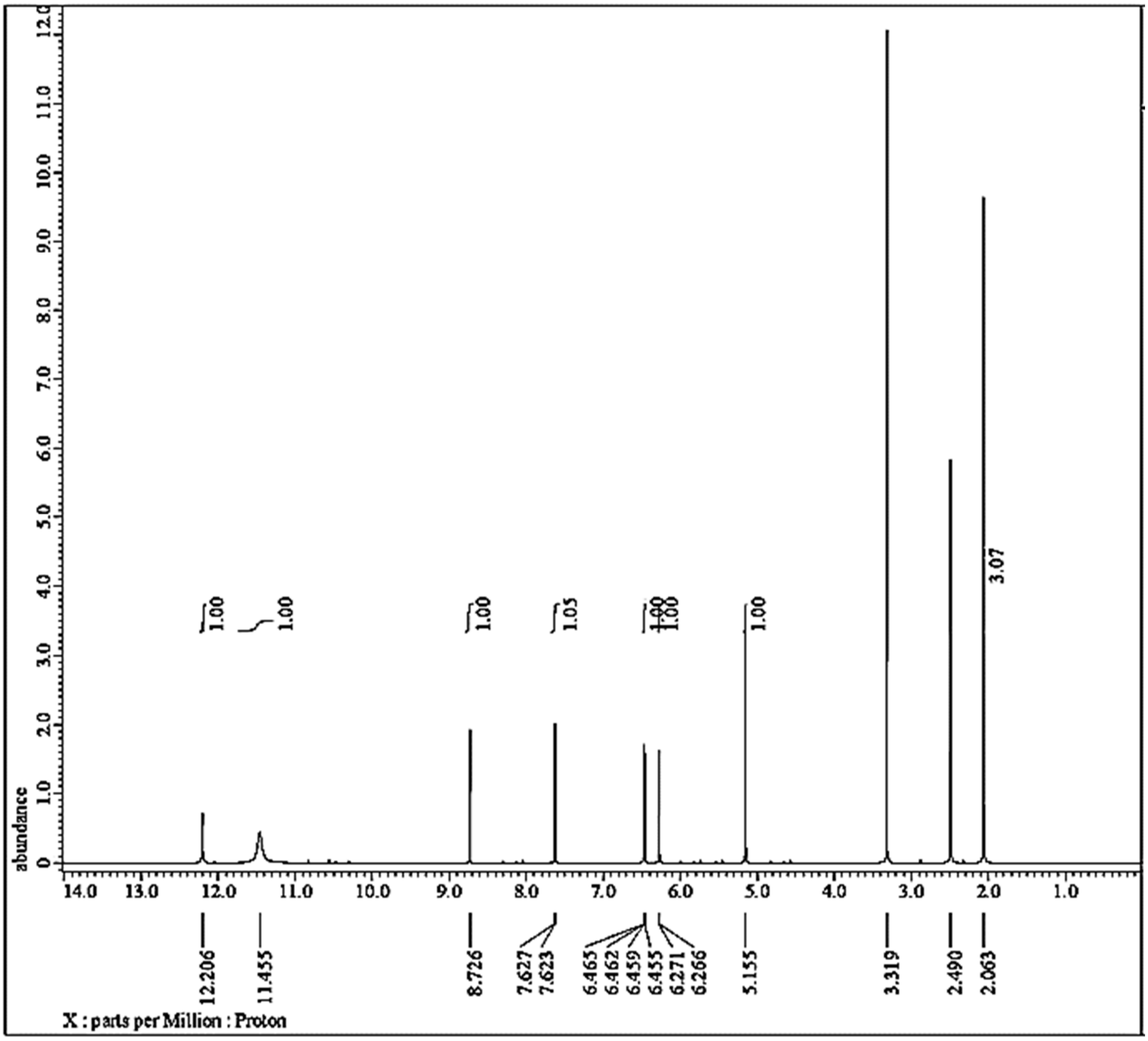

^{1}H NMR of Compound **11**

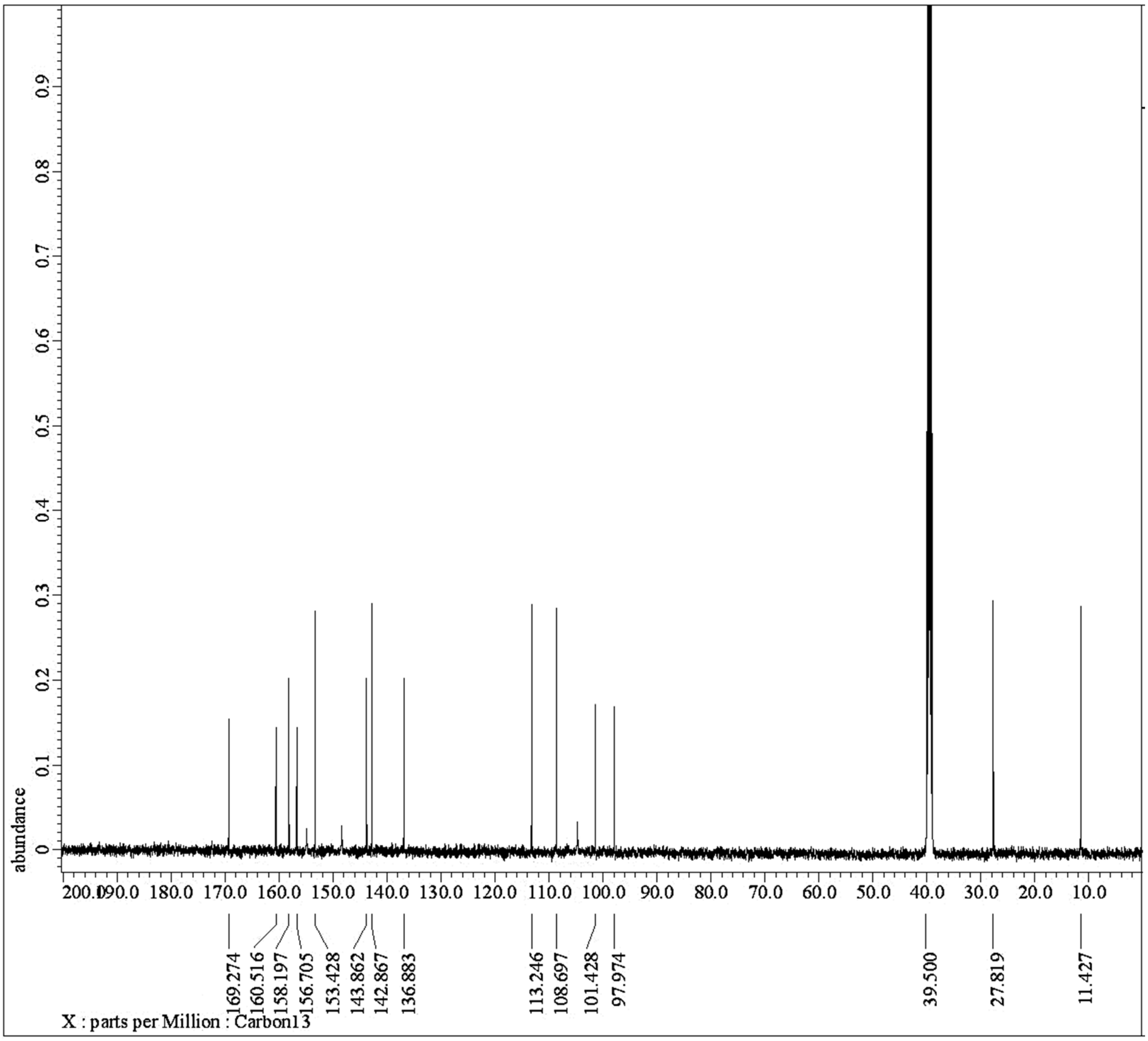

^{13}C NMR of Compound **11**

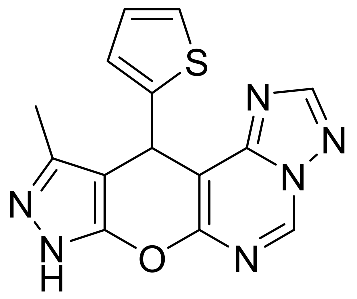

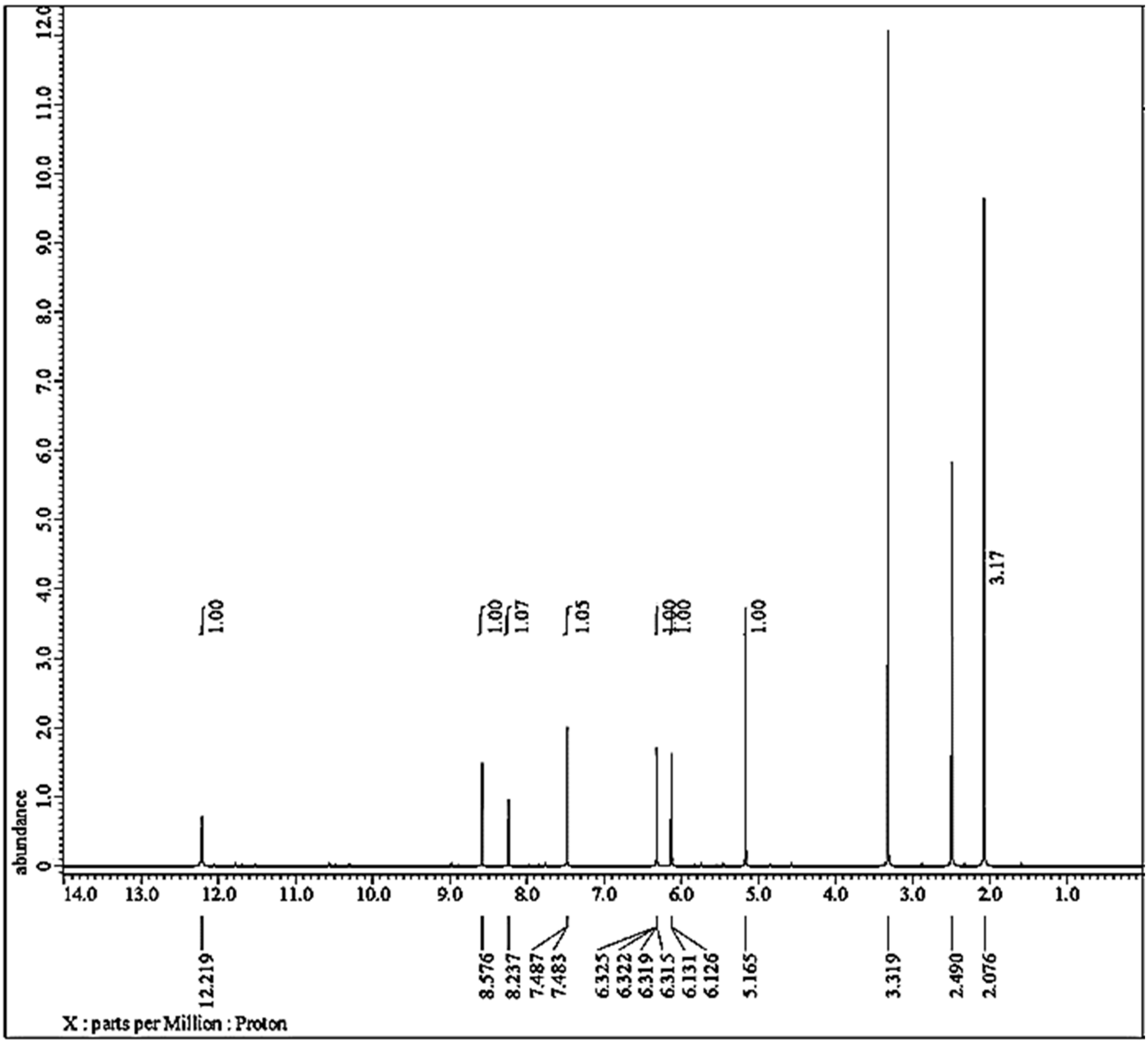

^{1}H NMR of Compound **12**

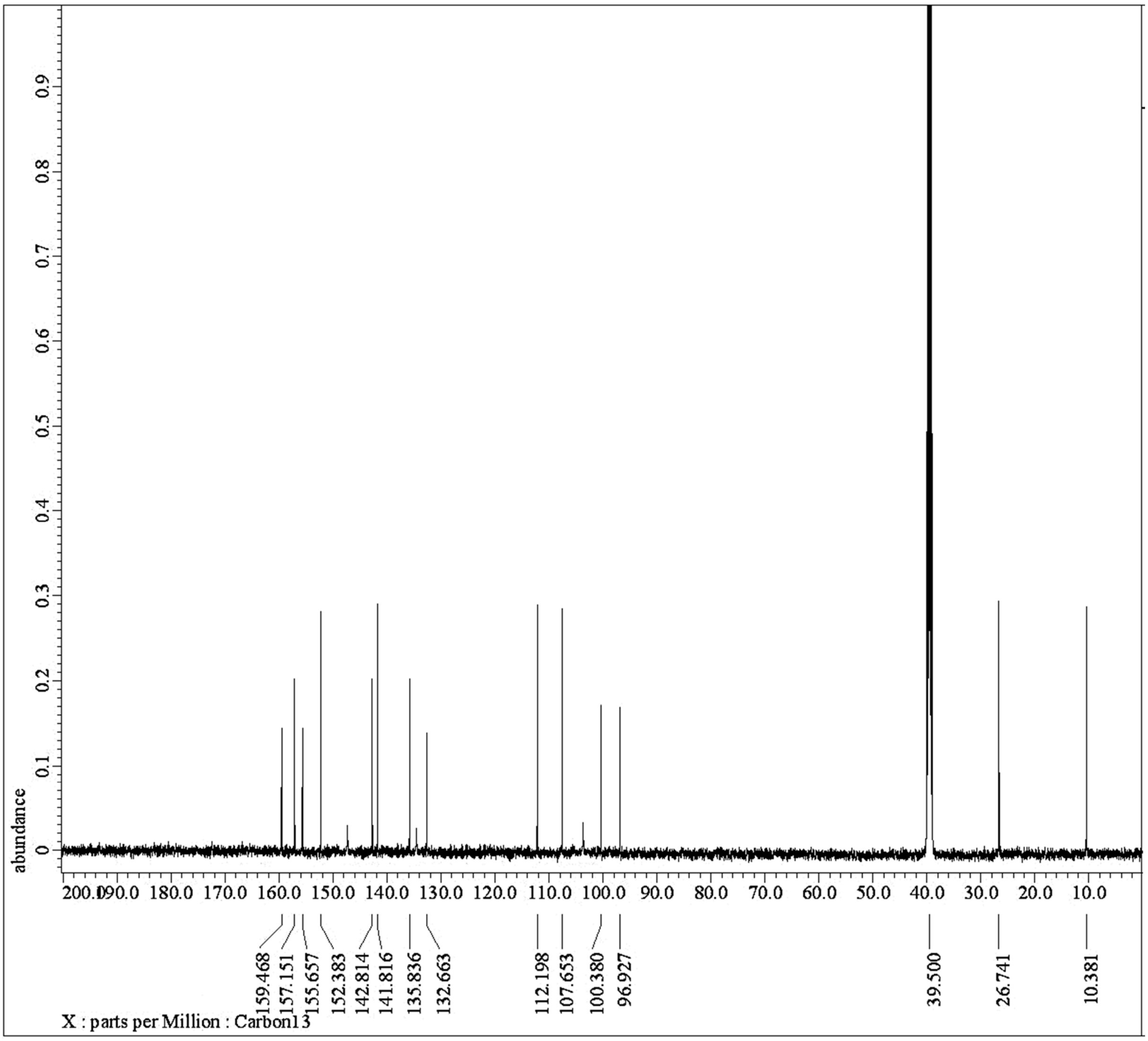

^{13}C NMR of Compound **12**

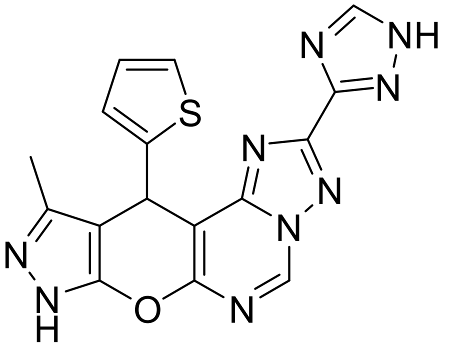

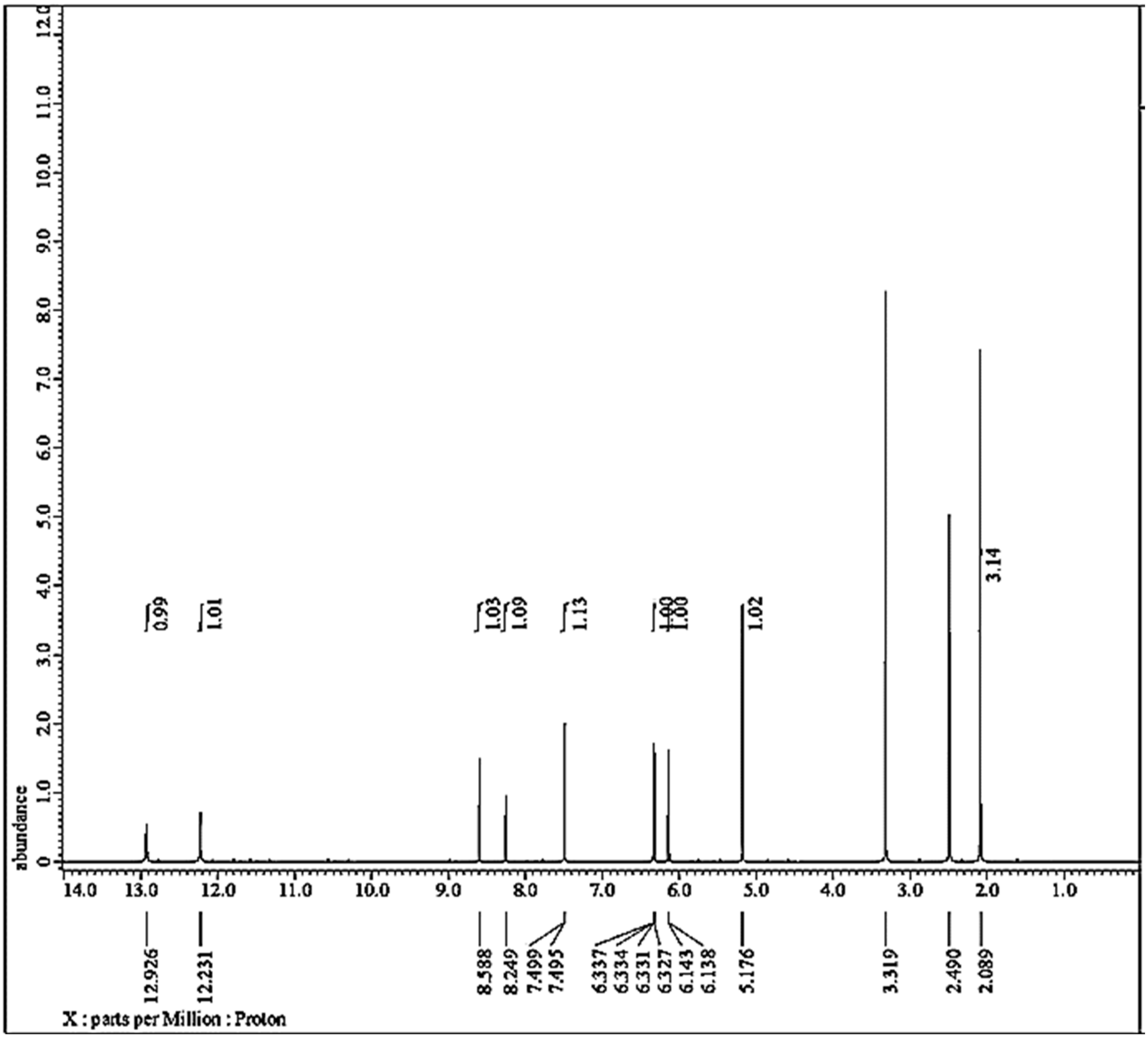

^{1}H NMR of Compound **13**

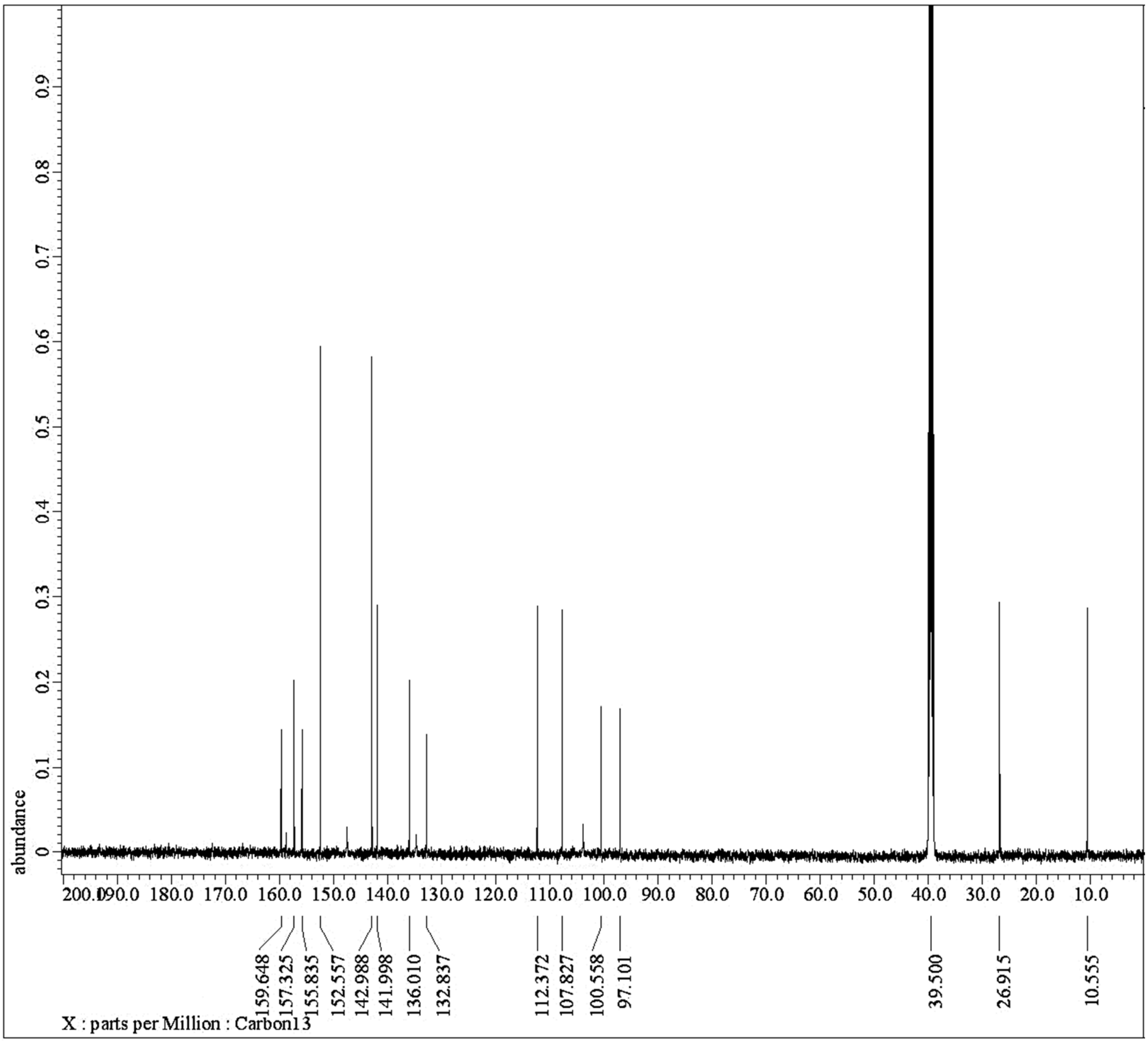

^{13}C NMR of Compound **13**

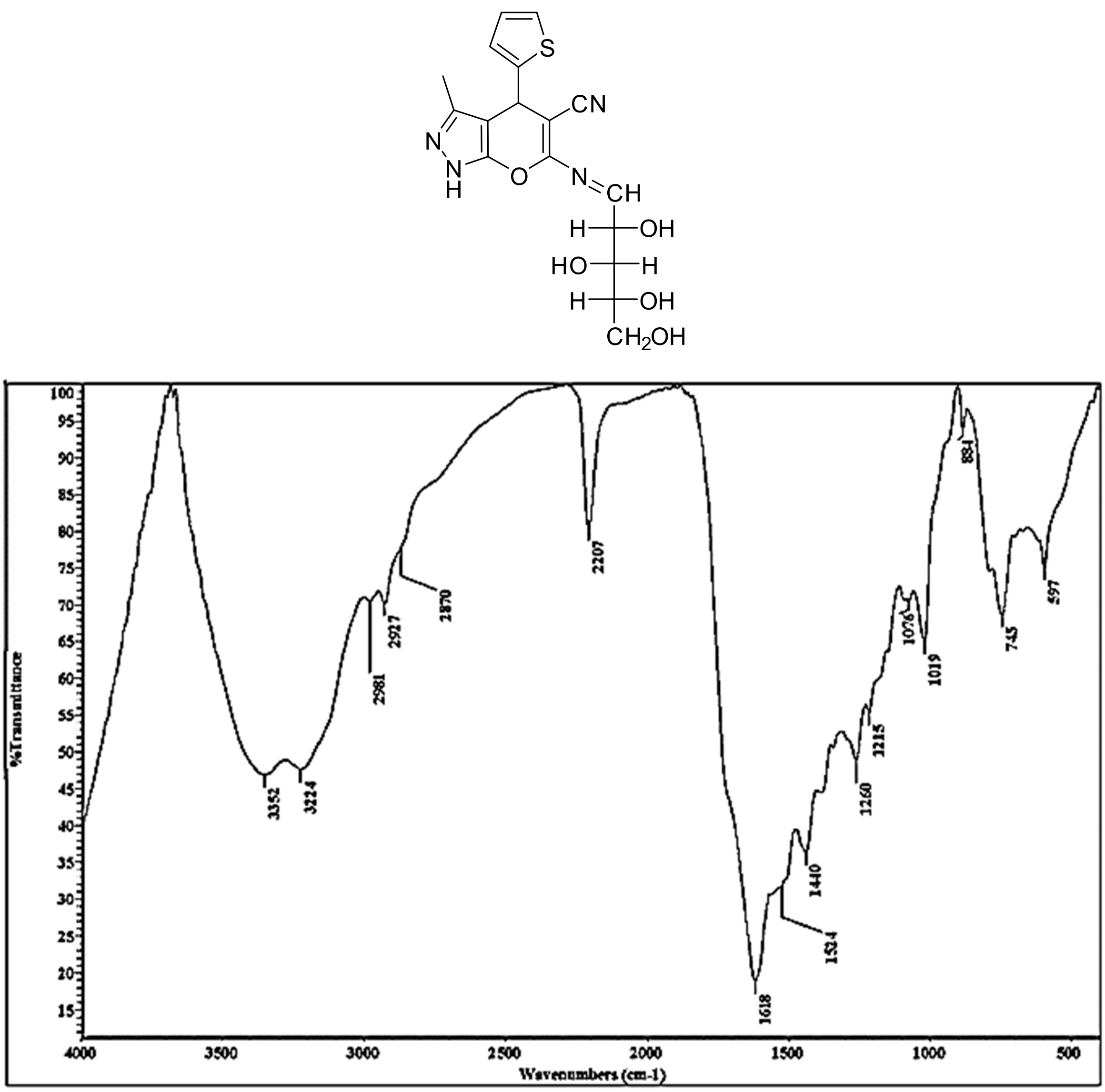

FT-IR of Compound **14**

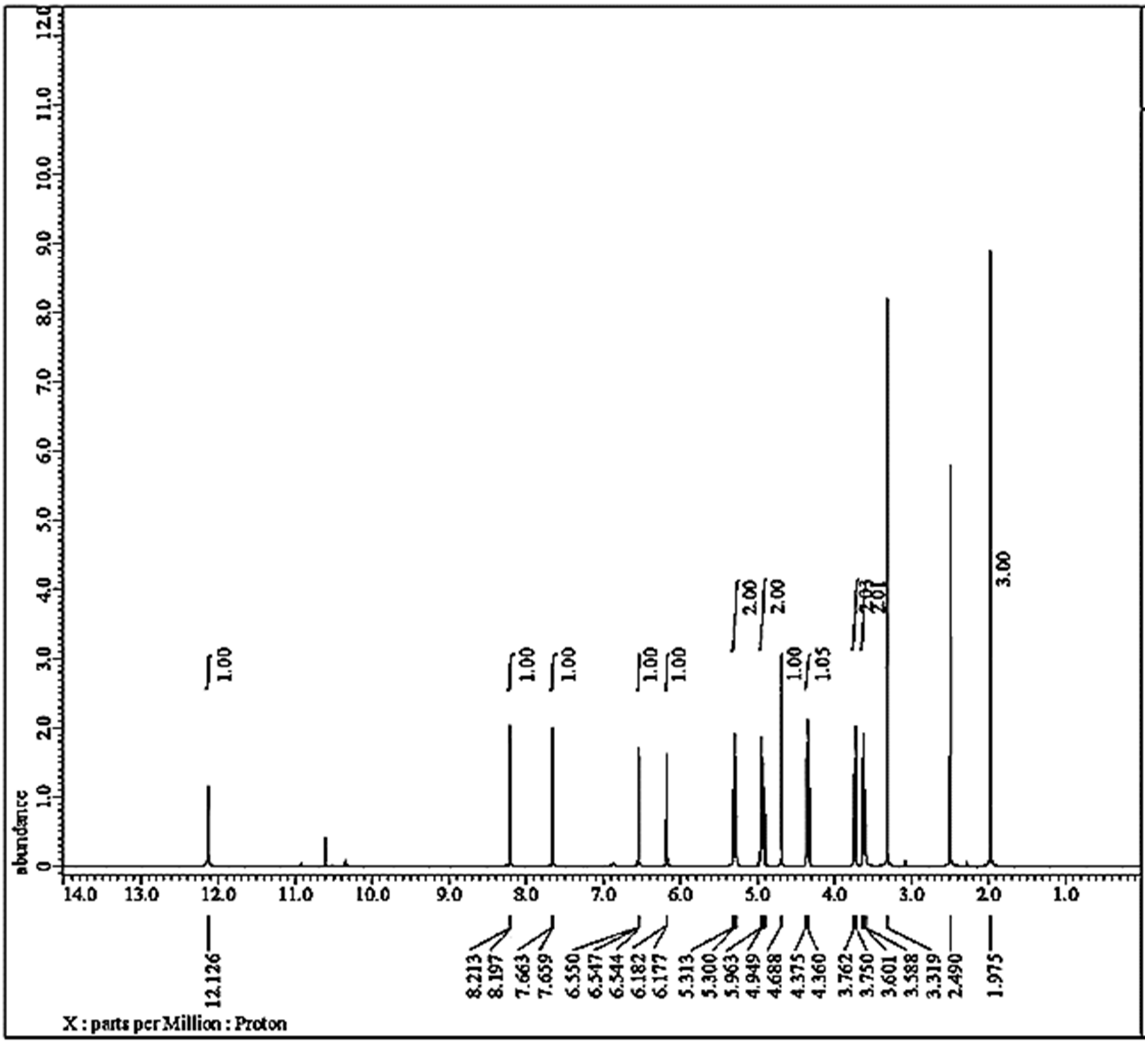

^{1}H NMR of Compound **14**

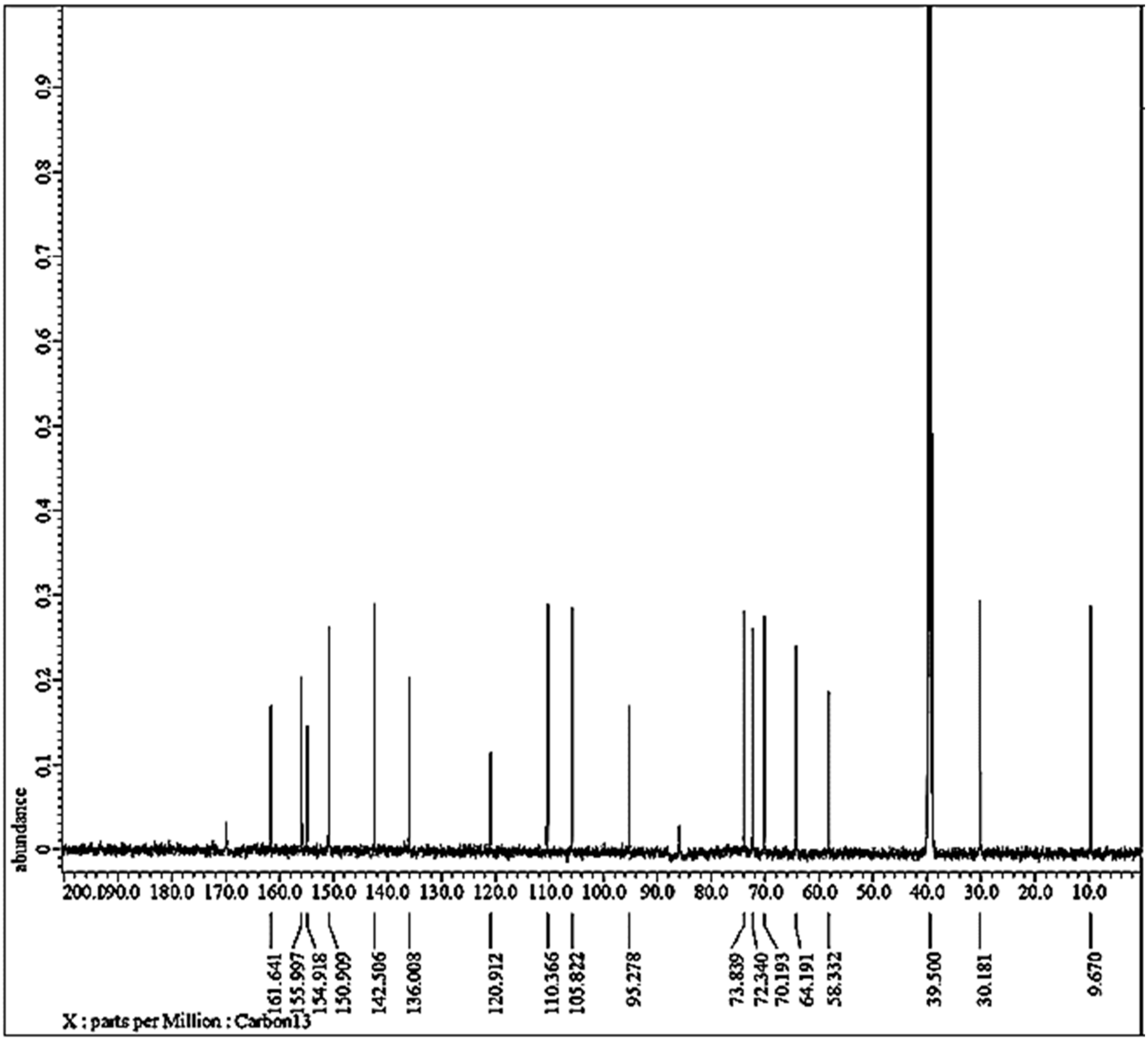

^{13}C NMR of Compound **14**

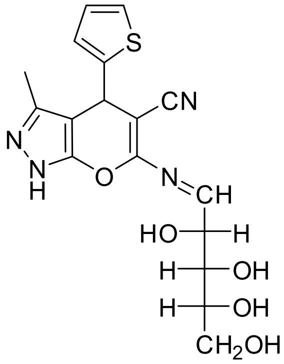

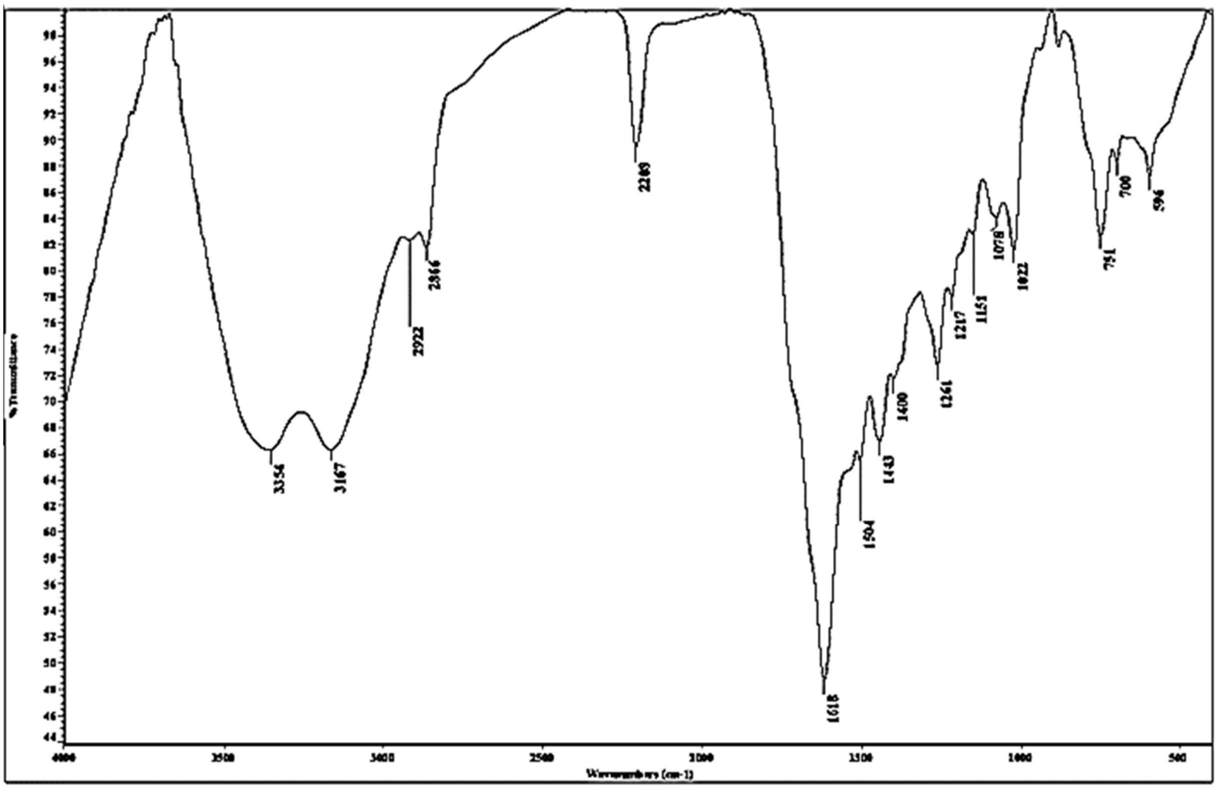

FT-IR of Compound **15**

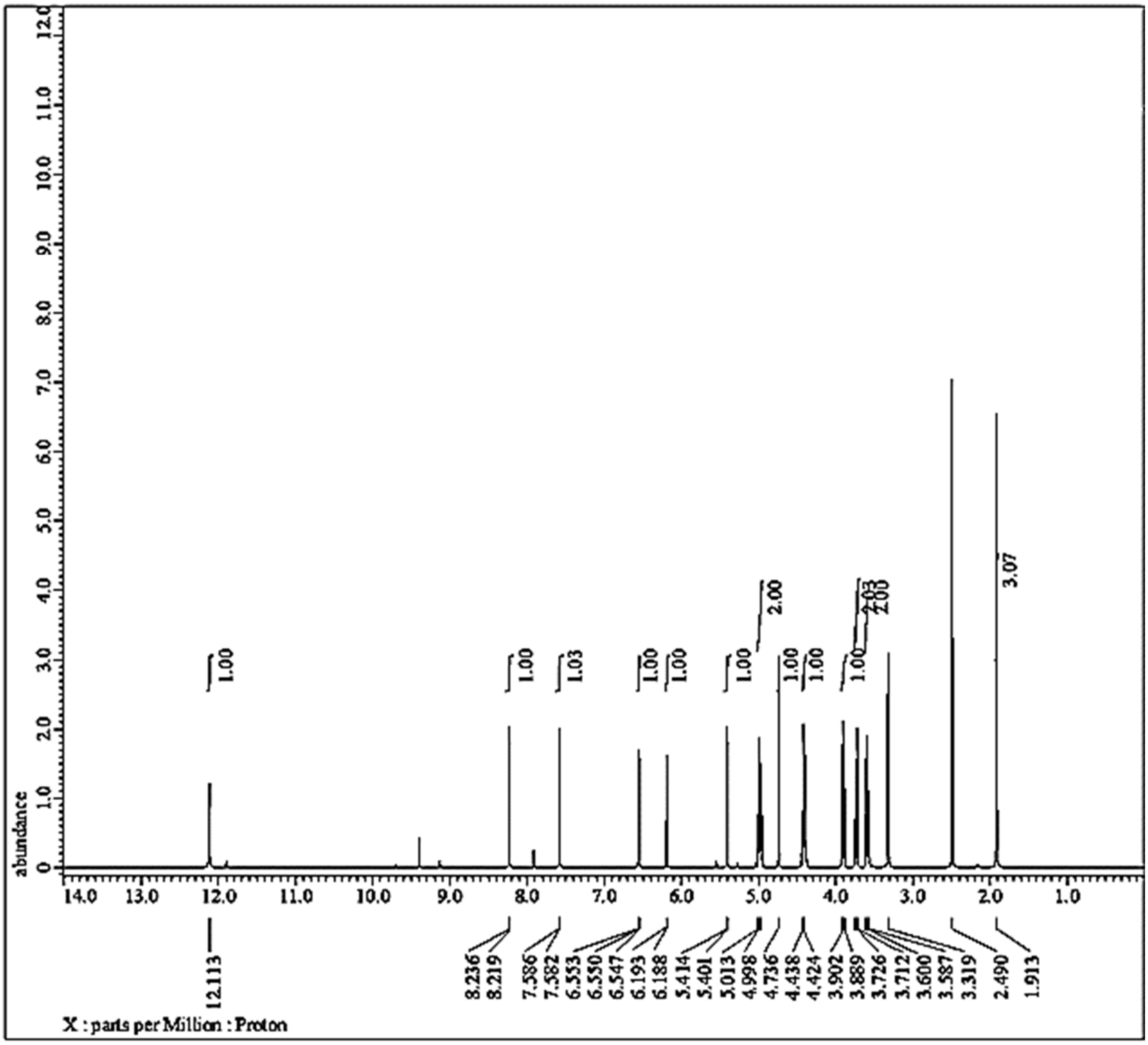

^{1}H NMR of Compound **15**

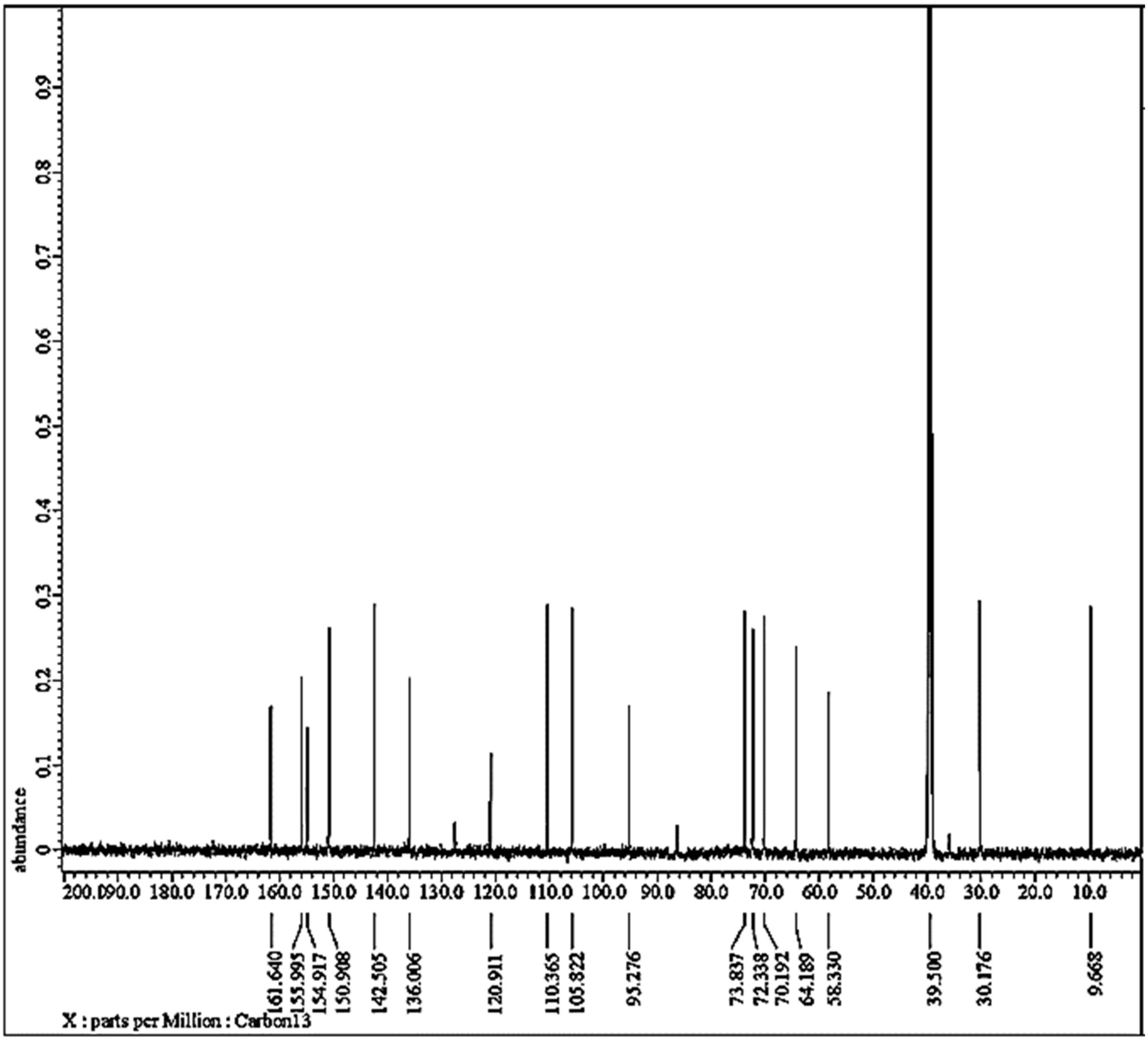

^{13}C NMR of Compound **15**

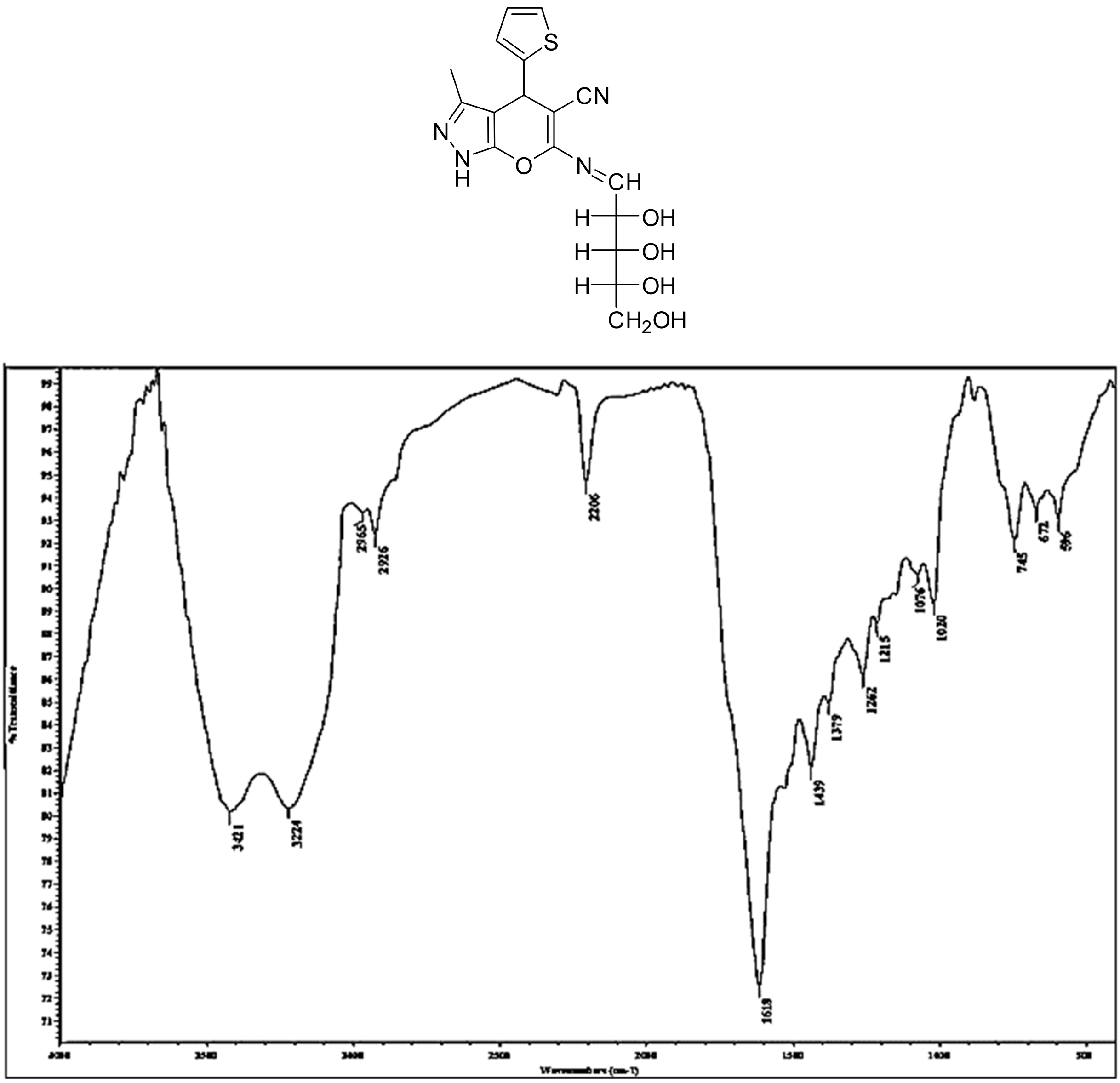

FT-IR of Compound **16**

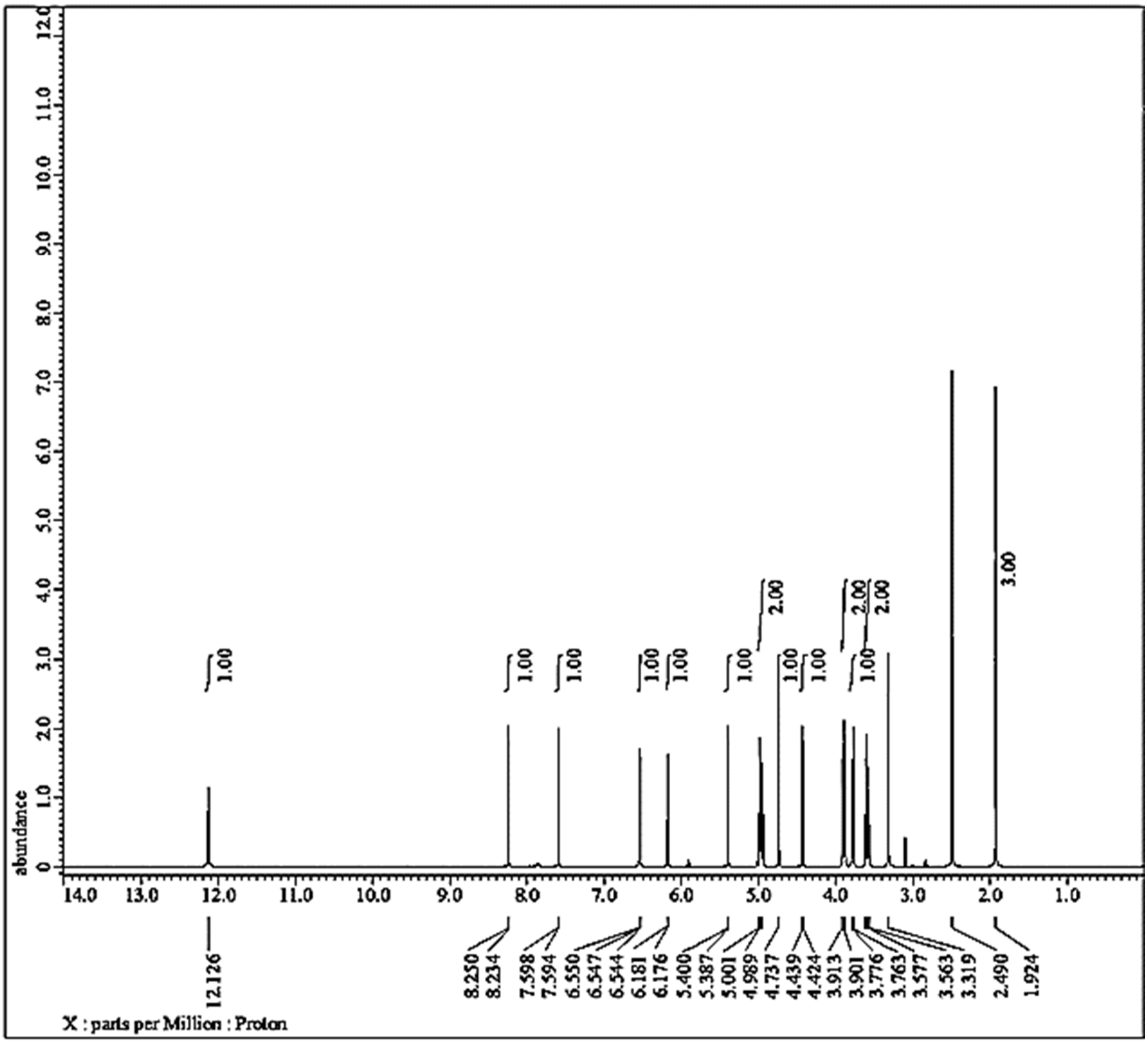

^{1}H NMR of Compound **16**

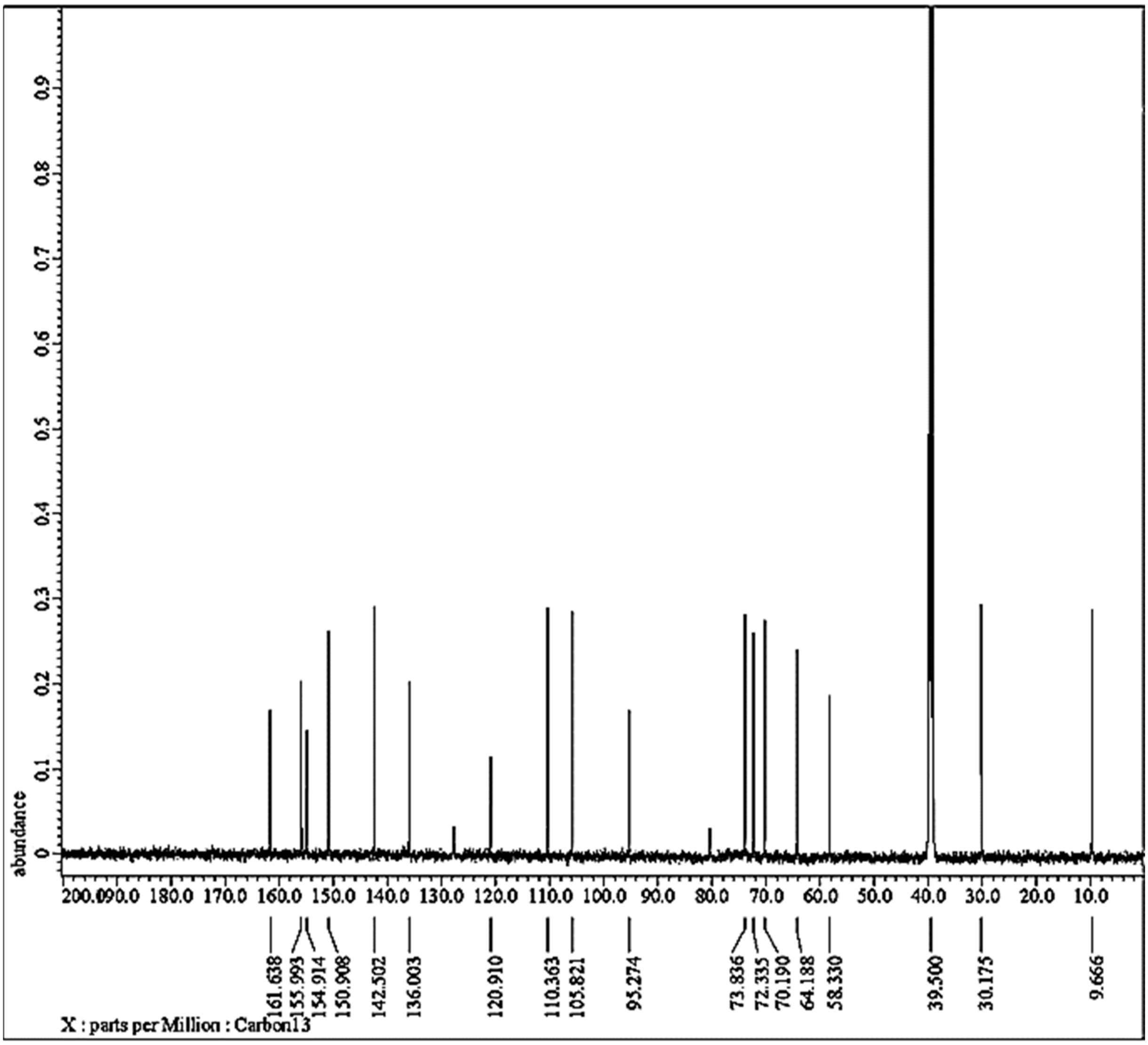

^{13}C NMR of Compound **16**

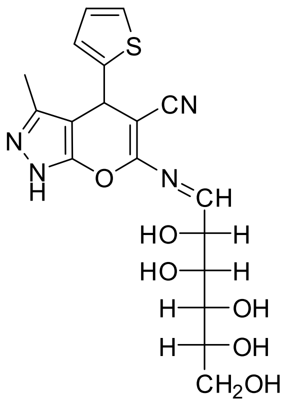

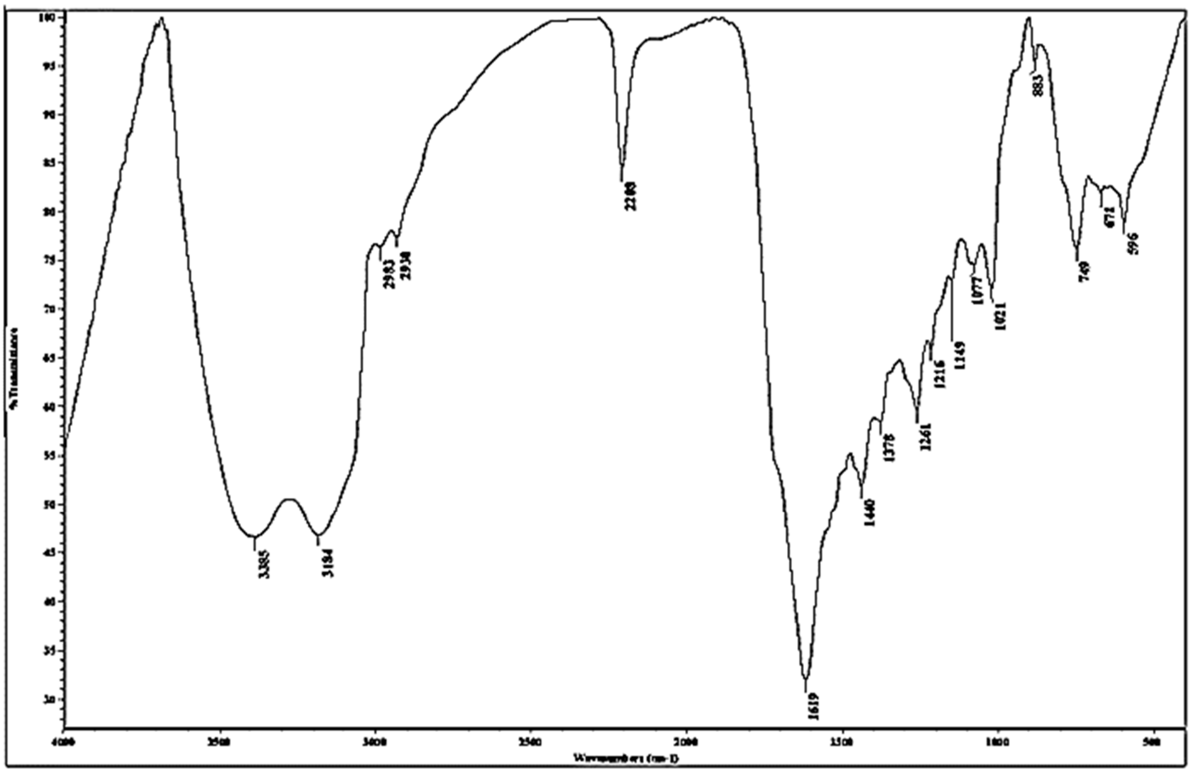

FT-IR of Compound **17**

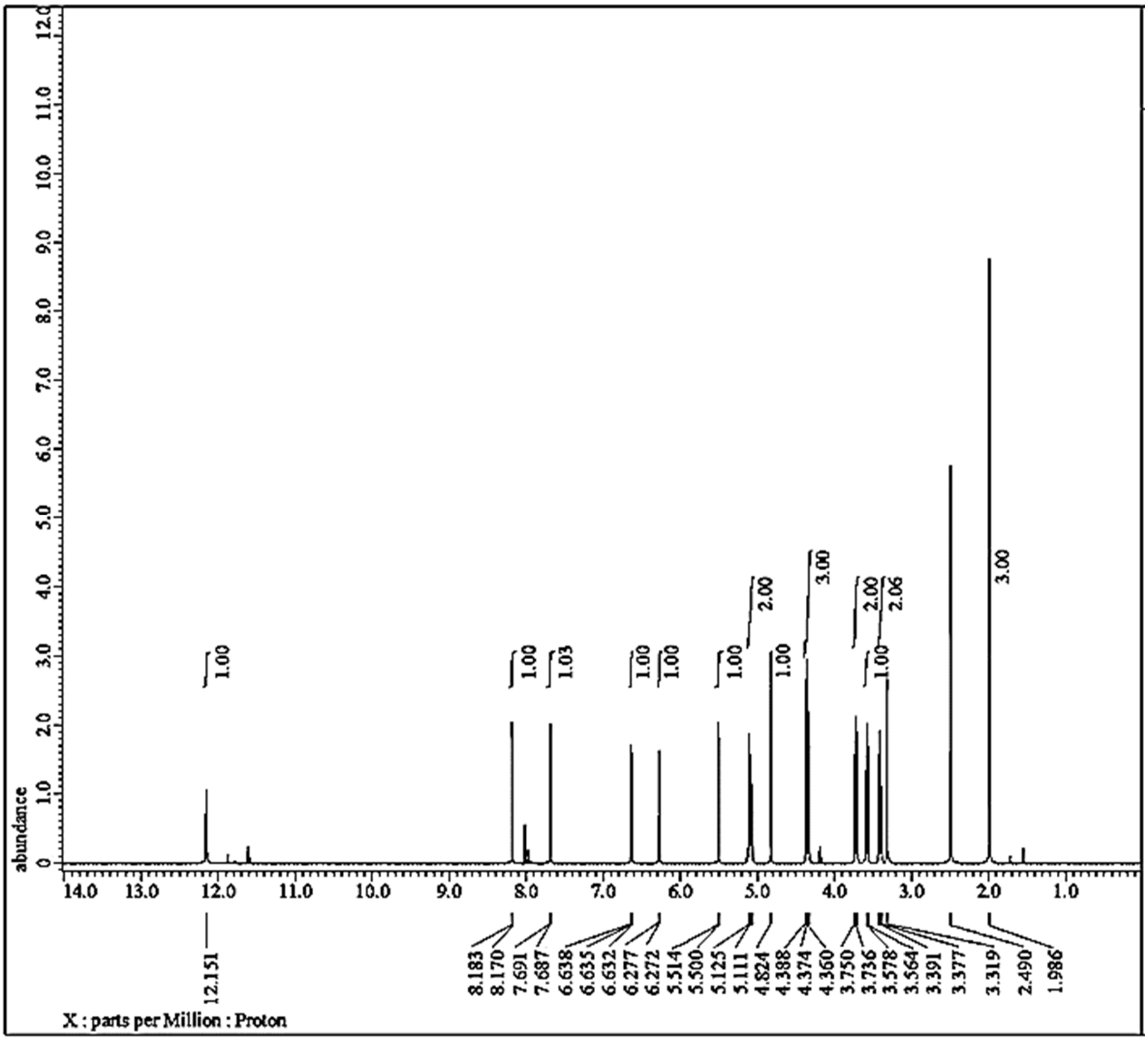

^{1}H NMR of Compound **17**

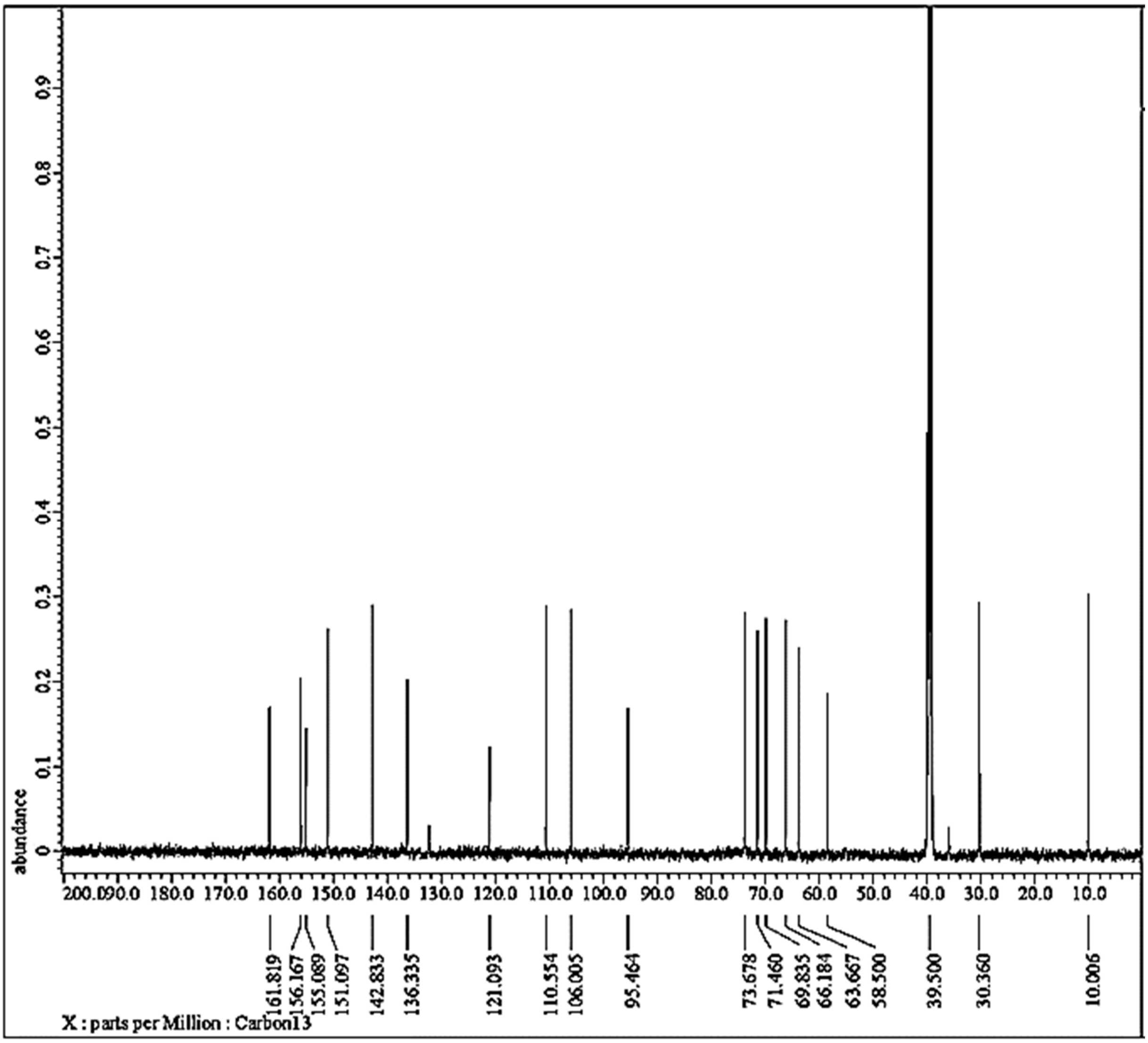

^{13}C NMR of Compound **17**

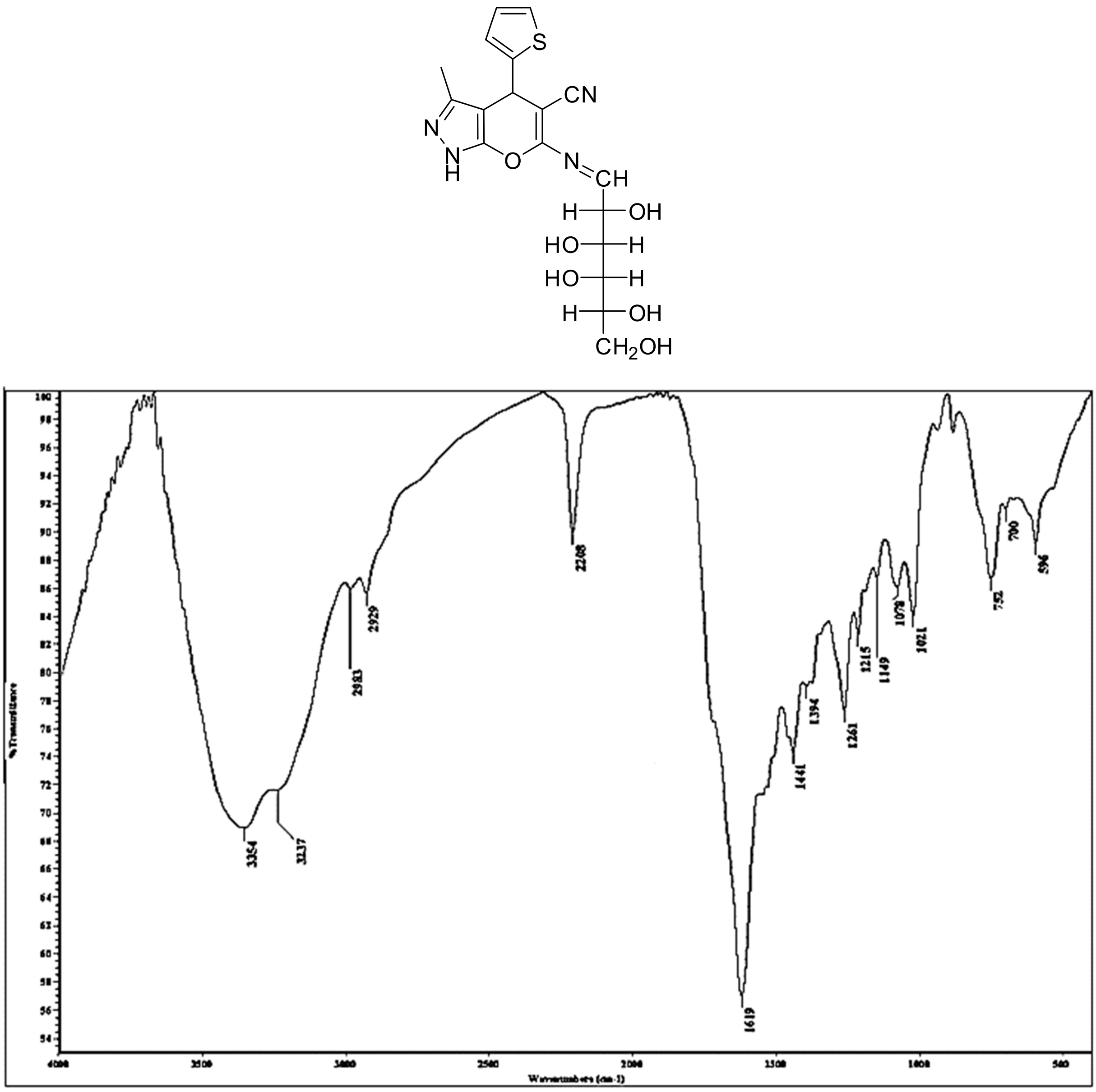

FT-IR of Compound **18**

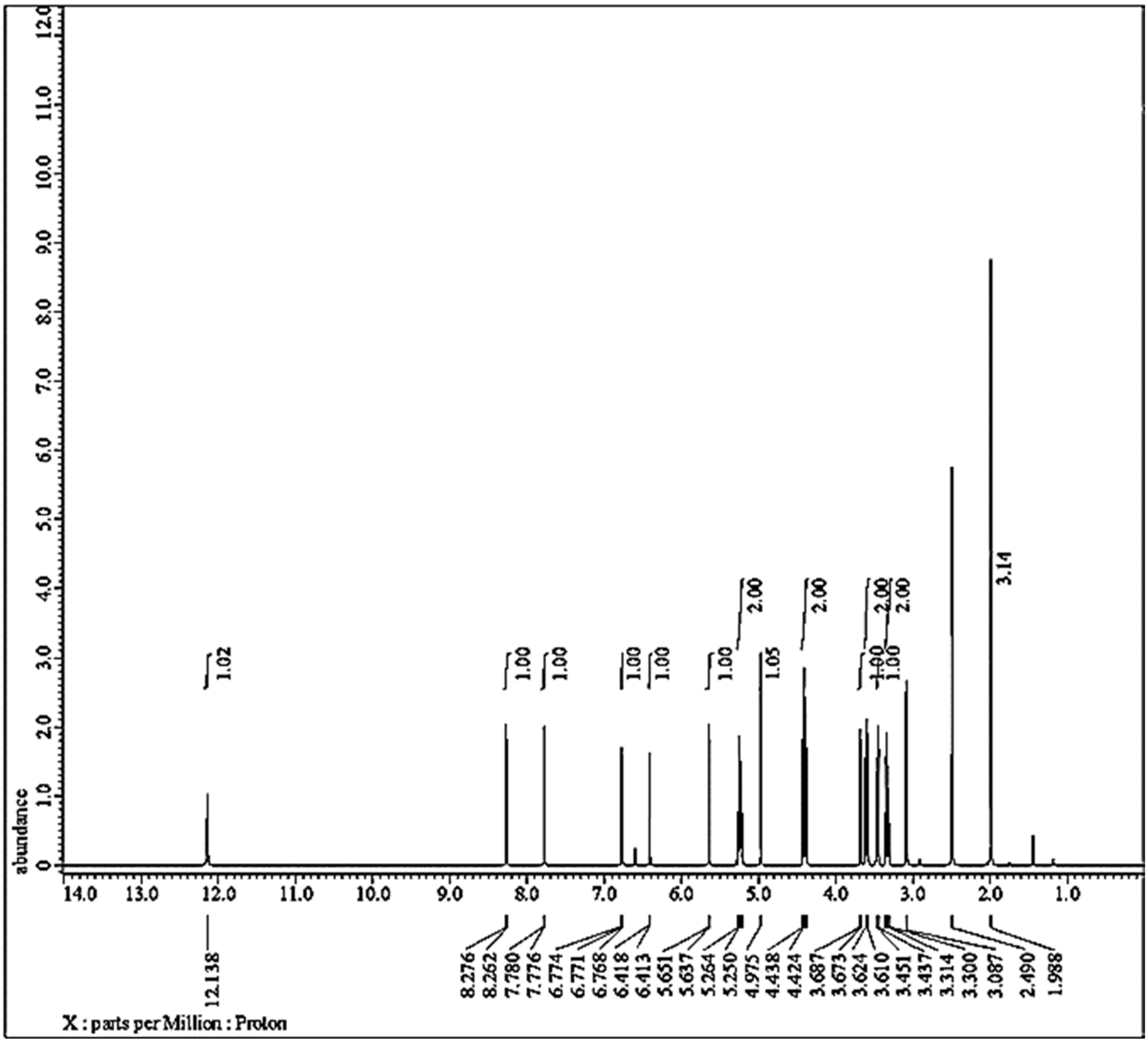

^{1}H NMR of Compound **18**

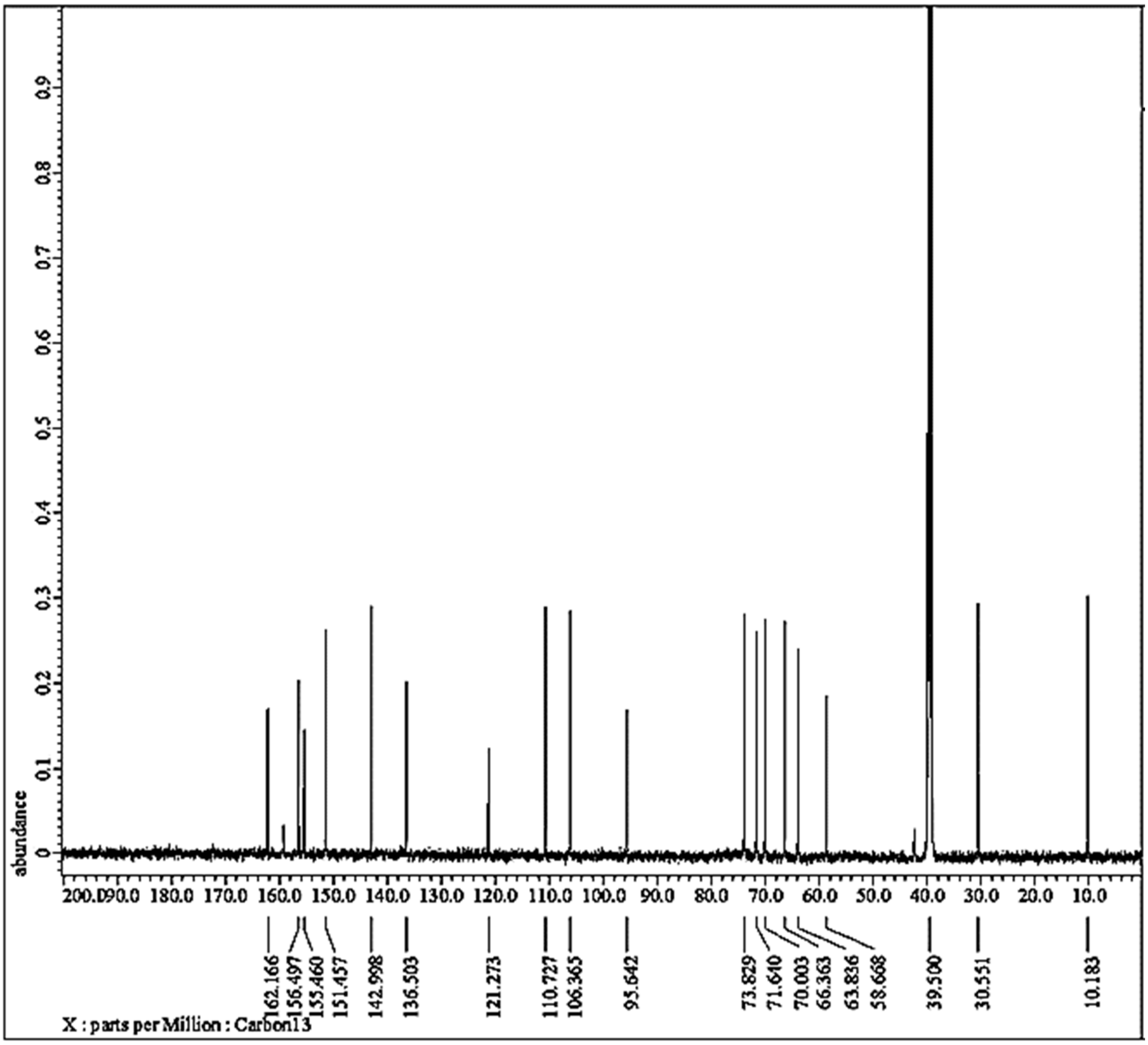

^{13}C NMR of Compound **18**
